# Beyond domestication: The unexpected contribution of native *Musa* wild relatives to cultivated banana diversity in Mainland Southeast Asia

**DOI:** 10.1371/journal.pone.0352247

**Published:** 2026-08-17

**Authors:** Xavier Perrier, Gabriel Sachter-Smith, Catherine Breton, Ronan Rivallan, Jean-Pierre Jacquemoud-Collet, Cécile Dubois, Julie Sardos, Frédéric Bakry, Matthieu Chabannes, Ngọc-Sâm Lý, Thomas Haevermans, Tiến-Dũng Triệu, Oudomphone Insisiengmay, Ting Zhang, Marie-Line Iskra-Caruana, Christophe Jenny

**Affiliations:** 1 CIRAD, UMR AGAP Institut, Montpellier, France; 2 UMR AGAP Institut, Univ Montpellier, CIRAD, INRAE, Institut Agro, Montpellier, France; 3 Hawaii Banana Source, Ohau, Hawai`i, United States of America; 4 Bioversity International, Parc Scientifique Agropolis II, Montpellier, France; 5 Institute of Life Sciences, Vietnam Academy of Science and Technology, 9/621 Vo Nguyen Giap, Linh Xuan ward, Ho Chi Minh City, Vietnam; 6 Institut de Systématique Évolution Biodiversité (ISYEB), Muséum national d’histoire naturelle, Centre national de la recherche scientifique, École Pratique des Hautes Études, Université des Antilles, Sorbonne Université, Paris, France; 7 Northern Mountainous Agriculture and Forestry Science Institute, Phú Hộ, Vietnam; 8 The Cabinet of the Lao Academy of Science and Technology, Ministry of Education and Sport, Vientiane Capital, Lao PDR; 9 Germplasm Bank of Wild Species, Kunming Institute of Botany, Chinese Academy of Sciences, Kunming, China; 10 CIRAD, Montpellier, France; South China Agricultural University, CHINA

## Abstract

The diversity of cultivated bananas remains partially unknown, whereas cultivars with suitable adaptive traits could address the drastically increasing biotic and abiotic constraints on banana production. This partial knowledge also impacts our understanding of domestication processes, although replicating these processes is an endorsed strategy for banana varietal improvement. To address this gap, 91 specimens were collected during a collecting mission across northern Vietnam, northern Laos, and China’s Yunnan region, an underexplored area at the northern limit of the *Musa* natural range. Native wild relatives were also collected (*Musa acuminata* subsp. *burmannica*, *M. balbisiana*, *M. itinerans, M. yunnanensis*) to assess their potential contributions to cultivated crops. SNP polymorphisms revealed that, beyond the globally cultivated sweet or starchy cultivars, farmers cultivate numerous previously undescribed landraces. SNP-based chromosome paintings further showed that all these landraces carry genomic signatures from endemic wild *Musa* relatives. Notably, *Musa acuminata* subsp. *burmannica*, typically regarded as a minor contributor to cultivated bananas, was detected in several *M. acuminata* derived diploids (AA) and triploids (AAA) as well as *M. acuminata* × *M. balbisiana* derived AAB and ABB landraces. Remarkably, we also report here for the first time polyploid landraces containing a complete haplotype of the local *M. itinerans*. Crucially, we showed that these landraces were not directly domesticated from the endemic wild *Musa*. They appear to result from local hybridizations of endemic wilds with exogenous AA diploids introduced into Mainland Southeast Asia from distant regions and originating from earlier domestication farther south. In addition, the various *M. acuminata* subspecies contributing to these exogenous diploids, provide arguments for an alternative eastern route of introduction via southern China or Taiwan and through the Philippines, distinct from the previously described western route via the Malay Peninsula, through Java, and Sumatra. We highlight an accumulation of genomic introgressions from native wild relatives, suggesting that banana diversity was shaped by an atypical process of cumulative diversification. This ‘post-domestication’ process is thought to enhance local adaptation through genes pyramiding. This multi-level genetic stacking is strengthened in bananas by polyploidy and vegetative propagation fixing any valuable combinations.

## Introduction

Bananas are a key crop in all humid subtropical regions, with more than 140 million tonnes produced worldwide. While industrial banana cultivation supplies the international sweet banana market, bananas are primarily a staple food across vast regions, with home consumption and local market trade accounting for nearly 80% of global production [1].

Cultivated banana varieties are seedless with well-developed fruit pulp, they emerged through domestication from wild forms via progressive selection for sterility and parthenocarpy, compensated by efficient vegetative propagation [2–4]. As a consequence of the necessary vegetative propagation, only a few genotypes are cultivated on a large scale. This results in limited adaptability to environmental changes and high vulnerability to plant health risks, which have been significantly amplified by global exchanges.

Therefore, developing adapted and resistant varieties is imperative. This requires thorough knowledge of the available diversity, both to preserve it for future use and to identify the adaptive traits essential for addressing the increasing biotic and abiotic constraints.

The ancestors of nearly all cultivated bananas belong to the *Musa* section of the genus *Musa*, family *Musaceae*. Species in this section are distributed across a vast area extending from India to South China and Taiwan, and southward to Papua New Guinea and northern Australia. The Fehi group cultivated in Oceania, which was domesticated from the *Callimusa* section, falls outside the scope of this study.

Among these species, *Musa acuminata* (A genome) is present in all cultivated bananas. The first domesticated forms were AA diploid varieties, and some of them are still widely cultivated in their regions of origin, in Southeast Asia and the Western Pacific. Several subspecies have been identified within wild ancestral forms of *M. acuminata* [5–7], they result from differentiation by geographical isolation within the highly insular Southeast Asian region [8–10]. This differentiation has been accompanied by several genomic translocations [11,12]. The AA diploid varieties were then shown to be intersubspecific hybrids [13,14], resulting from crosses between subspecies that, when naturally isolated, were brought into contact through human migration in the region [15]. Note that for clarity, species and subspecies names will often be used hereafter in their own as substantive nouns.

Three *M. acuminata* subspecies – banksii, zebrina and malaccensis – are considered the main contributors, with burmannica subspecies being more marginal. However, two other unknown *M. acuminata* genetic pools have recently been suggested to have contributed to cultivated AA [16], one being possibly the halabanensis subspecies, largely reported in Sumatra where it is sympatric with malaccensis subspecies [17]. A third pool originating from northern Borneo has also been proposed [10,18].

Interspecific hybridisation of these diploid AA varieties with *M. balbisiana* (B genome) occurred locally in sympatric areas, producing AB diploid cultivars, some of them are still cultivated today, especially in India. Other *Musa* species may have been involved, particularly *M. schizocarpa*, whose introgressions into A genomes suggest that spontaneous hybridisation with *M. acuminata* subsp. *banksii* occurred in their area of sympatry in Papua New Guinea. This initial hybridisation may have been a seminal factor in triggering the domestication process of all bananas [13,19].

Inter(sub)specific AA or AB diploid hybrids combine genomes that are sufficiently divergent to disrupt meiosis, leading to sterility but also to the formation of unreduced diploid gametes [20], which enable the emergence of triploid genomes with increased parthenocarpy and sterility [21,22].

These triploid varieties form cultivar groups [23] that have diversified phenotypically from initial sexual forms through diverse somaclonal processes of variation and fixation through vegetative propagation. These AAA, AAB, and ABB triploid varieties are the most widely cultivated forms today.

The study of this diversity, initially based on phenotypic traits, now relies on extensive genomic resources. The first genome to be assembled was a doubled haploid of ‘Pahang’, a *M. acuminata* subsp. *malaccensis* type [24], which was later updated [25]. Fully assembled genomes have also been published for *M. balbisiana*, *M. itinerans*, and other species [26]. These genomes are available on “The Banana Genome Hub” [27,28]. The complete sequences enabled locating numerous genome-wide single nucleotide polymorphisms (SNPs), opening the development of genotyping-by-sequencing (GBS) methodologies. These methods are well-suited for processing the large sample sets required for comprehensive diversity studies.

Building on pioneering works based on molecular markers [10,13,29–31], several recent studies, that will be addressed below, are based on in-deep characterisation of the genomes. They improved our understanding of the diversity of cultivated bananas as well as their wild relatives, clarifying the processes of diversification and domestication. Exploring this diversity is also a way to identify essential adaptive traits for addressing the increasing biotic and abiotic constraints faced today.

Most of these studies were conducted on representatives of the diversity available in major collections in Asia, Africa and the Caribbean. The *ex-situ* collection of the International Musa Germplasm Transit Centre (ITC) in Belgium [32] also holds a significant proportion of the known diversity. However, while regions, such as Papua New Guinea, have been relatively well explored, other regions remain poorly documented. This suggests that the diversity available in these collections is likely only partial. This gap in representativeness has prompted the present study undertaken in northern mainland Southeast Asia (nMSEA) with partners from these countries, to explore northern Vietnam, northern Laos and the Yunnan region of China. This area is recognised as a hotspot of diversity within the *Callimusa* and *Musa* sections, including potential wild relatives of cultivated bananas.

A team associating diverse disciplinary expertise, including diversity specialists from each country, conducted a field survey to collect wild and cultivated banana diversity in the target area. Morphotaxonomic descriptions, combined with genome characterisation using genotyping by sequencing (GBS), helped to document the diversity of the specimens collected and identify the contributions of ancestral forms to their genomes.

A previous study on the seeded wild forms collected during this survey [33] identified representatives of *M. acuminata* subsp. *burmannica*, *M. balbisiana*, and *M. itinerans*, as well as rarer species from the *Musa* section such as *M. yunnanensis.* Species from the *Callimusa* section, primarily known for their ornamental inflorescence, were also identified. The key finding was that, although these *Musa* species are wild, they are all affected to varying extents by human use, ranging from opportunistic gathering to full domestication. *Musa yunnanensis* and *M. acuminata* subsp. *burmannica* are the most strictly wild forms. Although they are traditionally gathered as fodder, they have not been widely dispersed by people beyond their endemic areas. *Musa itinerans* is not cultivated as such, but natural populations are widely exploited, with humans sometimes transferring the forms they consider most attractive, resulting in geographically structured diversity. *Musa balbisiana* has spread far beyond its region of origin, with a diversity geographically structured by human activities. This species should be regarded as domesticated. Northern MSEA, in the native area of *M. balbisiana*, associates endemic wild populations as well as exogen populations [33].

Within this background of crop wild relatives already shaped by human intervention, the present study focuses on cultivated bananas in our nMSEA region: northern Vietnam, northern Laos and southern China. The objective is to describe the diversity of the cultivated forms collected in the region, by comparison to the diversity previously documented. The aims are then to deduce how these bananas evolved from their ancestral genome and how they contribute to a broader history regarding the diversification and domestication of cultivated species.

## Materials and methods

### Plant material field collection

**Fig 1:**
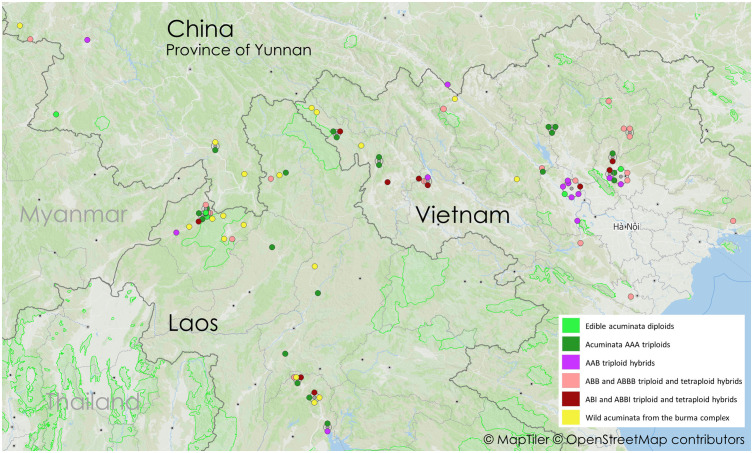
Geographical distribution of the 91 collected specimens. Points are colour-coded based on genetic composition (see legend). Locations of the collected wild burmannica specimens added for comparison. Overlapping points were offset using QGIS internal displacement option. Topographic data obtained from OpenStreetMap. Map uses WGS84 projection system.

Field surveys were conducted in two distinct phases. The first phase, undertaken in 2018, spanned northern Vietnam, extending from Ha Long Bay in the east to the northwestern borders with Laos and China. The second phase, conducted in 2019, focused on Laos, starting from Vientiane and progressing northward to the borders of Vietnam and Myanmar, followed by a land expedition into China’s Yunnan Province (Fig 1).

In order to characterize the diversity of cultivated banana varieties in the surveyed regions, any material of interest observed whether in the forest, along roadsides, in agricultural fields, or in home gardens, was collected. A systematic sampling of all encountered cultivated bananas would have disproportionately represented widely cultivated groups, unnecessarily complicating field protocols and increasing analytical costs without generating additional insights. Therefore, the research prioritized less frequent and potentially region-specific banana varieties over widespread types, except when the latter were found to have distinctive characteristics such as morphological variations or pests or diseases symptoms. Therefore, occurrence counts for each variety should be seen as indicators and should not be overinterpreted. This survey protocol is well suited to capturing the diversity of forms present in this northern part of the MSEA, but less well suited for statements about overall importance and relative abundance of a specific form.

For each site, the team recorded geolocation and administrative reference, vernacular names when available, a proposed taxonomic identification, and notes of collectors regarding morphological peculiarities and discovery contexts. Additionally, the team produced a series of photographs documenting the environment, the whole plant and its various parts. The study does not concern human subjects requiring approval from an ethics committee. The cultivated plants under study, obviously, depend on a farmer; however, no information has been collected regarding the person’s identity or any potential categorisation of any kind. The only information that could potentially be used to trace the farmer back was the geographical location. For anonymisation, the GPS coordinates have therefore been downgraded to prevent potential identification as the grower of a specific plot.

A portion of leaf lamina (approximately 10 cm²) was prepared for genomic analysis (as in [33]). While all three countries surveyed are signatories to the Nagoya Protocol, each maintains distinct regulatory frameworks governing access to genetic resources and their associated traditional knowledge. Prior compliance with country-specific procedures enabled the collection of desiccated foliar specimens, their subsequent transfer to French laboratories for genomic analyses, and their comprehensive investigation by the various research consortium members (S1 File for Material Transfer Agreements).

### Genotyping by sequencing

The genomic characterisation of collected and reference specimens was performed using a high-throughput genotyping method, specifically resequencing targeted genome regions via the GBS (Genotyping By Sequencing) technique [34]. DNA extraction followed a MATAB protocol [35]. Genotyping was conducted according to established procedures [34]. GBS libraries based on StdGBS methods were constructed following standard protocols, with PstI and MseI restriction enzymes. The 300–500 bp short-insert libraries were sequenced in 150 bp paired-end reads using Illumina HiSeq2500 (Illumina, San Diego, CA, USA) by Genewiz, Azenta Life Sciences, USA.

A first GBS analysis (batch 1) included the 182 samples collected in Vietnam in 2018, along with 34 wild and cultivated specimens from the Biological Resources Centre for Tropical Plants (CRB-PT), the field collection of CIRAD (Centre de Coopération Internationale en Recherches Agronomiques pour le Développement) in Guadeloupe, serving as a reference for global genetic diversity in the section *Musa*. A second GBS analysis (batch 2) was carried out on the 153 samples collected during the 2019 survey in Laos and China. This batch included 32 references (27 identical to batch 1) and seven previous samples rejected from batch 1 for insufficient DNA quality. In each batch, all samples were analysed twice to increase sequencing depth. Five samples were repeated on each plate as internal repeatability controls.

### Read processing and SNP calling

After demultiplexing with GBSX [36], the paired-end sequencing files (fastq_R1 and fastq_R2) were assessed for quality metrics, adapter presence, and GC content using FASTQC software [37], available at https://www.bioinformatics.babraham.ac.uk/projects/fastqc. The files were then cleaned to remove Illumina adapter sequences and low-quality ends (Phred score > 30) using Cutadapt [38]. Reads shorter than 30 bp after trimming were discarded. The cleaned reads were aligned against the v2 genome of Pahang-HD, a doubled haploid from *M. acuminata* subsp. *malaccensis* ‘Pahang’ [24], obtained from the Banana Genome Hub using BWA-MEM [39]. Re-alignment was performed with the IndelRealigner module from GATK v4.1 [40]. The GATK pipeline for non-model organisms was followed, including a recalibration step. This involved an initial round of SNP calling on the original uncalibrated data, selecting the SNPs with the highest confidence, followed by a round of base recalibration on the original mapped read files. For duplicate samples, the recalibrated BAM alignment files were merged via a script using Sambamba software. SNP and indel calling were conducted using the GATK module HaplotypeCaller v4.1. A script (gVCF2vcf -gz.pl) was written to merge the individual gVCF files into a single VCF file. The GenomicDB procedure from GATK was used to build the gVCF SNP database, and the GenotypeGVCF procedure was used to extract variant positions. The snpcluster exclusion procedure was applied to process SNP clusters, with a threshold of three or more SNPs per 10 bp window. These procedures are compiled in the pipeline Musa_NGS_Suite available at https://github.com/CathyBreton/Genomic_Evolution [41].

### Data analysis

Specimen datasets from Vietnam and Laos/China were processed independently using Musa_NGS_Suite, as they originated from separate analytical batches, resulting in two distinct raw VCF files. For each batch, a filtered VCF file was generated using GBS-Div software [33], retaining only bi-allelic loci with read depths ranging from 20 to 300. Alleles were retained only if they accounted for at least 5% of the total read depth at a given locus.

Subsequently, the filtered files were merged into a single VCF file, conserving exclusively SNPs that were polymorphic across both batches. The two surveys, conducted in neighbouring geographical areas, each included representatives from the same wide range of *Musa* species. Furthermore, 27 external references, covering a large part of the cultivar diversity, were present in both batches. Given this high degree of overlap between the two batches, it was expected that a polymorphic locus in one batch will also be polymorphic in the other. Otherwise, a bias linked to conditions specific to the analysis batches could be suspected, so we opted not to consider these potentially problematic sites. This choice necessarily resulted in a reduction in the number of SNPs selected, but the number remained high enough for diversity analyses and chromosome paintings.

The consolidated VCF file was further analysed using GBS-Div software. Statistical parameters including missing data proportions, allelic frequencies, and depth distribution patterns were assessed to detect potential outliers, which were recoded as missing data to ensure analytical robustness.

Field prospections across the three surveyed countries had resulted in the collection of a large number of samples of wild species within the *Musa* and *Callimusa* sections, as well as cultivated forms. A subset of 91 representatives was selected for this study, which focused on local cultivated bananas. This subset included the 72 diploid and polyploid collected cultivated types, as well as the 19 collected *M. acuminata* subsp. *burmannica,* included to evaluate the contribution of local wild *M. acuminata* to cultivated types (Fig 1 for their geographical distribution).

The dataset was supplemented with 26 external references, comprising 9 representatives of other *M. acuminata* subspecies, 3 cultivated AA diploids, and 14 cultivated AAA, AAB and ABB triploids provided by the CRB-PT. Except for one AAB specimen added to the second batch, all references were analysed using the same DNA samples across both batches. These technical replicates were maintained as separate entries rather than merged, serving as internal controls to assess the reproducibility of genotyping.

The genotypic data for the 91 collected specimens and 51 external references (2 × 25 + 1) (S1 Table) were extracted from the comprehensive VCF file. The resulting VCF file containing data for all 142 specimens is available through the Gigwa application [42].

For each heterozygous SNP within a specimen, a ploidy ratio was estimated by calculating the proportion of reads exhibiting the most frequent allele relative to the total number of reads for all represented alleles. The distribution of these ratios is expected to display a theoretical peak at 0.5 for diploid, 0.67 for triploid, and 0.75 for tetraploid populations. The position of the observed ploidy ratios, relative to these theoretical values, was used to determine the ploidy status of each specimen analysed [43].

Pairwise specimen dissimilarities were computed as the average, across the valid SNPs for both specimens, of the number of differing alleles relative to the number of alleles compared, i.e., the ploidy. Genetic relationships among specimens were visualized using a Neighbor-Joining tree [44], chosen for its independence from evolutionary model assumptions, that are frequently violated in clonally propagated plants subject to anthropogenic selective dispersal of seeds and suckers. Branch lengths were refined through *a posteriori* least-squares optimization.

Bootstrap analysis is usually used for assessing node support. However, its relevance in phylogeny reconstruction has been extensively debated [45]. In our case, due to the very high numbers of markers, several hundred thousand, and the same very small number of possible modalities for each marker, the random pseudoreplicates remain highly similar to the original dataset. The bootstrap values are therefore significantly overestimated and are no longer informative.

Alternative reliability estimations were therefore implemented. The tree topology assumes the absence of horizontal genetic exchange. If the tree model is locally disturbed by some events of inter-branch crossings, the observed dissimilarities between some unit subsets are lower than represented in the tree. A proposed solution [46] is to improve the tree in adding shortcuts between tree nodes, the reticulations, which significantly improved the fit between tree distances and observed dissimilarities.

All computational analyses were conducted using DARwin software [47].

### Chromosome painting

*In silico* chromosome painting was performed using GBS-Div software. Reference genomic profiles for putative ancestral pools (wild species or subspecies) were defined as the majority consensus derived from specimens considered reliable representatives of the pool. Discriminatory SNPs between these pools were identified as loci fixed for one allele in a given pool consensus while fixed for the alternate allele across all other pool consensuses.

Subsequently, genotypes at these diagnostic loci were used to generate chromosome paintings for all cultivated specimens, enabling the detection of potential contributions from these major ancestral pools to cultivated diversity. The limited number of meiotic recombination events in the evolutionary history of cultivated banana has resulted in large segments within inter(sub)specific genomic mosaics, facilitating reliable detection through chromosome painting methods using SNPs obtained via GBS. For comparison, SNPs data for additional samples available from published sources [10] were processed identically.

Seven primary pools were defined as potential progenitors, from samples collected or added as reference, leveraging the flexibility of GBS-Div which allows constructing pools from the data itself (S2 Table). Thus, pool consensuses were constructed for *M. balbisiana*, *M. itinerans*, *M. yunnanensis*, and *M. acuminata* subsp. *burmannica*, based on 101, 27, 20, and 18 collected specimens respectively, excluding specimens in marginal positions in diversity trees or those with low SNP coverage. The identity threshold was set at 80%, indicating that a genotype position is validated for the consensus if present in more than 80% of the specimens. Additional pools were established from the reference samples for other *M. acuminata* subspecies: banksii, zebrina, and malaccensis. For each, the two replicates were merged using a 100% identity threshold. Although *M. yunnanensis* exhibits spontaneous hybridization with *M. acuminata* [33], it showed no detectable contribution to cultivated forms and was not kept for the presentation of the results.

Chromosome paintings of all cultivated specimens were then generated, showing the contribution of these primary pools to their genome. Genomic segments without assigned ancestry (shown in gray and labelled ‘N.D.’ for ‘not defined’) represent unidentified ancestral sources. They most often represent only a small fraction of the genome of cultivated forms and do not substantially alter conclusions based on major pools considered here.

The chromosome lengths displayed in the paintings are scaled to the position of the last SNP in the v2 DH Pahang genome [48]. These lengths are only indicative, as recent haplotype-resolved genome assemblies (*M. acuminata* subsp. *malaccensis* [49]; AAA Cavendish [50]; AAB Plantain and Silk [51]) demonstrate significant variation in chromosome lengths between species or subspecies. Similarly, the centromere positions are estimated from the v2 reference genome, and these above publications highlight variability in their locations across genomes. Despite these uncertainties in representation, the large non-recombined segments of the mosaic remain highly informative for identifying parental origins.

Both DARwin and GBS-Div software are freely available at: https://agap.gitlab.cirad.fr/diversity-tools/

## Results

An initial VCF file, in which the two replicates of each specimen were merged, was generated separately for each batch. Using GBS-Div software, an intra-batch dissimilarity was calculated between all pairs of specimens, confirming the very high similarity between the five replicate samples serving as internal repeatability controls. Next, to verify consistency between the two batches, the 27 reference samples analysed jointly across both batches were subjected to a joint factor analysis (data not shown). The near-perfect match on the first factorial plans between the two points representing the same specimen confirmed the consistency of the two batches. The specimens from each batch, after filtering, were then merged in a single VCF file, conserving SNPs polymorphic across both batches.

Analysis with GBS-Div the merged file for the 142 selected samples (91 collected specimens and 51 external references) showed an average of 195,270 valid SNPs per sample. Locus depths ranged from 34 to 78, with a mean depth of 49.7. These high depths were achieved by merging the two sequencing replicates of each sample, enabling reliable ploidy and genotype estimations. Detailed metrics, including valid SNP counts, mean depths, heterozygosity (calculated as the ratio of SNPs with two alleles to the number of valid SNPs for the specimen), and ploidy ratios for the 142 analysed specimens, are presented in S1 Table.

The passport data attached to the reference specimens from the CRB-PT in Guadeloupe included an estimation of the ploidy determined by cytometry. These accessions have also been transferred to the International Transit Center (ITC), in Belgium, where the ploidy was also measured by cytometry [31]. For all these specimens, the cytometry measures confirmed the ploidies inferred from ploidy ratio.

The ploidy levels proposed for collected specimens from morphological observations were mostly confirmed, with only a few atypical specimens, initially designated as diploid by default, proving to be triploid. As detailed below, five specimens were proposed to be tetraploid. Based on these ploidy estimations, a pairwise dissimilarity matrix was computed across all 142 specimens, using the ploidy-dependent index proposed in GBS-Div. On average over all pairs of specimens, 106,000 loci were available for the dissimilarity measure, with almost 90% of the pairs having between 80,000 and 170,000 SNPs in common.

As the dissimilarity measure depends on the ploidy of the specimens, the observed dissimilarities are comparable only for specimens with identical ploidy levels. Two separate unrooted NJ trees were therefore produced for diploid and triploid forms, the specimens shown to be possibly tetraploid were too few in number to construct a specific tree.

**Fig 2:**
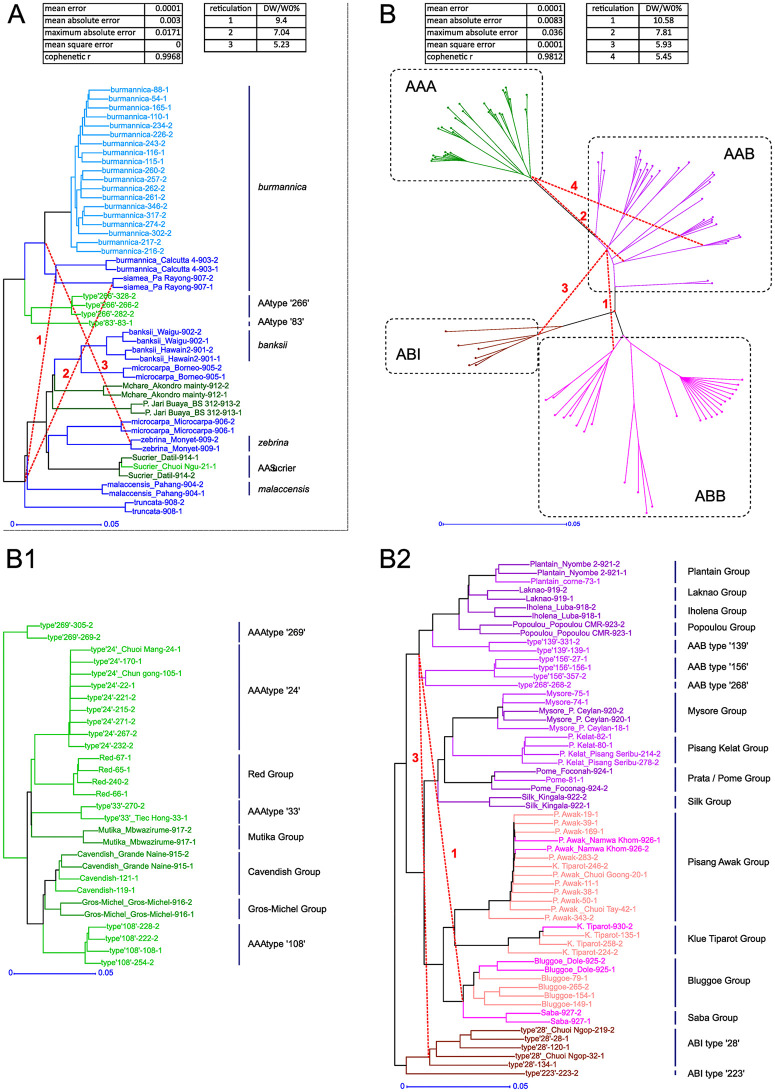
Genetic dissimilarity analysis. (A): Neighbour-Joining tree of the 48 diploids specimens including wild *M. acuminata* and cultivated AA diploids, *a posteriori* rooted with *M. balbisiana* as outgroup (not displayed). Dark and light blue indicate reference and collected *M. acuminata* specimens, respectively, while dark and light green represent reference and collected cultivated AA. (B): unrooted tree of the 89 triploid specimens. (B1): partial subtree of the 29 AAA triploids rooted as in B (collected specimens in light green, reference specimens in dark green). (B2): partial subtree of the 60 interspecific triploids rooted as in B, including AAB specimens (collected in light violet, reference in dark violet), ABB specimens (collected in light purple, reference in dark purple), and ABI specimens (in brown). Red dashed lines indicate major reticulation events inferred in both diploid and triploid trees.

The tree for diploids (Fig 2A) clearly isolated the cluster of locally collected burmannica specimens, associated with the burmannica references Calcutta 4 and Pa Rayong. Four cultivated AA specimens were linked to this cluster. The other part of the tree grouped the remaining *M. acuminata* subspecies and the AA cultivars added as references, along with a small group of specimens including a locally collected AA specimen and the reference for the AA Sucrier Group. The two strongest reticulations attempted to connect the reference group for the burmannica subspecies (Calcutta 4 and Pa Rayong) with the cluster of other subspecies of *M. acuminata*, suggesting these forms might not be purely burmannica [13].

The tree for triploids (Fig 2B) clearly distinguished between the AAA and ABB clusters, and positioned between them, several AAB groups and a particular cluster that will be recognized by the genome analysis as an ABI cluster (‘I’ referring to *M. itinerans*). This highly unexpected composition will be presented in greater detail below. The hierarchical representation of the AAA specimens only (Fig 2B1) showed that six of the collected samples belonged to Red and Cavendish Groups. However, surprisingly, the other collected AAA varieties were distinct and not genomically similar to any of the references included in our sampling. They were not morphologically related to any known AAA Group either.

For the interspecific forms (Fig 2B2), all collected ABB specimens aligned both morphologically and genotypically with the groups represented in the external references. Four of the specimens belonged to Bluggoe, twelve to Pisang Awak, and three to the Klue Tiparot Groups. Among the AAB specimens collected, several were related to known Groups represented in the external references. Four were Pisang Seribu of the Pisang Kelat Group, three were Pisang Ceylan of the Mysore Group, one was of the Pome Group, and another of the Plantain Group. However, the other six AAB specimens were not related genomically to any of the reference groups and could not be morphologically linked to any known types; they were original and specific to the region. In the diversity tree, these six specimens were in the AAB branch grouping Plantain, Iholena, Popoulou, and Laknao Groups, from the eastern banana distribution area: the Philippines, New Guinea, Oceania. Interestingly, the first reticulation in the diversity tree indicated an additional affinity between this set of AAB specimens and the Bluggoe ABB Group, which is also well distributed throughout the region.

**Fig 3:**
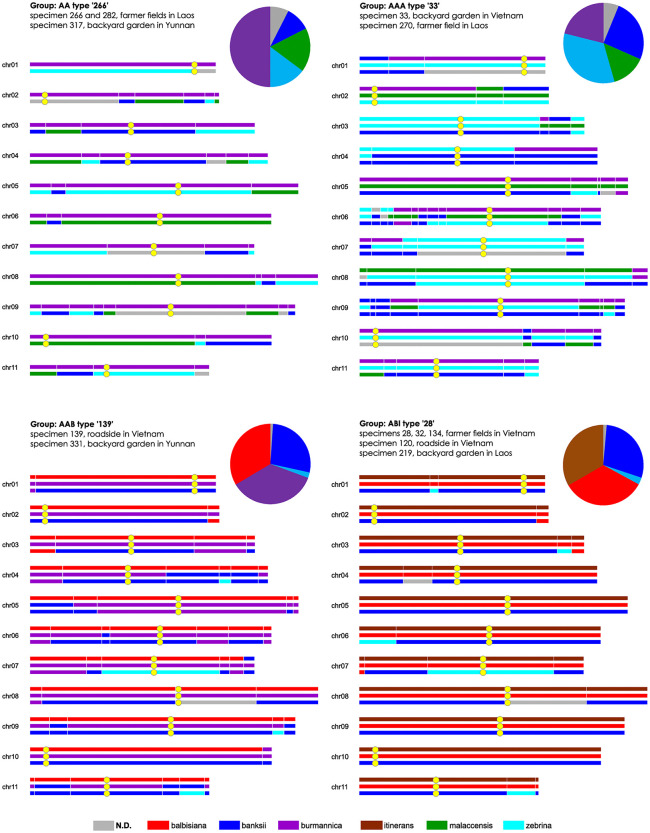
Examples of genomic ancestry profiles. Examples of AA, AAA, AAB, and ABI types showing mosaics of ancestral pools for each of the 11 chromosomes. A pie chart summarises the contribution of each ancestral pool to the complete genome. The list of specimens belonging to the profile is also provided. For an overview of the 38 distinct genomic profiles observed, refer to S2 File, ‘Genomic ancestry profiles.

Chromosome painting genomic profiles were generated for all specimens. Specimens with strictly identical profiles were grouped into a profile type, and those with almost identical profiles were defined as variants of this type. All observed profile types and their potential variants are shown in the supplementary file ‘genomic ancestry profiles’ (S2 File; see also Fig 3 for examples of these genomic profiles at different ploidy levels, and Table 1 listing the different genomic profiles identified). Specimens that share a given profile are indicated, along with their environment of collect. A pie chart summarises the relative contributions of each ancestral pool across all 11 chromosomes. Incidentally, the three published haplotypic sequences mentioned above were compared to the genomic profiles obtained here for the same varieties. Their similarity demonstrates that the parsimony principle used to infer the phasing of genomic segment mosaics is largely valid, with only a few exceptions as expected from a probabilistic principle.

**Table 1:** Distribution by country of the 38 genomic ancestry profiles. Specimens (ID VCF) by country over the 38 genomic ancestry profiles described in S2 File.

Genomic profiles^a^	Genomic comp.	Group/ Type	Ploidy	Specimen (ID VCF) from			
				Vietnam	Laos	China	Ref.
1	AA	Sucrier	2	21			914
2	AA	type ‘83’	2	83			
3	AA	type ‘266’	2		266 282	328	
4	AAA	Cavendish	3	119 121			915
5	AAA	Red	3	65 66 67	240		
6	AAA	type ‘24’ – Orotava	3	22 24 105 170	215 221 232 267 271		
7	AAA	type ‘269’	3		269	305	
8	AAA	type ‘108’	3	108	222 228 254		
9	AAA	type ‘33’	3		33 270		
10	AAB	Plantain	3	73			921
11	AAB	Pome	3	81			924
12	AAB	Mysore	3	18 74 75			920
13	AAB	P. Kelat_Pisang Seribu	3	80 82	214 278		
14	AAB	type ‘139’	3	139		331	
15	AAB	type ‘156’	3	27 156		357	
16	AAB	type ‘268’	3		268		
17	ABB	P. Awak	3	11 19 20 38 39 42 50 169	246 283		926
18	ABB	P. Awak variant ‘343’	3			343	
19	ABBB	P. Awak 4x	4	91			
20	ABB	Bluggoe	3				925
21	ABB	Bluggoe variant ‘79’	3	79			
22	ABB	Bluggoe variant ‘149’	3	149	265		
23	ABB	Bluggoe variant ‘154’	3	154			
24	ABB	K. Tiparot	3	135	258		930
25	ABB	K. Tiparot variant ‘224’	3		224		
26	ABI	type ‘28’	3	28 32 120	219		
27	ABI	type ‘28’ variant ‘134’	3	134			
28	ABI	type ‘223’	3		223		
29	ABBI	type ‘77’	4	77			
30	ABBI	type ‘77’ variant ‘132’	4	132 138	272		
31	AA	Mchare Akondro mainty	2				912
32	AAA	Gros-Michel	3				916
33	AAA	Mutika	3				917
34	AAB	Iholena	3				918
35	AAB	Laknao	3				919
36	AAB	Silk	3				922
37	AAB	Popoulou	3				923
38	ABB	Saba	3				927

^a^Profile number in S2 File.

A significant diversity of cultivated varieties, both diploid and, even more so, triploid, was then highlighted. Initial identification in the field was based on morphological characteristics, drawing on the experience and knowledge of experts in *Musa* diversity. Analysis of genomic profiles, establishing construction patterns from ancestral pools, has enabled refinement or modification of this classification. The presentation below of this diversity, according to major diploid or triploid groups, confirms that several specimens belong to previously known groups. However, numerous new types have emerged that, to our knowledge, have never been described or referenced before.

### Cultivated AA diploids

The cultivated parthenocarpic diploid bananas collected in the region are few, considering what might be expected in this area of diversification. One specimen collected in Vietnam, named ‘Chuoi Ngu’, links directly in the phylogenetic tree to ‘Datil’, the reference of the widely cultivated AA Sucrier Group (Fig 2A). A second similar specimen was observed in Laos (but not analysed due to lack of reliable data). The genomic organisation of ‘Chuoi Ngu’ was identical to the reference ‘Datil’, it showed a predominant contribution from the banksii subspecies (S2 File, page 1) as well as zebrina and, to a lesser extent, malaccensis.

**Fig 4:**
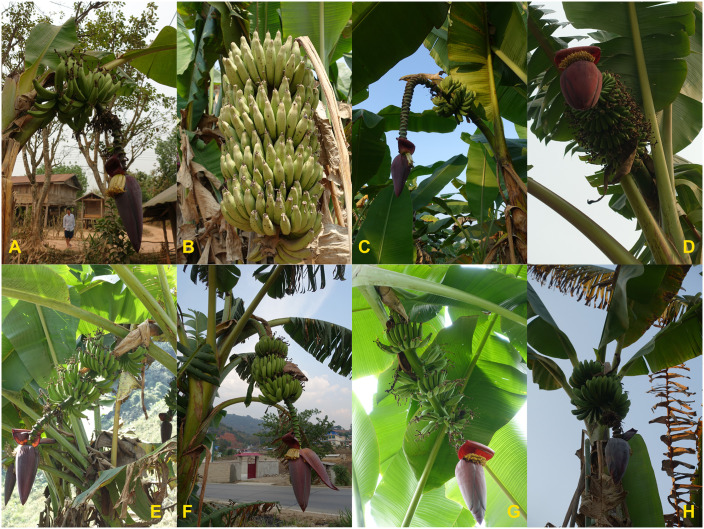
Morphological diversity among collected AA, AAA, and AAB specimens. (A-B): AAcv Kouay/ Lady Finger, type ‘266’; (C): AAcv type ‘83’; (D): AAA type ‘269’; (E): AAA type ‘108’; (F): AAB type ‘139’; (G): AAB type ‘156’; (H): AAB type ‘268’. - Pictures © Matthieu Chabannes – CIRAD.

A type that was not morphologically related to any other known AA types has been designated here as type ‘266’ (Fig 4A and Fig 4B). This type was represented by three specimens that were collected in home gardens: one in Yunnan and, over 200 km away, two closely located in Laos. This type ‘266’ was different but genetically related to another unknown type, designated here as type ‘83’, which was collected as a single specimen in Vietnam. These types, grown only locally, can be considered landraces, as opposed to the cultivar Sucrier, for example, which is widely grown in all banana-producing regions in the world. Both types displayed a complete *M. acuminata* subsp. *burmannica* haplotype (S2 File, pages 2–3), paired with complementary haplotypes that were not strictly identical for the two types. These complementary haplotypes, which involved banksii, zebrina and malaccensis in similar proportions in both cases (S2 File, pie charts on pages 2 and 3), showed strong similarities for several chromosomes but differed at least partially for others, suggesting two independent crossing events of *M. acuminata* subsp*. burmannica*, either with two different gametes from the same parental AA form, or with gametes from two different but closely related AA forms.

However, morphologically, these two types were strikingly different from each other. Type ‘83’ (Fig 4C) presented a horizontal bunch of small fruits, followed by a rachis that curves at a right angle downwards. It terminated in a purple male bud, whose shape and colour are found in the burmannica complex [33]. The last bract rolled upwards, revealing the internal discolouration characteristic of *M. acuminata*. With fruits that were barely larger than those of wild forms, type ‘83’ was morphologically very reminiscent of the burmannica complex specimens, from which it inherited half its genome. In contrast, type ‘266’ (Fig 4A and Fig 4B) exhibited characteristics of an edible acuminata diploid cultivar, with a sub-horizontal to vertical bunch that could be quite impressive, with numerous compact hands consisting of long fingers with pronounced apex. While the colour of the bud bracts was reminiscent of the burmannica complex, the internal discolouration of the bract appeared less pronounced, or even absent in some cases.

### Cultivated polyploids

Representatives of the main triploid groups cultivated worldwide were found among the specimens collected, but several types not yet referenced were also observed in our nMSAE zone.

#### Cultivated AAA.

Thus, regarding purely acuminata triploids, the classic AAA Cavendish Group was frequently encountered in intensive cultivation across all three countries (‘Chuoi Tieu’, or ‘Chuoi Tieu Lun’ for the dwarf form, in Vietnam, ‘Kuay Ngao’ in Laos, ‘Xiangjiao’ in Yunnan). Only one specimen of this type (119, and its sucker 121) was collected in Vietnam for genomic analysis. The genomic mosaic (S2 File, page 4) was identical to that of the Grande Naine reference (915). In addition to segments from the banksii, zebrina and malaccensis subspecies, a significant number of indeterminate segments were observed. They corresponded partly to unrepresented ancestral pools, and partly to uncertainties arising from the similarity between purely acuminata ancestral genomes as well as the scarcity of diagnostic loci in certain genome areas, particularly in centromeric regions.

Four specimens were identified as belonging to the Red Group. Although this type was not represented in our reference data, the strong morphological specificities of this group ensured identification. Furthermore, the genomic profile common to the four specimens (S2 File, page 5) showed a close resemblance to that of a recently published Red-type accession [52]. The three specimens from Vietnam all originated from the same location (65 and 66, one spontaneous by the roadside, the other in a garden, and 67 was a green variant among the red ones) while one specimen (240) was found in northern Laos. The distribution of contributions from the various subspecies of *M. acuminata* was similar to that of AAA Cavendish, indicating common construction patterns, probably from the same AAcv population and in the same geographical region.

In addition, several AAA landraces were also collected, their AAA genomes could not be assigned to any of the AAA references in the dataset (Fig 2B1).

Type ‘24’, with nine specimens, was the most represented. Four specimens came from Vietnam: two geographically close (22 and 24, under the name ‘Chuoi Mang’) from Thai Nguyen province, one further west (170), and the specimen (105), said ‘Chuoi Goong’, at the Laotian border. The other five specimens spread from southern Laos in the Vientiane region (215, 221), to central Laos (232) and to the north, up to the Chinese border (267, 271). The genomic mosaics of all these specimens were identical (S2 File, page 6), with contributions from banksii, zebrina and malaccensis, in proportions similar to those of AAA Cavendish and AAA Red, indicating a similar construction pattern. The genomic structure did not match any of our AAA references in the dataset but strongly resembled that of the ‘Hom Sakhon Nakhon’ specimen from the CRB-PT collection in Guadeloupe and originating from northern Thailand, near the Laotian border [52]. This supported the assignment of type ‘24’ to the AAA Orotava Group, the group of ‘Hom Sakhon Nakhon’. This group is morphologically similar to Cavendish and occupies the same cultivation niche.

Two specimens in northern Laos (269) and southern Yunnan (305), classified as type ‘269’, shared distinctive morphological characteristics with an almost horizontal bunch and fruits that remain small, which initially led to field identification as diploid AA. In the phylogenetic tree, they form the most distant group from all others. Their genomic structure (S2 File, page 7) also indicated a triple contribution from banksii, zebrina and malaccensis subspecies. However, the malaccensis contribution was very strong, as it encompassed complete haplotypes for several chromosomes. One haplotype was almost entirely malaccensis across all chromosomes, while a second haplotype was predominantly malaccensis but locally recombined on at least eight chromosomes. This genomic structure is rare and likely originates from a region further south, within the distribution range of the malaccensis subspecies. Morphologically, the distinctive blood-red colour of the male bud is an unambiguous trait of malaccensis origin (Fig 4D).

The AAA type ‘108’, represented by four specimens, clustered in the tree with the Cavendish and Gros-Michel Groups. However, it differed significantly from them as their genome (S2 File, page 8) incorporated a complete burmannica haplotype. To our knowledge, this configuration has never been described before. The elongated shape and violet colour of the male bud were consistent with this genomic composition (Fig 4E). Three specimens were collected in central Laos and one in Vietnam, at the Laotian border. The wide geographical distribution within the Laotian zone indicates a locally popular variety.

Finally, the AAA type ‘33’ incorporated, like type ‘108’, a burmannica contribution in one of its haplotypes, but here it was extensively recombined across most chromosomes (S2 File, page 9). It was present in Vietnam (33), collected under the name ‘Tieu Hong’, and in northern Laos (270), nearly 500 km further west, thus also indicating a wide geographical distribution. For most chromosomes, similarities were found between the two non-burmannica haplotypes and the local AA ‘Chuoi Ngu’ (Sucrier) suggesting a kinship. In contrast, similarities with local AA displaying a burmannica contribution, such as types ‘83’ and ‘266’, were very limited.

#### Cultivated AAB.

A wide diversity was also observed among interspecific triploids. Here again, previously undescribed types were identified. Among AAB triploids, the types commonly cultivated elsewhere were poorly represented. The few specimens belonging to known groups – two AAB Mysore, two Pisang Kelat and one from the Pome group – were collected from the NOMAFSI collection at Phu Ho in northern Vietnam. This collection conserves specimens of various origins, and there is no indication that these specimens are of local origin; they have been considered here as references complementing the initial references in the dataset.

One specimen (18) identified as AAB Pisang Ceylan, an emblematic cultivar of the Mysore group, was collected in eastern Vietnam. Its genome organisation (S2 File, page 12) was identical to that of the Pisang Ceylan reference (920) and the two AAB Mysore specimens from the Phu Ho collection (74, 75). The balbisiana haplotype shows that segments of chromosomes have been exchanged with the acuminata genome, mainly in the distal regions of chromosomes 3, 4, 9 and 11. This suggests that mitotic recombination [53] may have occurred at these distal positions after zygote formation during the course of somatic cell divisions. A second haplotype is predominantly zebrina, nearly complete for 8 of the 11 chromosomes. The third haplotype shows contributions from malaccensis, banksii and zebrina subspecies. This type, widespread in SE Asia, is considered to be native from India.

Two specimens (214, 278) were identified as Pisang Seribu by their highly specific morphological traits, particularly the bunch structure (‘seribu’ means 1000 in Thai; in Vietnam, it is ‘Chuoi Tram Nai’, with ‘tram’ for 100 and ‘nai’ for hands, similar to ‘thousand fingers’ in English). Both specimens originated from Laos: 214 from Vientiane Province in the south and 278 from Luang Namtha Province in the far north, near the border with Yunnan, where they are commonly planted as ornamentals. The two specimens had identical genomic structures (S2 File, page 13) and were also identical to the P. Kelat specimens from the Phu Ho collection with which they clustered in the diversity tree. Genomically, one haplotype was purely balbisiana, the second almost purely zebrina, and the third was also predominantly zebrina, but with banksii introgressions and very occasional malaccensis contributions. Pisang Seribu appears to be a morphological variant of P. Kelat, with a variation having a particularly striking phenotypic effect that has justified its preservation by farmers and its wide distribution throughout SE Asia. Its presence in Laos is therefore probably not a marker of geographical origin, an origin that the banksii/zebrina genomic structure would place further south, like P. Kelat, a variety that was never found in farmers’ plots in this study.

Only one specimen of the plantain type was observed, located a short distance north of the Phu Ho collection in Vietnam. It is a Horn type, the most commonly cultivated type in SE Asia, often called Tandok/Tanduk/Tindok (meaning ‘Horn’). Genetically related groups to plantain such as Laknao, Iholena or Popoulou are not represented. The Indian AAB Silk group is also absent.

However, several landraces that could not be linked, either morphologically or genetically, to known groups, were collected and noted here as types ‘139’, ‘156’, and ‘268’.

The two specimens of type ‘139’ originated from different locations: one (139) from the Dien Bien Phu region in Vietnam, and the other (331) much further north, in Yunnan, near the Burmese border, at about 800m altitude (Fig 4F). Similar plants were observed in Laos but were not collected. The genome structure showed an interspecific AAB hybrid (S2 File, page 14). The balbisiana haplotype was complete. Two exchanges occurred with the acuminata genome at the ends of the chromosomes 7 and 10 which again could be due to mitotic recombination. The second haplotype was of burmannica type; it was complete for 6 chromosomes, with banksii insertions for the others. The third haplotype was predominantly banksii with some zebrina insertions but no trace of malaccensis contribution.

Type ‘156’ was represented by three specimens: two, geographically quite distant, in central Vietnam (27, 156), and the third (357) further north, in Yunnan, just at the Vietnamese border, at low altitude (Fig 4G). The genome structure also showed an AAB genome (S2 File, page 15) with a complete balbisiana haplotype (without segment exchange with the acuminata genome), a burmannica haplotype complete for 7 of the chromosomes and hybridised with other *M. acuminata* subspecies for the remaining chromosomes. The third haplotype was acuminata with a large malaccensis contribution.

Type ‘268’ was represented by a single specimen from northern Laos, near the Chinese border, at around 800 m altitude (Fig 4H). The genomic structure (S2 File, page 16) was similar to that of type ‘156’, with a pure balbisiana haplotype, a second haplotype that was pure burmannica for at least five chromosomes but recombined for the others, and a third acuminata haplotype combining several subspecies, but in somewhat different proportions.

Morphological characteristics common to AAB types were observed for these three unidentified types (Fig 4F to Fig 4H), but they did not exhibit the specific assemblages observed in known groups today. Light blotches at the basis of the petiole, narrow petiolar canal, absence of internal discolouration of the bract – which re-rolls after opening – and male flowers of predominantly cream colour, were among these characteristics. Only type ‘139’ (Fig 4F) had orange male flowers and bracts that were more reddish than purple. In all three types, bunches are followed by a bare rachis. The orientation of the bunch varies from oblique to vertical, though it is unclear whether this is related to the weight of the bunch or a genetic feature. The medium-length fruits are curved, with marked ridges, when ripe. Variations in pseudostem colour are observed among the three types. It should be noted that the presence of a burmannica haplotype does not confer the typical characteristics of AA burmannica to these types.

#### Cultivated ABB.

In contrast to AAB types, bananas with ABB genomic composition belong to already known cultivar groups.

- *Pisang Awak*

The ABB-type Pisang Awak (P. Awak) variety was the most frequently observed banana type in this study, across all three countries: 8 specimens were found distributed in different regions of Vietnam, 2 in Laos, and 1 in China. Ten of these specimens exhibited the same genomic profile (S2 File, page 17), which we have considered as the standard profile due to its prevalence. This standard profile matched that of the Namwah Khom reference variety, originating from Thailand, confirming the morphological identification as P. Awak. This profile was of ABB type with two balbisiana haplotypes; the acuminata haplotype was derived from a complex AA hybrid involving banksii, zebrina, and predominantly malaccensis, as well as other subspecies in undetermined segments. However, for 5 chromosomes, a distal segment of the acuminata haplotype was replaced by a balbisiana sequence (4 cases) or vice versa (3 cases), resulting locally in BBB or AAB configurations.

In contrast, specimen 343 collected in Yunnan showed variations in these terminal substitutions for 7 of the chromosomes (S2 File, page 18), in a background that remained clearly P. Awak; this type can therefore be considered another variant of P. Awak, originating, like the standard type, from an initial P. Awak. It differed also from another variant recently identified [52].

The P. Awak variety is widely distributed and highly valued for its sweet fruit, which can be consumed raw when ripe. It is extensively cultivated, particularly in MSEA where it is a very popular variety, indicating a possible origin in this region, probably in Thailand where numerous morphologically diverse types are cultivated [54]

**Fig 5:**
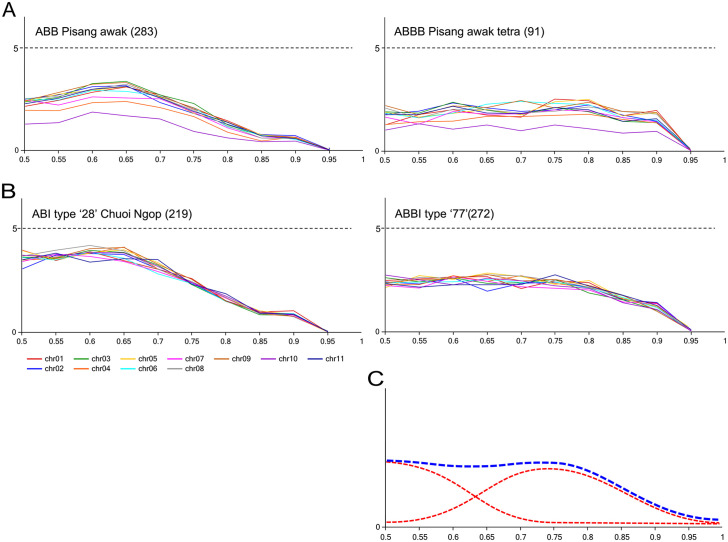
Distribution by chromosome of ploidy ratios. Expected ratios: 0.5 for heterozygous diploid loci, 0.66 for triploid loci, and 0.75 for tetraploid loci. (A): ABB Pisang Awak genotype and ABBB tetraploid variant. (B): ABI genotype and ABBI tetraploid variant. (C): interpretation of the tetraploid model (blue dotted line) as a mixture of ploidy ratios around 0.5 (2:2) and 0.75 (3:1) (red dotted lines).

The specimen 91 collected in Vietnam should also be classified under the Pisang Awak type. The ploidy distributions showed very flat frequency distributions for all 11 chromosomes, ranging between 0.5 and 0.75, indicating a possible tetraploid genome (Fig 5A). This distribution corresponds to a mixture of loci with 2/2 distribution (ratios close to 0.5) and 3/1 distribution (ratios close to 0.75) (Fig 5C). The structure of this tetraploid genome (S2 File, page 19) appeared predominantly of ABBB type, with the addition of an extra balbisiana genome to the basic ABB form, which would here be of the standard type, except for chromosome 3 which presented specific distal substitutions. For segments with AAB structure in the triploid form, the addition of a balbisiana genome locally resulted in segments with a ratio 0.50/0.50, corresponding to an AABB structure.

This ABBB structure could result from the hybridisation of a P. Awak 3x gamete, produced by apomeiosis [55], and a *M. balbisiana* gamete. The high female fertility of P. Awak supports this hypothesis. Such tetraploid variants are not rare and have been demonstrated by flow cytometry and chromosome counting [56] or by sequencing [57]. The tetraploids P. Awak previously identified [19,52] differ from the variant found here as they do not present the AABB structure at the telomere of chromosome 3.

- *Bluggoe*

The ABB Bluggoe Group was represented by four specimens, three from Vietnam and one from Laos. Their profile was of ABB type with two balbisiana haplotypes and one acuminata haplotype. The acuminata haplotype showed banksii and zebrina segments but did not incorporate any malaccensis contribution. The specimens collected had three slightly different profiles: specimen 79, type ‘149’ (specimens 149 and 265), and specimen 154 (S2 File, pages 21–23). These three types diverged by the segments exchanged between acuminata and balbisiana haplotypes. The Dole reference (S2 File, page 20) presented another slightly different profile. Apart from a length variation of a banksii insertion within a balbisiana genome for chromosome 9, the observed variations all related to the extent of the balbisiana substitutions in the acuminata haplotype of chromosomes 1, 2, 4, 7 and 11. All these forms correspond to different variants of a probably unique ABB Bluggoe ancestor.

Despite the frequency of variant forms observed in the region, the composition of the acuminata haplotype does not suggest local formation. Primarily a cooking banana but also eaten as a dessert banana, the Bluggoe variety, which is particularly vigorous, is widely distributed across Asia and has spread worldwide probably from a region further east in Asia, possibly the Philippines.

- *Klue Tiparot*

Three specimens of the ABB Klue Tiparot group were also collected in the area, in Laos and Vietnam, near the border with Laos. Samples 135 and 258 have genomic profiles identical to Klue Tiparot reference (930) (S2 File, page 24). The structure was of ABB type with two pure balbisiana genomes and one acuminata genome that was very often invaded by balbisiana segments, this balbisiana portion becoming predominant for several chromosomes and even covering the centromeric region, suggesting some chromosome substitution events. For chromosomes 2, 8 and 10, the entire haplotype was balbisiana, resulting in a BBB structure for these chromosomes. The remaining acuminata segments showed banksii and burmannica contributions. Specimen 224 (S2 File, page 25) differs from the Klue Tiparot reference (930) by a better preserved acuminata haplotype, particularly by burmannica segments, for 7 of the chromosomes.

**Fig 6:**
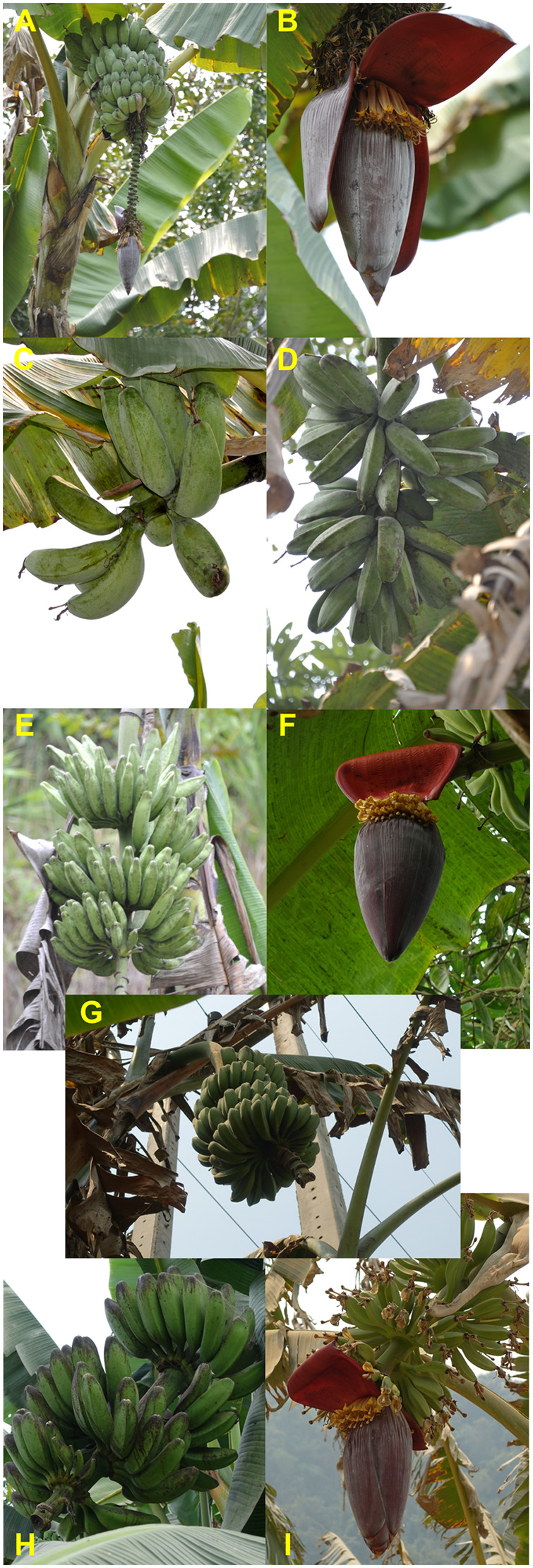
Morphological diversity among observed ABB Klue Tiparot and ABI/ABBI specimens. (A-B): ABB Klue Tiparot types ‘135’ and ‘224’ with “French” morphotype (complete bunch and male bud). (C): ABB Klue Tiparot (not sampled) with “Horn” morphotype (short bunch without male bud). (D): ABB Klue Tiparot type ’258’ with “French-Horn” morphotype (long bunch without male bud). (E-F): ABI type ’28’. (G): ABI type ‘223’. (H-I): ABBI type ‘77’. All pictures © Matthieu Chabannes – CIRAD, except (C) © Gabe Sachter-Smith.

The Klue Tiparot variety has often been described as purely balbisiana (BBB), or even tetraploid ABBB. However, it has been shown that the structure is indeed triploid and of ABB type [58], even though the genomic profiles do show significant sections of the genome as purely balbisiana, which likely explains the hesitations in morphological identification. Klue Tiparot is known for the versatility of its inflorescence, sometimes complete, sometimes truncated as in the so-called ‘Horn’ types of AAB Plantains [58,59]. Among the three specimens analysed here (Fig 6A to Fig 6D), two presented the complete bunch morphotype with male bud (135 and 224), comparable to the French type of AAB Plantains. Sample 258, although bearing a bunch of four to five hands, had an absent bud and slightly longer fruits. This morphotype had not previously been described for Klue Tiparot. It can be likened to the rare French-Horn morphotype sometimes observed in AAB Plantains. Specimens presenting the classic Horn morphotype were also noted, but not sampled, within the morphological diversity observed in the field.

#### Cultivated ABI and ABBI.

One of the major discoveries made during this study was the identification of hybrids incorporating the local species *Musa itinerans*. In the field, these specimens intrigued the botanists of the team. The ABB composition was proposed based on characteristics commonly found in varieties with two balbisiana genomes (Fig 6G): large waxy plants, showing few blotches on the pseudostem, almost closed petiolar canal and fruits with marked ridges. For other characteristics, the signature of a double B genome was less evident. Certainly, the inner face of the bract was not discoloured, but this is already the case in AAB varieties. The male flowers were just cream-orange, and not pink or red. Finally, the fruit shape, although resembling the balbisiana type, was not as typical and, in some specimens, the pedicel appeared to be uncommonly long. These individuals, classified as ABB in the field, nevertheless formed a distinct group in the diversity tree (Fig 2B2).

Genomic profiles analysis demonstrated that these specimens were hybrids with the species *M. itinerans*, with a composition that we will denote as ABI, adopting I for *M. itinerans*, by extension of the usual notation conventions.

Two different types of triploid specimens showed an ABI structure. Type ‘28’ (Fig 6E and 6F) was collected on four places, in both Vietnam and Laos, with identical genomic profiles (S2 File, page 26). Sample 134 was a variant of this type ‘28’, showing a small balbisiana substitution at the beginning of chromosome 9 (S2 File, page 27). The itinerans haplotype was complete for all 11 chromosomes, without recombination with the acuminata or balbisiana haplotypes. The balbisiana haplotype was also complete and not recombined, with small balbisiana segments substituting the ends of several acuminata chromosomes. The acuminata haplotype was predominantly of banksii origin, with introgressed segments from zebrina.

The second ABI structure, corresponding to type ‘223’, was represented by only one sample from central Laos (Fig 6G). It had an ABI structure very similar to type ‘28’, but the acuminata haplotype was very largely burmannica, with large banksii segments and some unidentifiable segments (S2 File, page 28).

Four specimens were assumed to be tetraploid based on their ploidy distributions observed along the chromosomes (Fig 5B). The spread of the distribution is explained by the significant proportion of values close to 0.5 (2/4) at specific balbisiana loci and 0.75 (3/4) at specific acuminata and itinerans loci (Fig 5C). The genomic profiles indicated ABBI genomic constitutions. The specimen 77 (S2 File, page 29) corresponded to the addition of a supernumerary B haplotype to the ABI type ‘28’ (S2 File, page 26). Three other identical specimens (132, 138 and 272) differed from type ‘77’ by a large balbisiana substitution at one end of chromosome 9 (S2 File, page 30), similar to that observed in variant ‘134’ of ABI type ‘28’ (S2 File, page 27). These three specimens therefore constitute a variant, noted ‘132’, of the ABBI type ‘77’. This variant derives from the type ABI ‘28’ variant ‘134’, in the same way that the ABBI type ‘77’ derives from the ABI type ‘128’.

Specimen 77 was collected in the northwest of Hanoi at low altitude, specimens 134 and 138 were collected east of Dien Bien Phu in Vietnam, in a mountainous area around 800 m, and specimen 272 in northern Laos near the Chinese border, at around 700 m elevation. The dispersion of these ABBI was therefore very similar to that of the ABI, from which they are not so easily distinguishable phenotypically (Fig 6H to Fig 6I).

## Discussion

The results of this study demonstrate the existence of original forms of cultivated banana in MSEA, they also provide a deeper understanding of the process of cultivated banana creation. The genomic profile established by chromosome painting for each sample provides information on its ancestral components and on the evolutionary process of its creation.

The first evidence is the very wide and unexpected diversity within the cultivated forms in this nMSEA zone. This diversity is likely even broader, since the survey in practice covered only a limited part of the area. It should be noted that the cultivated varieties are mostly sweet and eaten ripe as fruit, whether they are AAA, AAB or ABB forms. Starchy bananas are not part of the staple diet, and daily cooked starch usually comes from rice or some root crops. This contrasts with regions further east -Philippines, New Guinea island and Oceania – where cooking types are most abundant and often the basis of the diet.

Our study demonstrates that several *Musa* crop wild relatives, endemic to this northern MSEA zone, are involved in a significant number of banana landraces. For *M. acuminata*, this zone is exclusively that of the burmannica subspecies. The species *M. balbisiana* and *M. itinerans* are also spontaneously present in the zone.

### Introduced AA exogenous cultivars as a prerequisite

Despite their close sympatry, no landrace has been observed to result exclusively from hybridisation between these indigenous wild forms. Like the other *M. acuminata* subspecies, the burmannica subspecies has not been domesticated on its own, and no cultivated forms are known where the A genomes would be strictly burmannica. However, their hybridisation with diploid AA cultivars originating from outside the area and deriving from other *M. acuminata* subspecies has generated a series of diploid or polyploid landraces that are original and highly specific to the zone.

These AA cultivars, said exogenous since their ancestral forms are not spontaneous in the area, presumably exhibited the characteristics of parthenocarpy and sterility required for fruit domestication, which are *a priori* absent in local forms. These imported cultivars, hybrid forms between subspecies of *M. acuminata*, presented at least residual fertility that enabled hybridisations with local wild species to create a new diversity of cultivated bananas. The pre-domestication demonstrated for these local wild species [33] likely facilitated proximity in backyards with the introduced cultivated forms, promoting hybridisations, otherwise highly unlikely, and the retention by farmers of any resulting forms that could be of some interest.

These exogenous AA are poorly represented among the specimens collected, with Choi Ngu, from the Sucrier Group, being one of the rare examples. This cultivar in particular is surprisingly rare, since it is generally highly appreciated and cultivated in all regions of the world, for self-consumption or local markets. Nevertheless, other exogenous AA forms have probably been introduced, as shown by the diversity of the genomic profiles of their contribution to the local triploid forms.

### An area of mobilizable diversity for AAA triploids

The MSEA zone is the area of origin for various AAA triploids, including the Cavendish Group, which has supplied the majority of the international banana market for over half a century. This group was frequently encountered in our nMSEA study area, along with AAA from the Red Group (4 occurrences) and especially from the Orotava Group (9 occurrences) which is here very popular. These three groups are fairly similar in their genomic profiles and show roughly balanced contributions between the banksii, zebrina, malaccensis subspecies, along with a significant proportion of undetermined segments (unknown gene pools) (pie charts on S2 File, pages 4–6). In contrast, type ‘269’, encountered twice in the zone, has no known equivalent in international collections. It clearly differs from the previous types by a very strong contribution from the malaccensis subspecies, which represents nearly half of the genome (pie charts on S2 File, page 7).

It may be assumed that these triploids followed the diffusion of diploids from the southern areas of MSEA. Local creation of AAA in the northern MSEA zone, from imported exogenous AA, cannot be excluded and presently undescribed AAA might be found in the area as elsewhere in Laos, northern Thailand or eastern Myanmar.

A series of AAA triploids has thus formed in this MSEA zone, both north and south, alongside the Cavendish/Gros-Michel types. Their potential residual fertility could make them resources of interest for banana breeding. They might also offer immediate alternatives to the perilous Cavendish monoculture, which is now under severe biotic constraints. In the same way that in the mid-20th century, Cavendish provided an alternative to the Gros-Michel type decimated by a fungal disease, Fusarium wilt, knowing that a new variant of this fungus, Tropical Race 4, is now decimating the Cavendish in turn.

### Reassessing the contribution of burmannica subspecies

The native *M. acuminata* subsp*. burmannica* was commonly thought to be minimally involved in cultivated bananas, for example, contributing to only 4 of the 60 genomic profiles analysed by Sardos and colleagues [52]. This subspecies is in fact very present locally in di- or triploid types resulting from hybridisations with various exogenous AA.

To describe the genomic constitution of the different specimens, the following notation has been adopted: A_e_ for an exogenous haplotype, i.e., mixture of various *M. acuminata* subspecies except burmannica, A_b_ for a pure burmannica type acuminata haplotype, and A_eb_ for a recombinant haplotype of A_e_ and A_b_.

#### Burmannica contribution to cultivated AA.

**Fig 7:**
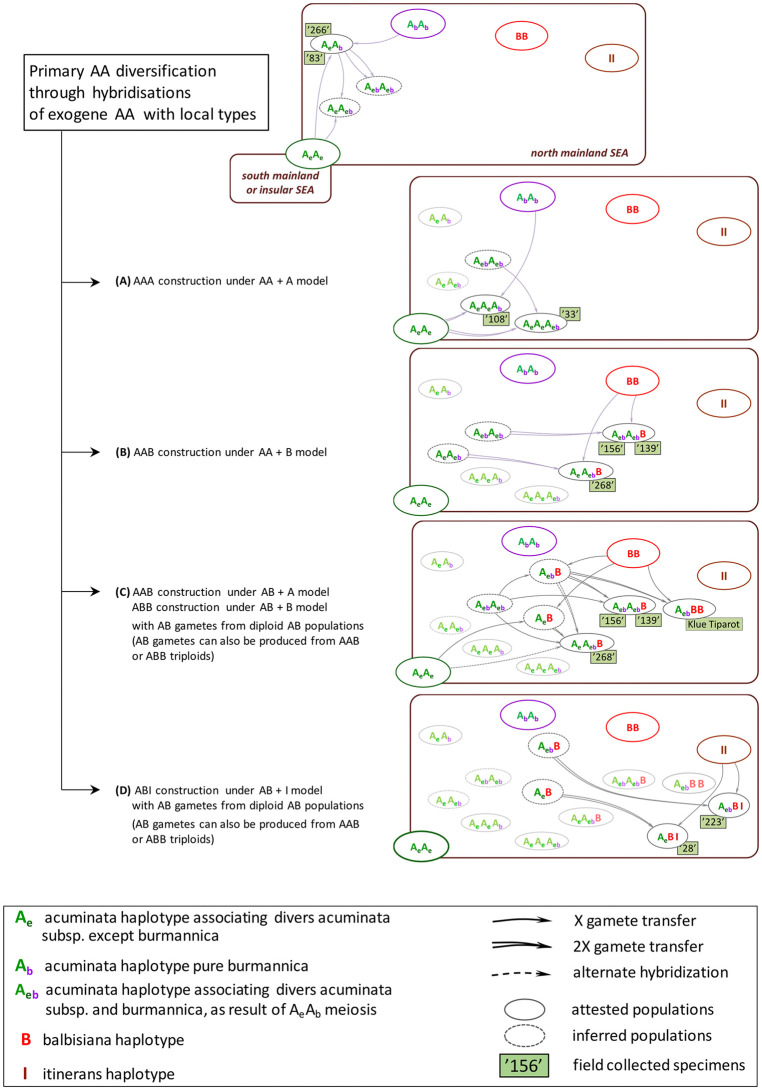
Models for cultivated diploids and triploids construction with integration of local wild forms. A_e_: acuminata haplotype associating various *M. acuminata* subspecies except burmannica; A_b_: pure *M. acuminata* subsp. *burmannica* haplotype; A_eb_: haplotype associating *M. acuminata* subsp. *burmannica* and other subspecies as result of A_e_A_b_ meiosis; B: *M. balbisiana* haplotype; I: *M. itinerans* haplotype.

At the first generation, hybridisation of *M. acuminata* subsp*. burmannica* with exogenous A_e_A_e_ produced A_e_A_b_ forms (Fig 7). The AA type ‘266’ and type ‘83’ are two representatives of these F1 populations. In both cases, the burmannica haplotype is complete, with no trace of recombination with the other acuminata haplotype. This second acuminata haplotype differs between the two types, although several chromosomes are quite similar, or even identical, such as chromosomes 2, 5, or 10, indicating a close relationship between the mobilised exogenous AA.

These A_e_A_b_ forms are otherwise little known. From the SNPs data available from accessions of the International Transit Center collection [10], the genomic profiles of various specimens were established using an identical method to that used here. The Tuu Gia accession (ITC0610) from Vietnam presents a complete burmannica haplotype; the other haplotype is banksii, with its schizocarpa and zebrina insertions, but without malaccensis contribution (S1 Fig – A). The Matti accession (ITC1761) also presents a complete burmannica haplotype, but the other acuminata haplotype has a very strong malaccensis contribution (S1 Fig – B). This variety is widely cultivated in the southern regions of India, Tamil Nadu and Kerala, where the sweet fruit is used as baby food and for its medicinal properties [60]. It is interesting to note that the presence of a complete burmannica haplotype does not seem to modify fruit quality, while the resistance to leaf spot diseases credited to Matti could originate from this burmannica genome [61]. This highlights the potential of closely related wild species, in this case *M. acuminata* subsp*. burmannica*, for managing tolerance to (a)biotic stress in breeding programmes. The ITC collection also includes accessions ITC0069 and ITC0727, which are artificial creations from CIRAD improvement programme; their interest lies in showing that burmannica by zebrina hybrids are possible and viable although the fruits do not appear to contain viable seeds, confirming the immediate sterility induced by intersubspecificity (S1 Fig – C).

Recombined A_eb_ haplotypes are present in several of the triploid forms collected. These A_eb_ haplotypes may originate from a recombined unreduced 2N gamete from a diploid F1 hybrids A_e_A_b_ parent. It is also possible that recombined N gametes from F1 A_e_A_b_ hybrids give rise to A_eb_A_eb_ populations, or A_e_A_eb_ through backcrossing with an exogenous AA. These recombined AA were not observed in the collected specimens, they are, however, documented, such as the Pulutan accession (ITC1775) from the Philippines, which effectively presents a recombinant burmannica haplotype (S1 Fig – D). The Manang accession (ITC1771), popular in the Bicol region in the Philippines, presents a very similar profile [52].

#### Burmannica contribution to cultivated AAA.

The complex genomes of these exogenous AA lead to disrupted meiosis and the formation of unreduced 2x gametes, which locally associated with burmannica x gametes to generate A_e_A_e_A_b_ triploids specific to the nMSEA zone (Fig 7A).

**Fig 8:**
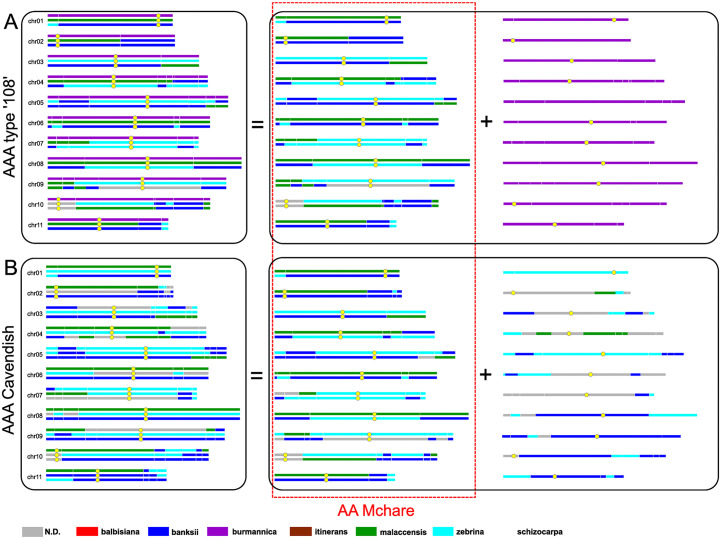
AA Mchare as 2x gamete donor in AAA triploid formation. (A): Decomposition of the AAA type ‘108’ by subtraction of the x gamete from *M. acuminata* subsp*. burmannica*, leaving a 2x gamete of AA Mchare type. (B): Decomposition of AAA Cavendish on AA Mchare as 2x gamete and an unidentified AA as x gamete (with just a few local inversions due to arbitrary phasing choices made in the Cavendish profile).

The AAA type ‘108’, collected four times in Vietnam and Laos, associates a non-recombined burmannica genome with the genome of an exogenous AA identical to the reference 912 AA ‘Akondro Mainty’ (Fig 8A). This AA Akondro Mainty, of Madagascan origin, is a representative of the Mchare AA group also cultivated in the Comoros and very widely in East Africa, particularly in Tanzania [62]. It had been shown that this Mchare genome was fully conserved either by apomeiosis or strict first-division restitution (FDR) [63], in the AAA Cavendish and Gros-Michel genomes originating from the southern MSEA zone [64]. Our data confirm that the genomic profile of AAA Cavendish indeed integrates the ‘Akondro Mainty’ genome (Fig 8B). It had also been shown that this Mchare genome had provided the acuminata haplotypes of the AAB of India, Pome and related forms Nadan and Nendra Padaththi [22,65]. Our AAA type ‘108’ thus indicates that in this MSEA zone, alongside Cavendish and Gros-Michel, other AAA formed from an AA Mchare type as unreduced 2x gamete, with various x gametes. This AA Mchare has not been found in Asia to date, even though its past footprint is evident there [15]. It has moved extensively, passing through this nMSEA zone to reach India; it hybridised along the way with local AA but also with wild acuminata forms such as burmannica or even with *M. balbisiana* in India. This type ‘108’, or other AAA with the same mode of formation, should be explored as alternatives to Cavendish, retaining similar fruit quality but incorporating resistance traits from burmannica.

The AAA type ‘33’, collected in Vietnam and northern Laos, also presents a haplotype with burmannica contribution, but largely recombined here (S2 File, page 9). The construction is of type A_e_A_e_ + A_eb_, associating, as in the previous case, a 2x gamete from an exogenous AA and an x gamete from the local recombined A_e_A_eb_ population. The exogenous AA involved is original; it is not identifiable with Mchare as in the previous case, nor as the AA Sucrier that has been found under cultivation in the area. It is also not found as a constituent of other triploids. Note that another mode of construction is possible, associating a 2x gamete A_e_A_eb_ and an x gamete from an A_e_A_e_. However, this would only be possible for type ‘33’, since for type ‘108’, the contribution of A_e_A_e_ Mchare is established.

#### Burmannica contribution to cultivated AAB.

*Musa acuminata* subsp. *burmannica* also contributes to the formation of interspecific AAB triploids. Three different landraces have been found under cultivation in the area: types ‘139’, ‘156’ and ‘268’. In all three cases, a complete balbisiana haplotype is found, with just some exchanges at chromosome ends, likely due to mitotic recombination. A second haplotype is clearly burmannica, with for each type complete haplotypes for several chromosomes, the other chromosomes being recombined with other *M. acuminata* subspecies. The third haplotypes come from three different exogenous AA: type ‘139’ (S2 File, page 14) is exclusively banksii and zebrina, whereas types ‘156’ (S2 File, page 15) and ‘268’ (S2 File, page 16) also show contributions from the malaccensis subspecies. Interestingly, burmannica segments are observed on this third haplotype for types ‘139’ and ‘156’, which are therefore already recombined and thus of form A_eb_A_eb_B, whereas ‘268’ would be A_e_A_eb_B. In an AA + B scheme (Fig 7B), local *M. balbisiana* would provide an x gamete associating with 2x gametes A_eb_A_eb_ and A_e_A_eb_ already mentioned for the AA and AAA types.

An AB + A scheme is also possible (Fig 7C), supposing the prior formation of AB diploid gametes: A_eb_B + A_eb_ for ‘139’ and ‘156’, A_eb_B + A_e_ or A_e_B + A_eb_ for ‘268’. These diploid AB gametes may be unreduced gametes of hybrid AB forms. They may also be produced by triploids AAB or ABB. This issue will be revisited later for the formation of triploids ABB and ABI.

#### Burmannica contribution to cultivated ABB.

*Musa acuminata* subsp*. burmannica* is also found in interspecific ABB triploids. The four specimens of Klue Tiparot collected in the area, three in northern Laos and one in Vietnam at the Laotian border, show an acuminata haplotype mobilising the banksii and burmannica subspecies, the two genotypes observed differing in the extent of balbisiana intrusion into the acuminata haplotype (S2 File, pages 24 and 25). The significant burmannica contribution leads to proposing an origin in our study area, nMSEA. The ‘Tiparot’ accession from several *ex situ* collections and from ITC (ITC0652) is of Thai origin, the name ‘Tiparot’ being a corrupted form of the Thai name ‘Kluai Theppharot’, which means ‘banana of divine taste’ [58]. Its synonym would be Pisang Abu Siam, the qualifier Siam referring to its area of origin. The Ya Ta Na Thin Kha accession from the ITC collection (ITC1755), which presents an acuminata genomic profile of the same origine as our Tiparot type [52], comes from Pyinmana, former name of the current capital of Myanmar, about 200 km west of the northern Thailand border. The two Klue Tiparot specimens referenced in the MGIS In Situ system (https://www.crop-diversity.org/mgis) also come from northern Laos. All these elements argue for a local origin, in the northern MSEA zone.

The joint presence of banksii and burmannica indicates a recombined origin of the acuminata haplotype, and thus an A_eb_BB type for Klue Tiparot. Its formation would therefore involve an A_eb_B 2x gamete and an x gamete from a local BB. The A_eb_B 2x gamete may be a non-reduced gamete produced by a locally formed diploid AB or a 2x gamete from A_eb_A_eb_B or A_eb_BB triploids (Fig 7C). An alternative hypothesis of BB as 2x gamete and a local A_eb_A_eb_ as x gamete could also be considered; however, the production of 2x gametes by *M. balbisiana* or an ABB variety has never been demonstrated and should have led to the formation of BBB triploids, which are not currently attested.

### First evidence of *M. itinerans* shaping banana diversity

**Fig 9:**
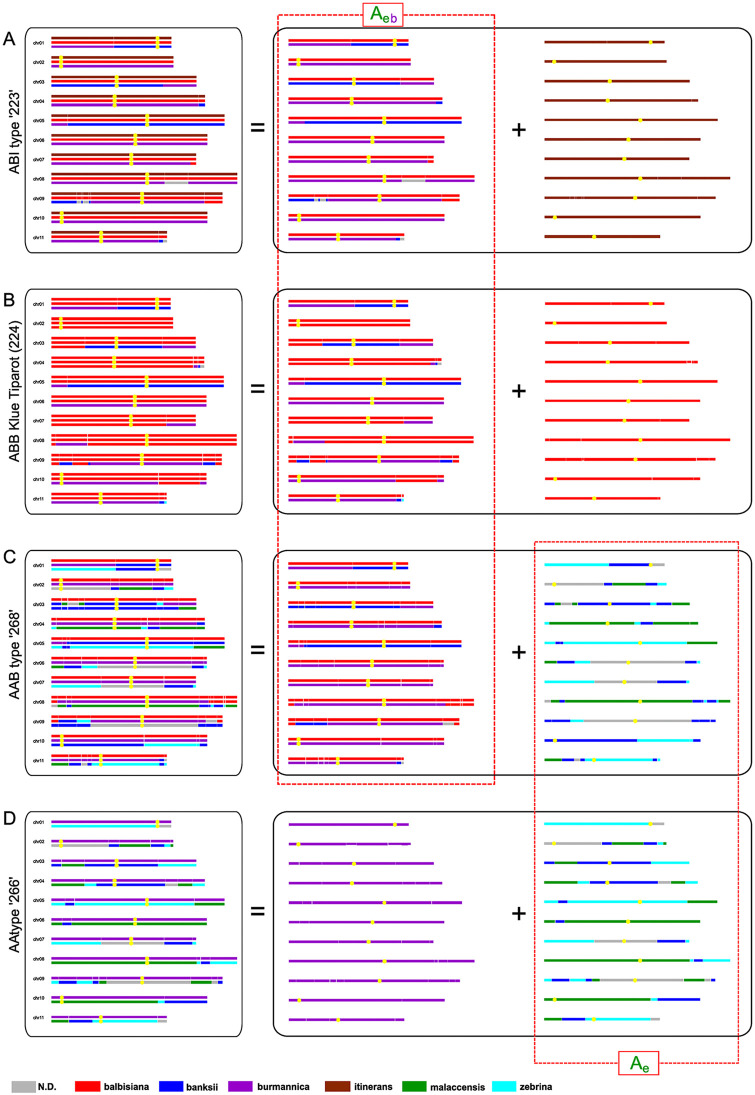
Decomposition of genomic profiles on parental gametes. (A): extraction of the *M. itinerans* haplotype as x gamete from the ABI type ‘223’ genome, leaving a 2x gamete from a local A_eb_ hybrid between an exogenous AA and *M. acuminata* subsp*. burmannica*. (B): the same 2x gamete is found in ABB Klue Tiparot (although locally altered by balbisiana insertions), associated with a balbisiana x gamete. (C): a similar 2x gamete can be extracted from AAB type ‘268’, leaving an x gamete from an exogenous AA. (D): The x gamete from an exogenous AA found in the above decomposition of AAB type ‘268’ is very similar to the exogenous gamete associated with a burmannica genome in AA Kouay type ‘226’.

As a major finding of this study, *Musa itinerans*, whose contribution to cultivated forms had never been documented before, appears in several triploid and tetraploid forms. The ABI ‘type 223’ specimen is highly relevant for tracing the development of these interspecific hybrids. The absence of any recombination along the itinerans haplotype, whilst observing some balbisiana introgressions in the acuminata haplotype, supports the hypothesis of hybridisation between an x itinerans gamete and a 2x AB gamete. Under this hypothesis, the ABI ‘type 223’ genome can be split into its itinerans and AB components. (Fig 9A). The acuminata genome of this AB gamete appears to be primarily of burmannica origin. However, banksii segments on at least 4 chromosomes indicate an A_eb_ form, with contribution from an exogenous AA with strong banksii component. For type ‘28’, the acuminata component shows no burmannica contribution and is of A_e_ form originating from an exogenous AA.

These unreduced A_eb_B or A_e_B gametes would originate from AB diploids (Fig 7D) that we can postulate formed locally. This assumes the formation of interspecific AB diploids, as previously proposed for the development of ABB Klue Tiparot, from local balbisiana and exogenous A_e_A_e_ populations (for ABI type ‘28’) or A_e_A_eb_ or A_eb_A_eb_ hybrids with the local burmannica population (for ABI type ‘223’). As previously mentioned, it is also possible that these 2x gametes are produced by triploid A_eb_BB, or even A_e_A_eb_B or A_eb_A_eb_B, known to be present in the area.

The ABBI forms directly derive from ABI forms through the addition of a balbisiana genome, either by endoreplication of the B chromosomes, although simultaneous endoreplication of all 11 chromosomes seems highly improbable, or by formation of unreduced triploid gametes ABI and hybridisation with a B gamete. The production of triploid gametes by apomeiosis or strict FDR [55] is widely attested by the presence of tetraploid forms in the offspring of crosses between triploids and diploids [66,67].

These hitherto unidentified ABI forms are nevertheless already represented in the ITC international collection. The Chuoi Xi Mon accession (ITC1386) introduced from Vietnam, which is designated ABX with an unknown X genome, shows an AB genomic composition [52] identical to our ABI type ‘28’, strongly suggesting that the unknown haplotype named X is in fact *M. itinerans*. However, the ABI ‘223’ form should also be introduced in the ITC collection, as well as a tetraploid ABBI.

### Diversification patterns evidenced by genetic kindships between banana landraces

The potential of SNPs to construct well-established phylogenies is known, including for bananas. It is even possible to go beyond phylogenetic evidence and explore the genealogies of these cultivated forms by identifying direct relationships between particular genotypes.

It has been shown above, for example, that the same AAcv Mchare was involved in the creation of AAA Cavendish or Gros-Michel as well as the local AAA triploid type ‘108’ (Fig 8).

The ABI type ‘223’ is also particularly interesting as it allows tracing relationships with several other types. Subtraction of the itinerans haplotype leaves an A_eb_B genome (Fig 9A). This same A_eb_B is found when decomposing AAB type ‘268’ (Fig 9C), with the same highly characteristic signature of recombination points between banksii and burmannica. The x gamete here is not itinerans but comes from an AA with banksii, zebrina and malaccensis components (A_e_A_e_) of exogenous origin introduced into the area.

Moreover, this same A_eb_B component can be surmised when decomposing the local ABB triploid Klue Tiparot into AB + B (Fig 9-B), even though balbisiana substitutions in the acuminata haplotype mask a number of characteristic recombination points. However, the accession ’Ya Ta Na Thin Kha’ (ITC1755) [52], mentioned above as having a similar acuminata origin, does indeed show this same A_eb_B component.

The mobilisation of the same 2x AB gamete in very different genomic contexts provides insights for the modes of construction of the AAB, ABB, and ABI triploids mentioned above. Indeed, the diploid gametes produced by a triploid are necessarily recombined, and it is therefore highly improbable that the same AB gamete would occur three times by chance in AAB, ABB, and ABI contexts. This supports the hypothesis of an unreduced and non-recombined gamete produced by a local AB diploid, in the same way that the AA gamete of several AAA is produced by the intersubspecific diploid variety AA Mchare.

The mobilised interspecific AB diploids were presumably cultivated forms, widely distributed to be so frequently involved in the construction of new cultivars, and likely sterile, or at least exhibiting disrupted meiosis, to readily produce 2x gametes. However, these AB forms have not been described and were not found during the investigation. Diploids AB are mainly known in India, where they are widely cultivated: Safet Velchi, Kunnan, Ney Poovan... These genomes do indeed show balanced acuminata and balbisiana chromosomes, but with local segments that are purely acuminata, indicating backcrossing with an acuminata diploid [68]. However, overall, except for recent discoveries in Southeast Asia [69], cultivated AB forms are rare: forms of lesser agricultural or gustatory interest, greater susceptibility to disease?

Nevertheless, in some other cases, the triploid origin of the AB gamete cannot be ruled out, despite the very low fertility of triploids. The production of AB gametes by an ABB triploid allows for recombinations that could explain the presence of several balbisiana centromeres in the third haplotype of the two Klue Tiparot variants. Thus, ’Ya Ta Na Thin Kha’ was even proposed as potential ABB donor of 2x gametes for ‘Klue Tiparot’ [52]. An indirect argument would also be that it no longer requires the existence of these unidentified AB populations. It has recently been shown [65] that for most triploid cultivars, the parents detected as most probable by their genome are diploids. However, for at least two triploids, ABB Saba and AAB Mnalouki, the hypothesis of a triploid parent was the most highly supported.

In summary, the hypothesis of diploid gamete production by diploids seems to be accepted. However, this does not rule out the possibility that their production by triploids may have coexisted locally.

Coming back to the AAB type ‘268’ specimen, subtraction of the previous AB genome leaves an A genome with triple banksii, zebrina and malaccensis components, with notable segments from unknown pools, which shows very strong similarities with the complementary acuminata x gamete of the burmannica gamete in the genome of AA ‘Kouai’ type ‘266’ (Fig 9-D). Both could have the same exogenous cultivated AA as parent, or two genetically closely related AA. This AAB type ‘268’ also leads to the conclusion that both processes are possible for AAB formation: AB + A for this type ‘268’ and more probably AA + B for types ‘139’ or ‘156’ mentioned previously.

### Several possible routes for exogenous AA dispersal in MSEA

The dispersal routes of exogenous AA cultivars arriving in the MSEA region are now partially understood. The narrative begins in New Guinea, where the local *M. acuminata* subsp. *banksii* hybridised with the endemic *M. schizocarpa* species [19]. Banksii forms, introgressed with several schizocarpa segments, exhibited sufficiently interesting characteristics for human selection and dissemination. Notably, parthenocarpy—a major fruit domestication syndrome alongside sterility—shows early indications in some wild banksii forms [70]. These initial cultivars were subsequently introduced westward, interacting with *M. acuminata* subsp. *zebrina* populations from Java, and probably also from Sulawesi and southern Kalimantan. This interaction generated hybrid forms associating banksii (with its schizocarpa introgressions) and zebrina genomes, observed both in the diploid state and integrated into AAA or AAB triploid.

Further north, via Sumatra and towards the Malay Peninsula and southern mainland SEA, these hybrids further diversified by hybridising with local *M. acuminata* subsp. *malaccensis* and possibly other local acuminata genomes like *M. acuminata* subsp. *halabanensis*. Several of these hybrids, characterised by their malaccensis genomic segments, are documented in our northern MSEA zone, either in diploid form or through their contribution to mono- or interspecific triploids.

Intriguingly, locally originated triploids such as ABI, ABB Klue Tiparot, and AAB type ‘139’, which likely share a common 2x AB gamete, show no malaccensis contribution. The diploid ‘Tuu Gia’ from Vietnam, presenting a complete burmannica haplotype without malaccensis input (S1 Fig), further substantiates this observation.

These findings suggest an alternative introduction pathway in northern MSEA for exogenous AA genomes without malaccensis contribution. A plausible alternative route emerges via southern China and Taiwan, regions with high prevalence of acuminata, balbisiana, and itinerans genomes. Consequently, a second potential AA migration pathway can be inferred, traversing eastward through the Philippines, where ABB types like Bluggoe and Saba, similarly devoid of malaccensis contributions, are widespread. The Austronesian expansion was followed by the emergence of complex maritime trade routes over the last millennium BCE. One such route may have linked New Guinea to the Asian mainland via the Moluccas and the Philippines. Thus, cloves, produced in the Moluccas (Spice Islands), were already in use at the Chinese imperial court, in the final centuries BCE [71].

### Mobilising local related genomes: new adaptive potential or neutral variation?

Conventionally, crop domestication is associated with genetic bottlenecks and diversity loss compared to original wild forms. However, banana domestication reveals a contrasting narrative—a process of diversity gain through successive incorporations of genomic material from encountered wild relatives during the expansion of the geographical range. Notably, ABI forms exhibit a record number of involved genomes, with haplotypes of *M. balbisiana*, *M.* *itinerans*, and *M. acuminata* origin, itself presenting contributions from the local burmannica subspecies, the zebrina subspecies and the banksii subspecies, known itself to include segments of *M. schizocarpa*.

Founding hybridisation in sympatric habitats between two, or more, wild relatives, often accompanied by allopolyploidisation, is recognized as a crop development mechanism, such as the well-known example of the various wheat sub-genomes [72]. However, banana plants follow a very different interspecific hybridisation process where a domesticated plant hybridises with a local wild relative. Such secondary hybridisations have been shown to be key in shaping the diversity of various species, including rice (*Oryza sativa*) [73], date palm [74], grape [75], and almond [76].

The rationales for this cumulative diversification through the incorporation of heterologous genomic segments raise questions. A primary interpretation suggests the generation of increasingly well-adapted forms by pyramiding genes of interest. These introgressions could contribute to a ‘post-domestication’ process facilitating better adaptation to novel local conditions encountered during geographical expansion from the centre of origin [77,78]. This multi-level stacking is facilitated in bananas by polyploidy and vegetative propagation, which fixes valuable combinations. This stacking could include genes for disease resistance, adaptation to abiotic constraints, as well as organoleptic qualities. The exceptional adaptability of a number of successful cultivars, especially triploids, found in various intertropical regions -AAA Cavendish, AAA Red, AAB Plantain, ABB Bluggoe, etc.- might be explained by this cumulative process.

Conversely, another interpretation would suggest that in regions rich in endemic *Musa* species, hybridisation with genetically close introduced cultivated forms is highly probable. When incorporating novel genomic sequences does not induce manifest disadvantages, such genetic material may persist or even spread if it coincidentally confers a distinctive secondary trait. The ABI forms encountered in the region closely resemble ABB cultivars and do not appear to exhibit strong specific characters. The reasons why local farmers cultivate and preserve these original varieties could only be clarified by conducting an extensive survey and running field evaluations.

Regardless, these incorporations of wild relatives, whether beneficial or neutral, are readily accepted by local populations. Consequently, this argues for incorporating more widely these wild relatives into breeding programmes. For instance, *M. itinerans* could be strategically mobilised as a resistance source, notably to Fusarium race 4 [79], through hybrids with *M. acuminata* and *M. balbisiana*. The demonstration that such triploid forms are not only possible but have been traditionally cultivated further validates this approach.

## Conclusions

The primary contribution of this study is the identification of locally cultivated forms that incorporate endemic wild relatives. Notably, the contribution of *M. acuminata* subsp. *burmannica* is significantly more prevalent than previously assumed, while the contribution of *M. itinerans* represents a major finding of this survey. The pre-domestication status observed for these local wild species [33] may have facilitated their interaction with cultivated forms, occasionally leading to the formation of hybrids that farmers adopted and propagated vegetatively. Conversely, *M. yunnanensis*, despite being genetically closer to *M. acuminata* and capable of spontaneous hybridisation, has not been recruited into the domestication of cultivated forms. And indeed, the previous results had shown that local populations make only limited use of this species.

Even though the sampling procedure does not allow for a precise estimate of their relative contribution to banana production in northern MSEA, these original local forms are not merely improbable crossings, sporadically maintained through vegetative propagation. The dispersal of most of these hybrids across geographically distant regions, often remote from the mixing zones of major urban centres, demonstrates that they are sufficiently valued to be identified, named, and disseminated, even over long distances.

A critical observation is that the diversity represented in major *in vitro* or field collections, which form the basis of diversity studies and breeding programmes, is not fully representative of the global genetic diversity, as evidenced at least in this study, presenting instead a partial and fragmented picture. Further surveys should be conducted in regions that appear to be less fully documented: southern China, Myanmar, northern Thailand, Kalimantan, Sulawesi, and other Indonesian islands. This conclusion had previously been articulated for the same region regarding wild relatives.

## Supporting information

S1 Table List of specimens analysed. NumID: study identifier. ID VCF: sample identifier in the original VCF file. GBS batch. Species/Genomic composition. Subsp/Group/type: subspecies for wild specimens, Group and type when known, type assignment based on genomic profiles for original local material. Ploidy: estimated from allelic ratios. CRBPT code: accession code in the CRB-PT collection (CIRAD Guadeloupe) for reference specimens. Country. Local name when known. Latitude: decimal latitude. Longitude: decimal longitude (GPS coordinates downgraded for farmers’ fields and gardens). Elevation in m. Environment: the context where the sample was found. Valid SNPs: number of valid SNP for this accession. Mean depth: mean of the depths on all the valid SNPs. H%: heterozygosity. (in grey, the 51 samples added as reference).
(XLSX)

S2 Table Wild samples retained for ancestral pools construction. Same columns as in S1 Table.
(XLSX)

S1 Fig Genomic ancestry profiles for AA diploids with *M. acuminata* subsp*. burmannica* contribution. Redrawn from data by Sardos and colleagues [10].
(PDF)

S1 File Material Transfer Agreements. (PDF)

S2 File Genomic ancestry profiles. The 38 distinct genomic profiles observed on collected specimens and added references: list of specimens belonging to the type, ancestral pool mosaics across all 11 chromosomes, pie charts of the ancestral pool contributions to the complete genome.
(PDF)
