# Supplementary material for: Beyond domestication: The unexpected contribution of native Musa wild relatives to cultivated banana diversity in Mainland Southeast Asia
Source: PLoS One. 2026 Aug 17;21(8):e0352247. doi: 10.1371/journal.pone.0352247 (PMC13480591; doi:10.1371/journal.pone.0352247)
Supplement: Supplementary file 1 [file pone.0352247.s003.pdf]

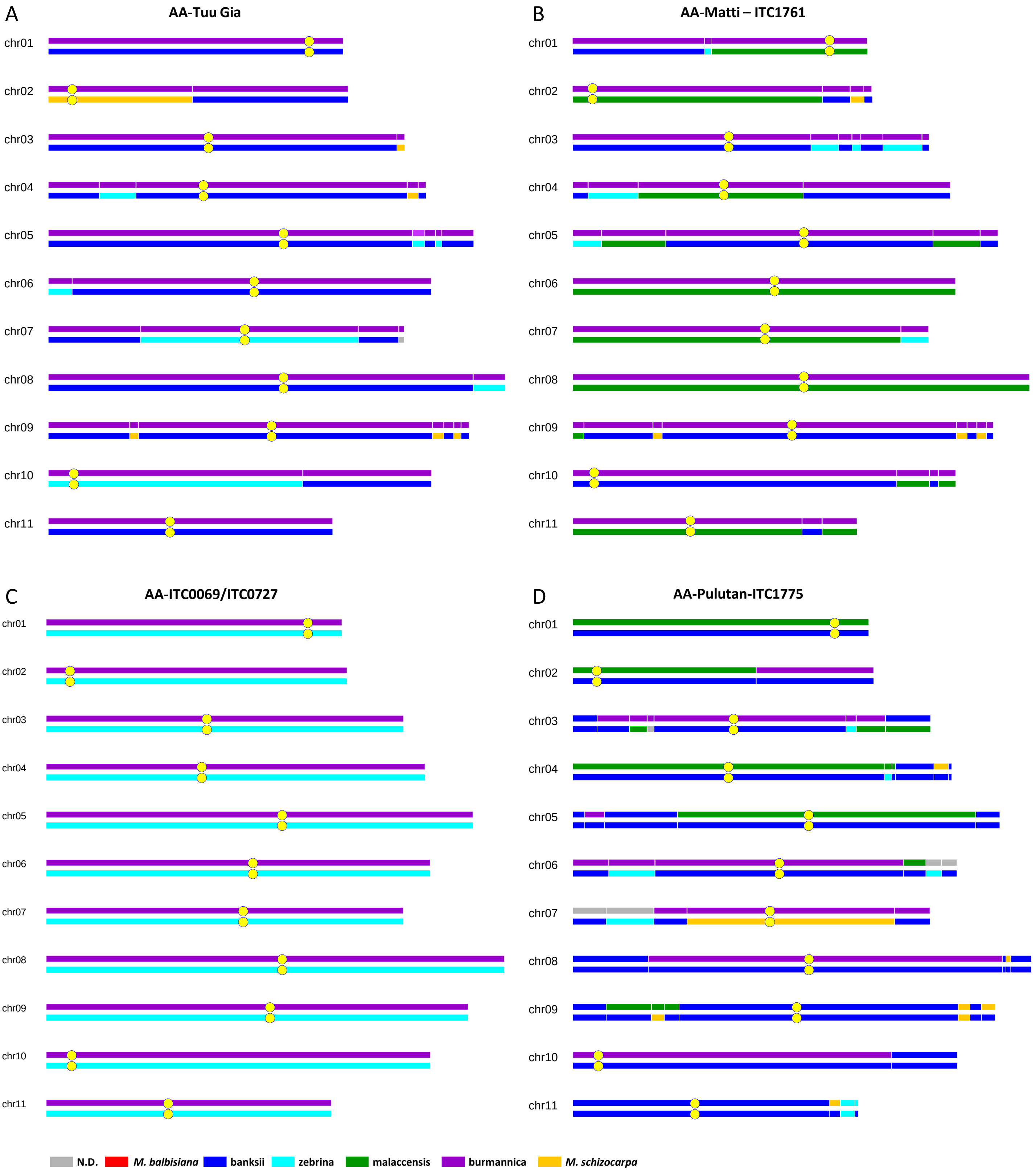
A
AA-Tuu Gia
B
AA-Matti – ITC1761
C
AA-ITC0069/ITC0727
D
AA-Pulutan-ITC1775
chr01
chr02
chr03
chr04
chr05
chr06
chr07
chr08
chr09
chr10
chr11
N.D.
M. balbisiana
banksii
zebrina
malaccensis
burmannica
M. schizocarpa
