# Supplementary material for: Beyond domestication: The unexpected contribution of native Musa wild relatives to cultivated banana diversity in Mainland Southeast Asia
Source: PLoS One. 2026 Aug 17;21(8):e0352247. doi: 10.1371/journal.pone.0352247 (PMC13480591; doi:10.1371/journal.pone.0352247)
Supplement: Supplementary file 2 [file pone.0352247.s004.pdf]

Material Transfer Agreements

For the collecting mission in Vietnam, an application for access to genetic resources was signed in 2018 between one partner from Vietnam (the Institute of Tropical Biology – ITB), the French Agricultural Research Centre for International Development (CIRAD), and the National Museum of Natural History of Paris (MNHN). This document was signed by the three directors of these scientific institutions. ITB was responsible for establishing contracts with the parks and reserves where applicants collected banana samples. CIRAD was responsible for the molecular characterisation of the banana samples, and MNHN was responsible for the long-term storage of the banana samples. Additionally, a collecting permit (No. 1961/TCLN-DDPH) was issued by the Vietnamese Ministry of Agriculture and Rural Development on 9 November 2018.

For the collecting mission in Laos, an application for access to genetic resources was signed in 2018 between the Institute of Tropical Biology (ITB) for Vietnam, the French Agricultural Research Centre for International Development (CIRAD), the National Museum of Natural History of Paris (MNHN), and the Cabinet of the Lao Academy of Science and Technology (CLAST). CLAST is a public establishment responsible for national scientific research projects and the promotion of cultural and scientific activities. Therefore, it was responsible for establishing contracts with the parks and reserves where applicants collected banana samples. The application for access to genetic resources was signed by the four directors of these scientific institutions. Additionally, a Material Transfer Agreement was also signed between the directors of CLAST and CIRAD in March 2019.

For the collecting mission in China, a Material Transfer Agreement was signed in 2019 between the directors of the Kunming Institute of Botany (KIB), CIRAD, and MNHN.

Finally, a consortium agreement for the implementation of each of the two research projects (the Sud Expert Plantes Développement Durable programme through the DivBa SEP2D project (AAP3-97) and the BforBB open science project supported by the Agropolis Fondation under the reference ID 1605-011 through the “Investissements d’avenir” programme (Labex Agro:ANR-10-LABX-0001-01)) that financed the research described in our article was signed by ITB, MNHN, CIRAD, and the Northern Mountainous Agriculture and Forestry Science Institute (NOMAFSI) from Vietnam.
