# Supplementary material for: Beyond domestication: The unexpected contribution of native Musa wild relatives to cultivated banana diversity in Mainland Southeast Asia
Source: PLoS One. 2026 Aug 17;21(8):e0352247. doi: 10.1371/journal.pone.0352247 (PMC13480591; doi:10.1371/journal.pone.0352247)

Catalogue of the 38 genomic ancestry profiles identified

Page	Group	nb specimens
1	AA Sucrier	1 + ref
2	AA type '83'	1
3	AA type '266'	3
4	AAA Cavendish	2 + ref
5	AAA Red	4
6	AAA type '24' - Orotava	9
7	AAA type '269'	2
8	AAA type '108'	4
9	AAA type '33'	2
10	AAB Plantain	1 + ref
11	AAB Pome	1 + ref
12	AAB Mysore	3 + ref
13	AAB Pisang Kelat/Pisang Seribu	4
14	AAB type '139'	2
15	AAB type '156'	3
16	AAB type '268'	1
17	ABB Pisang Awak	10 + ref
18	ABB Pisang Awak variant '343'	1
19	ABBB Pisang Awak 4x	1
20	ABB Bluggoe	ref
21	ABB Bluggoe variant '79'	1
22	ABB Bluggoe variant '149'	2
23	ABB Bluggoe variant '154'	1
24	ABB Klue Tiparot	2 + ref
25	ABB Klue Tiparot variant '224'	1
26	ABI type '28'	4
27	ABI type '28' variant '134'	1
28	ABI type '223'	1
29	ABBI type ‘77'	1
30	ABBI type ‘77’ variant ‘132'	3

Page	Other references
31	AA Mchare
32	AAA Gros-Michel
33	AAA Mutika
34	AAB Silk
35	AAB Laknao
36	AAB Iholena
37	AAB Popoulou
38	ABB Saba

Common legend for all profiles

Colour	Legend
Grey	Not Defined
Red	*M. balbisiana*
Blue	*M. acuminata banksii*
Purple	*M. acuminata burmannica*
Green	*M. acuminata malaccensis*
Cyan	*M. acuminata zebrina*
Brown	*M. itinerans*

Group: AA Sucrier

- specimen 21 ‘Chuoi Ngu’, roadside in Vietnam
- reference 914, ‘Datil’ from CRBP-PT in Guadeloupe (PT-BA-00106)

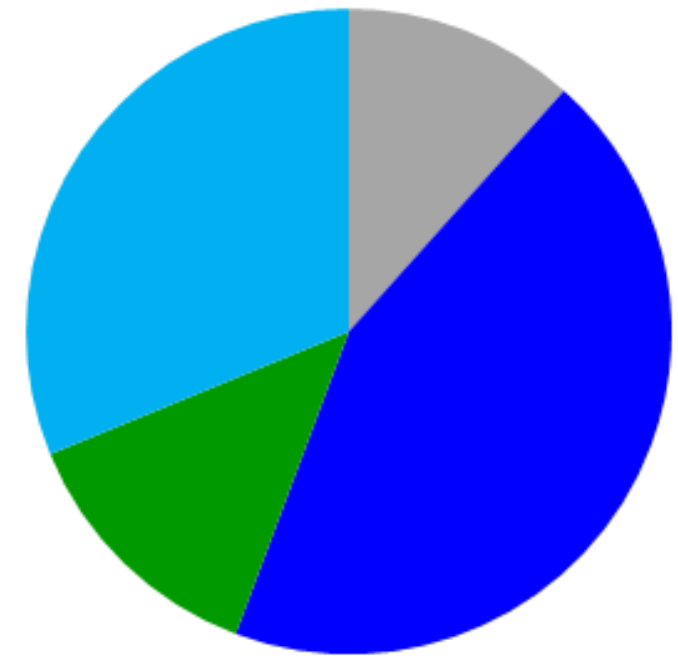

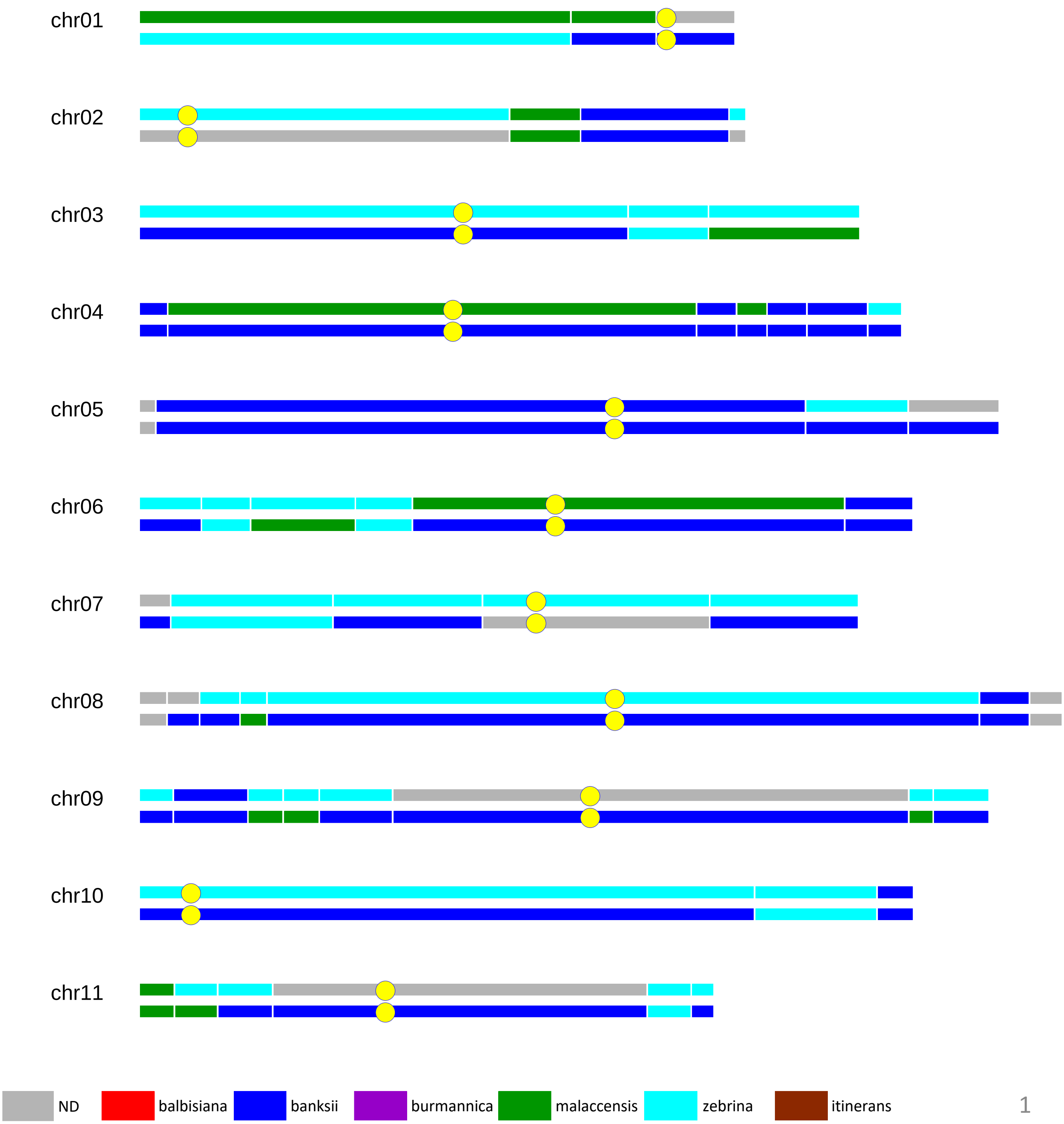

Group: AA type '83'

- specimen 83, botanical garden in Vietnam

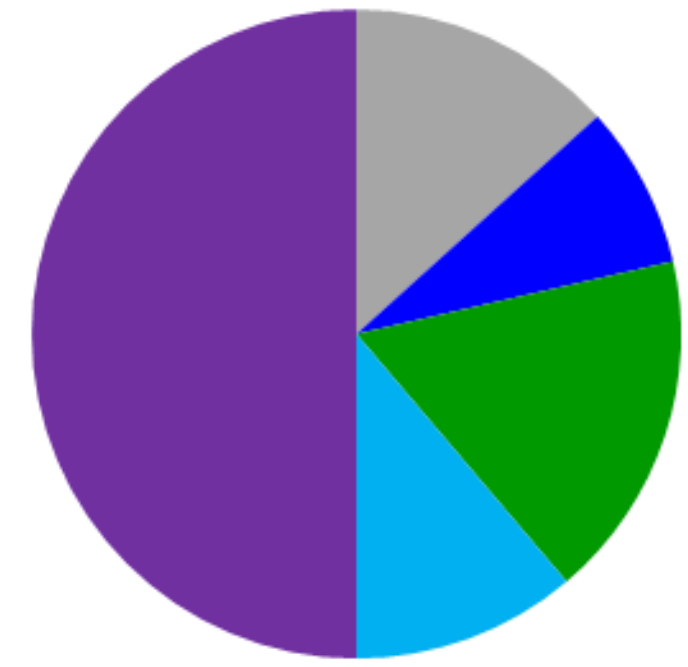

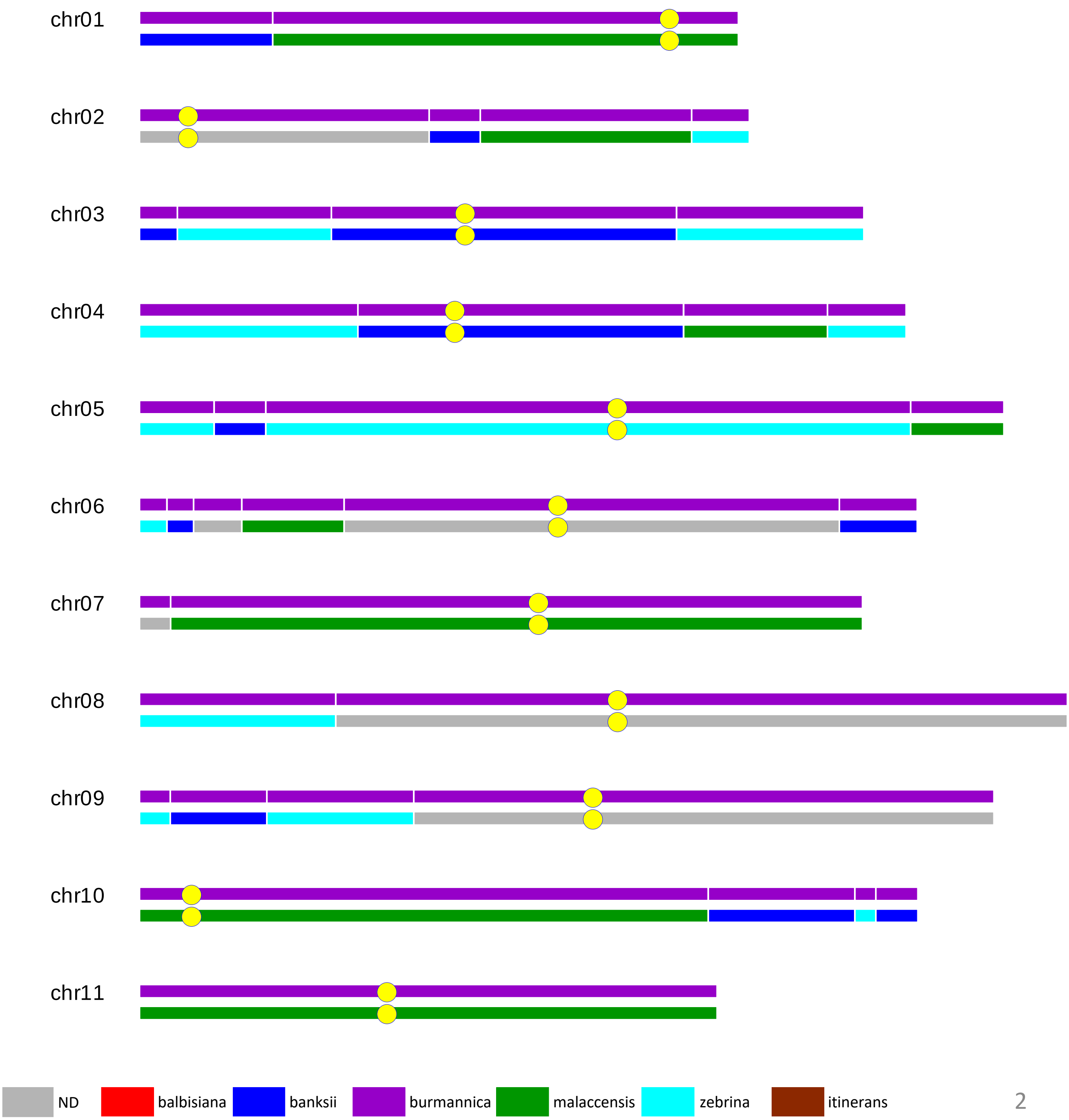

Group: AA type'266‘

- specimens 266 and 282, farmer fields in Laos
- specimen 317, backyard garden in Yunnan

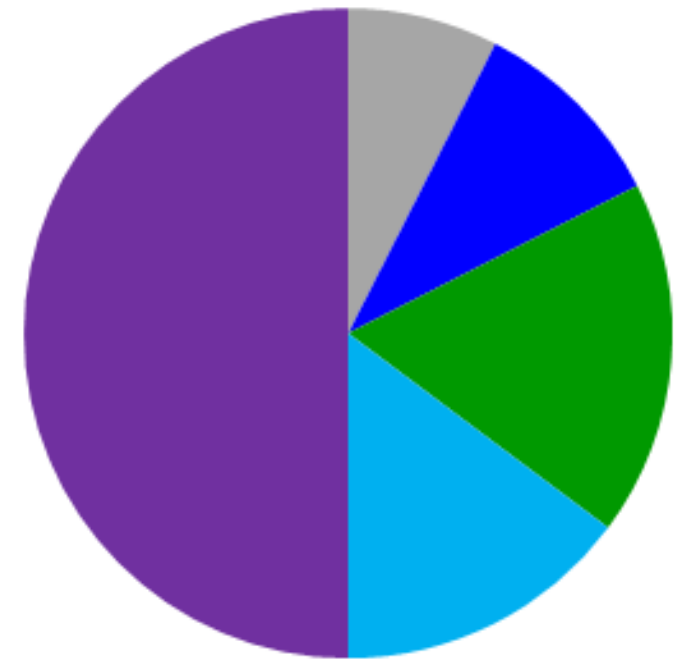

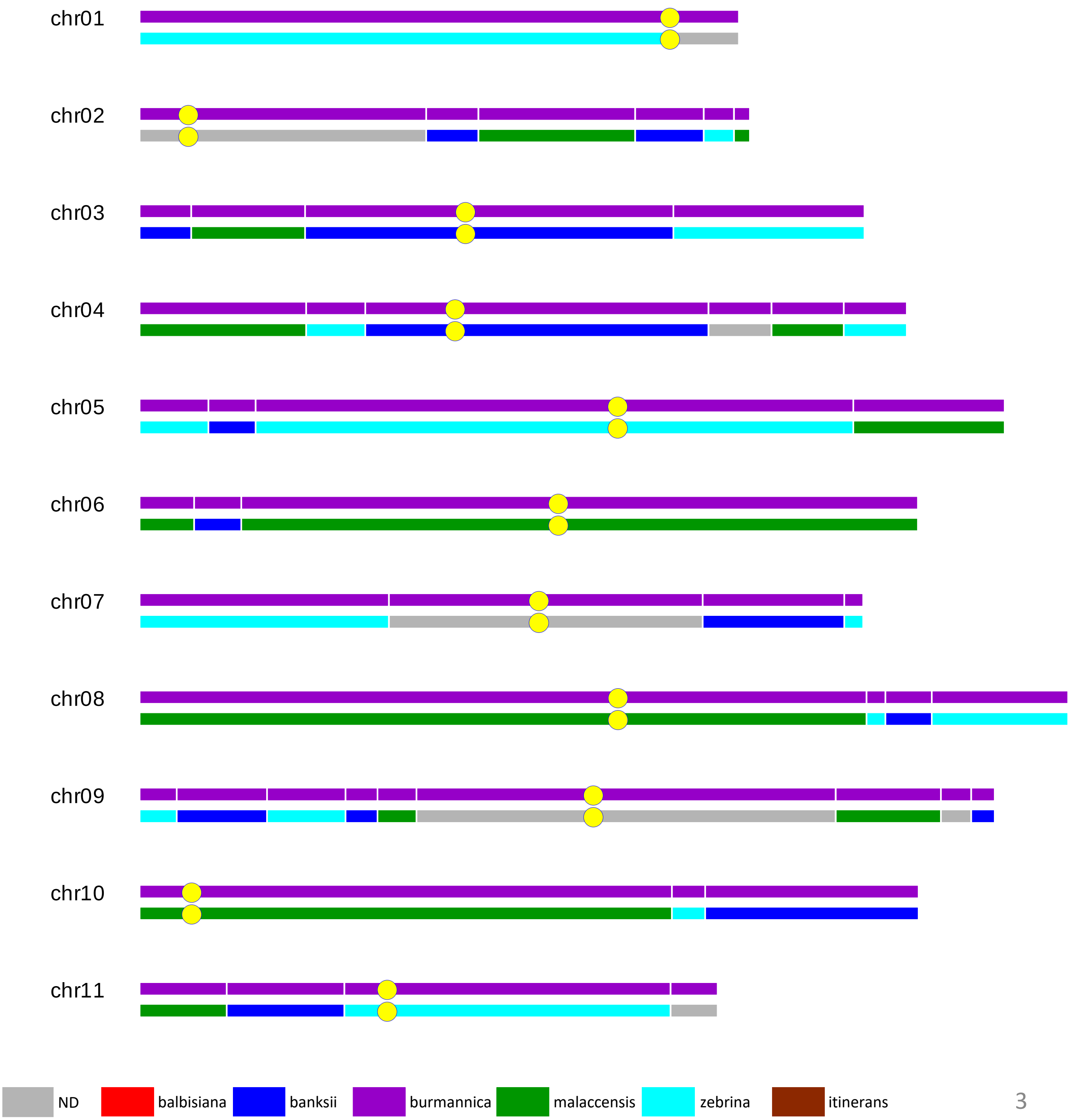

Group: AAA Cavendish

- specimens 119 and 121, farmer fields in Vietnam
- reference 915, 'Grande Naine' from CRBP-PT in Guadeloupe (PT-BA-00104)

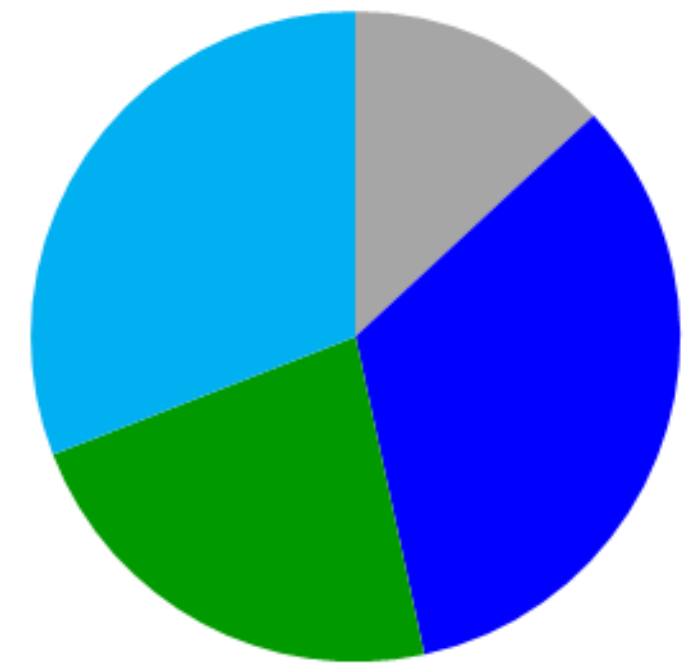

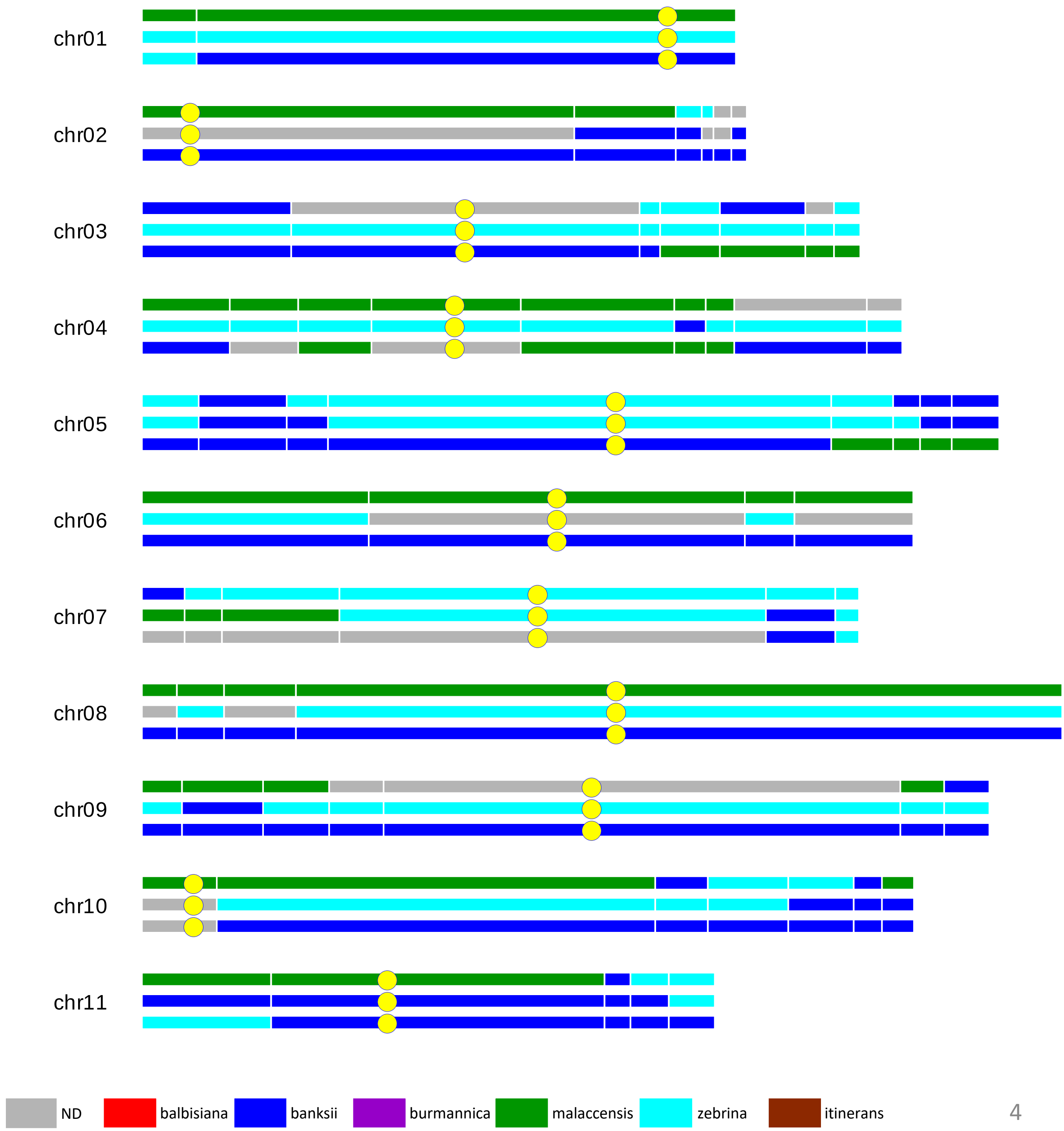

Group: AAA Red

- specimens 65 and 66, roadside in Vietnam
- specimen 67, farmer field in Vietnam
- specimen 240, farmer field in Laos

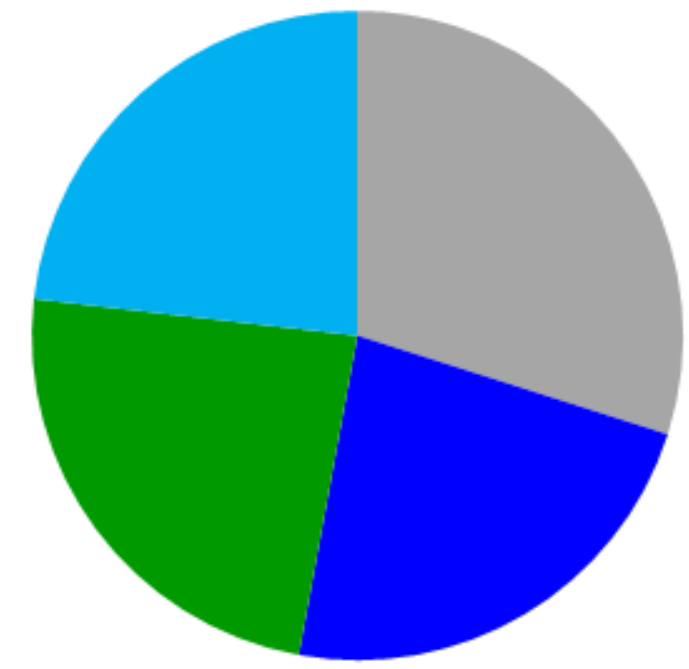

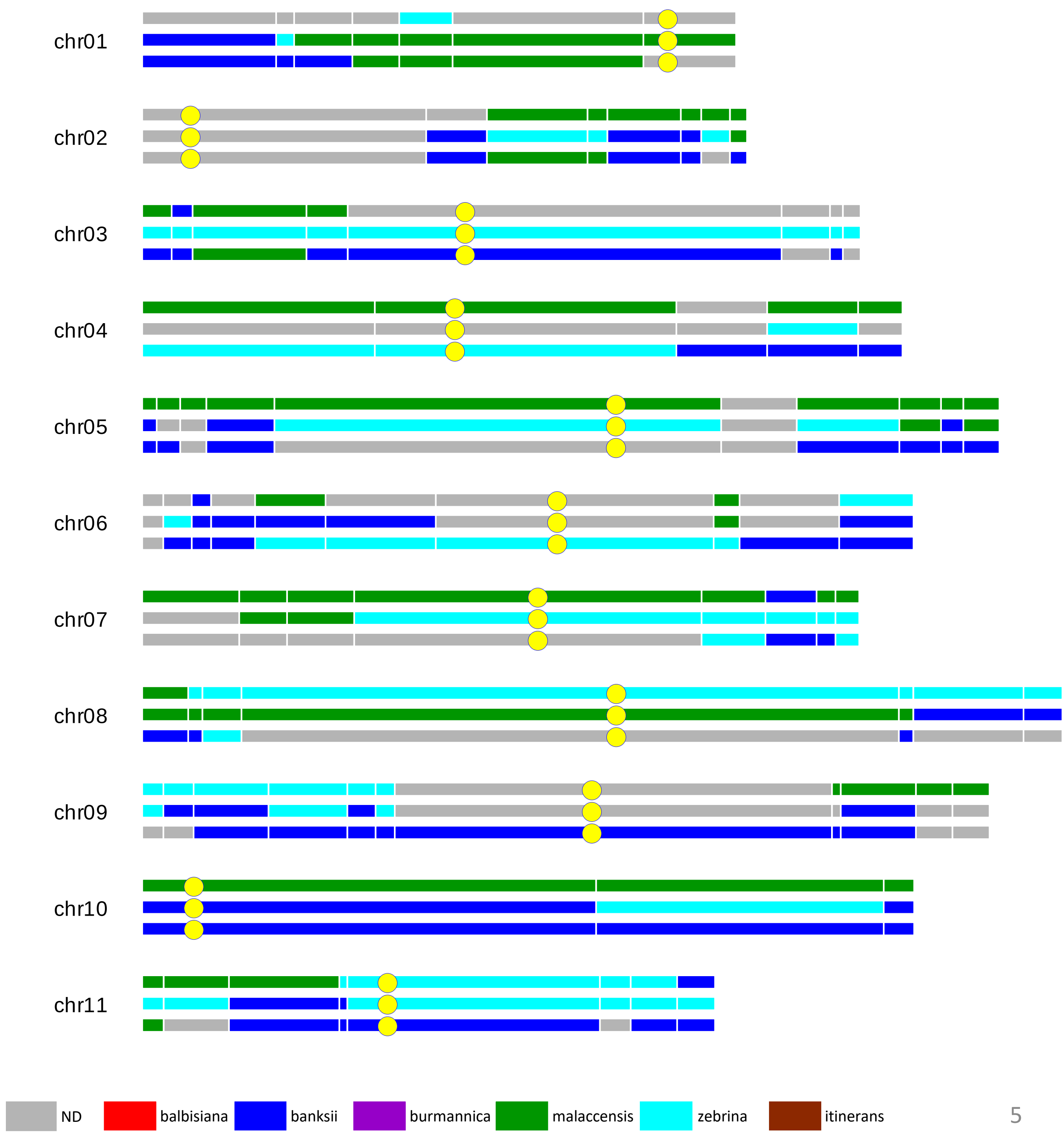

Group: AAA type ‘24’ - Orotava

- specimens 22, 24 ‘Chuoi Mang’, and 105 ‘Chun gong’, farmer fields in Vietnam
- specimen 170, roadside in Vietnam
- specimen 215, roadside in Laos
- specimens 221, 267 and 271, farmer fields in Laos
- specimen 232, backyard garden in Laos

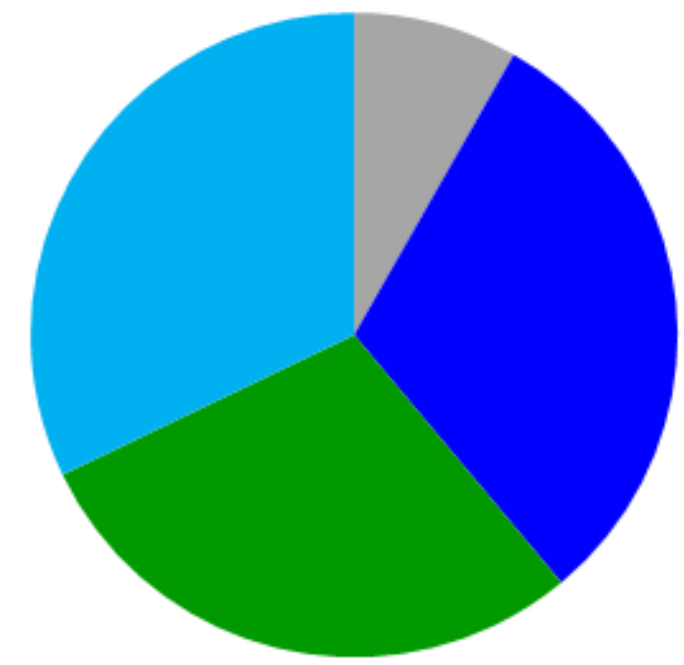

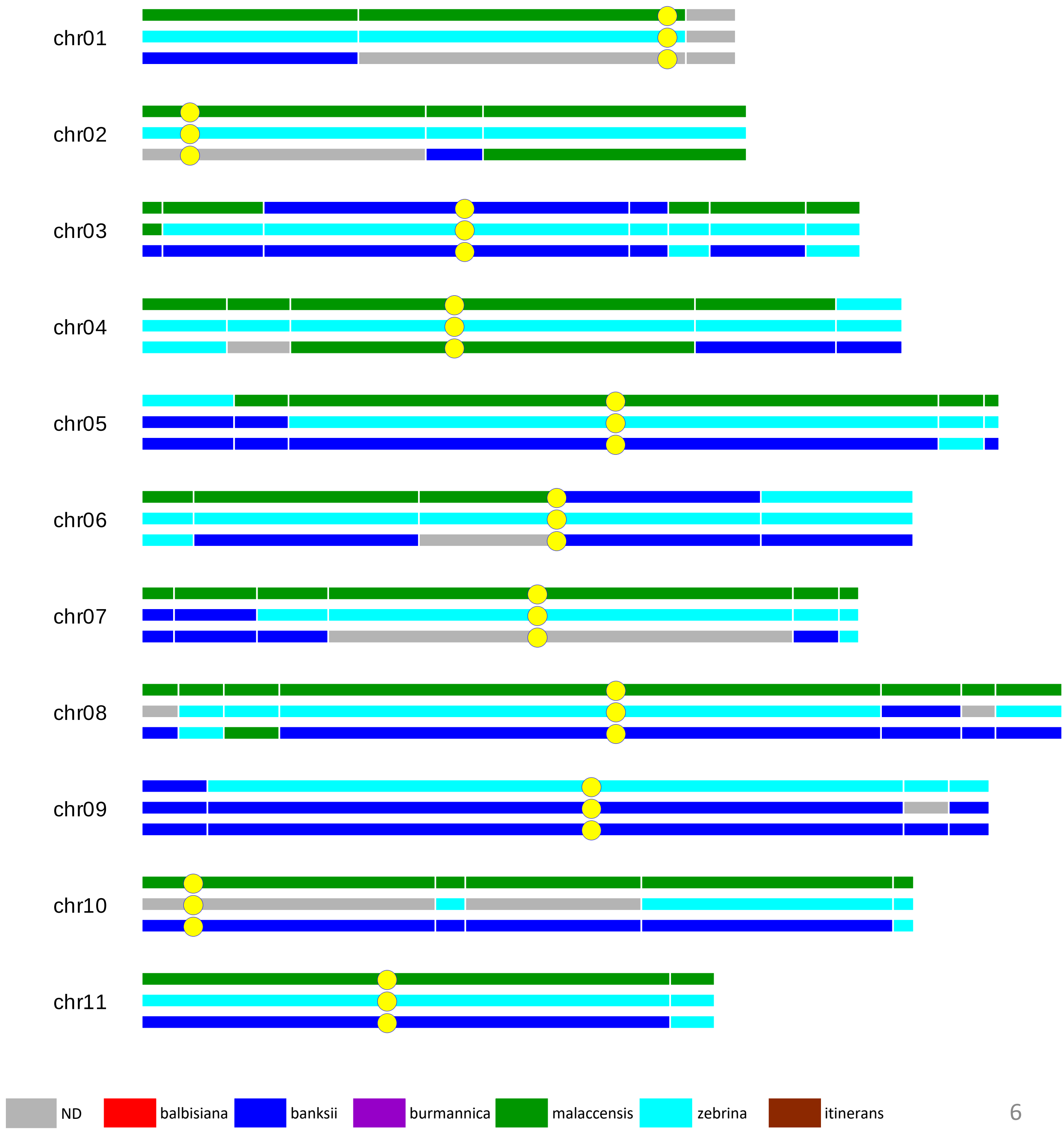

Group: AAA type'269'

- specimen 269, farmer field in Laos
- specimen 305, farmer field in Yunnan (China)

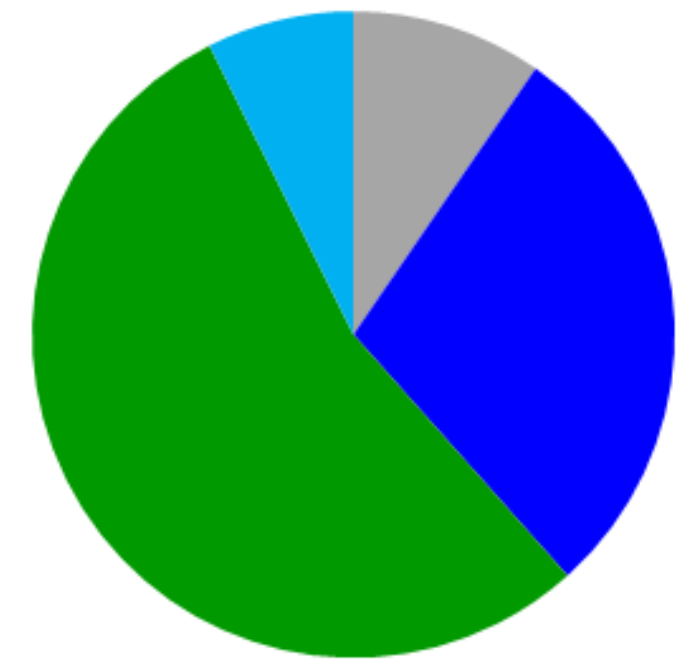

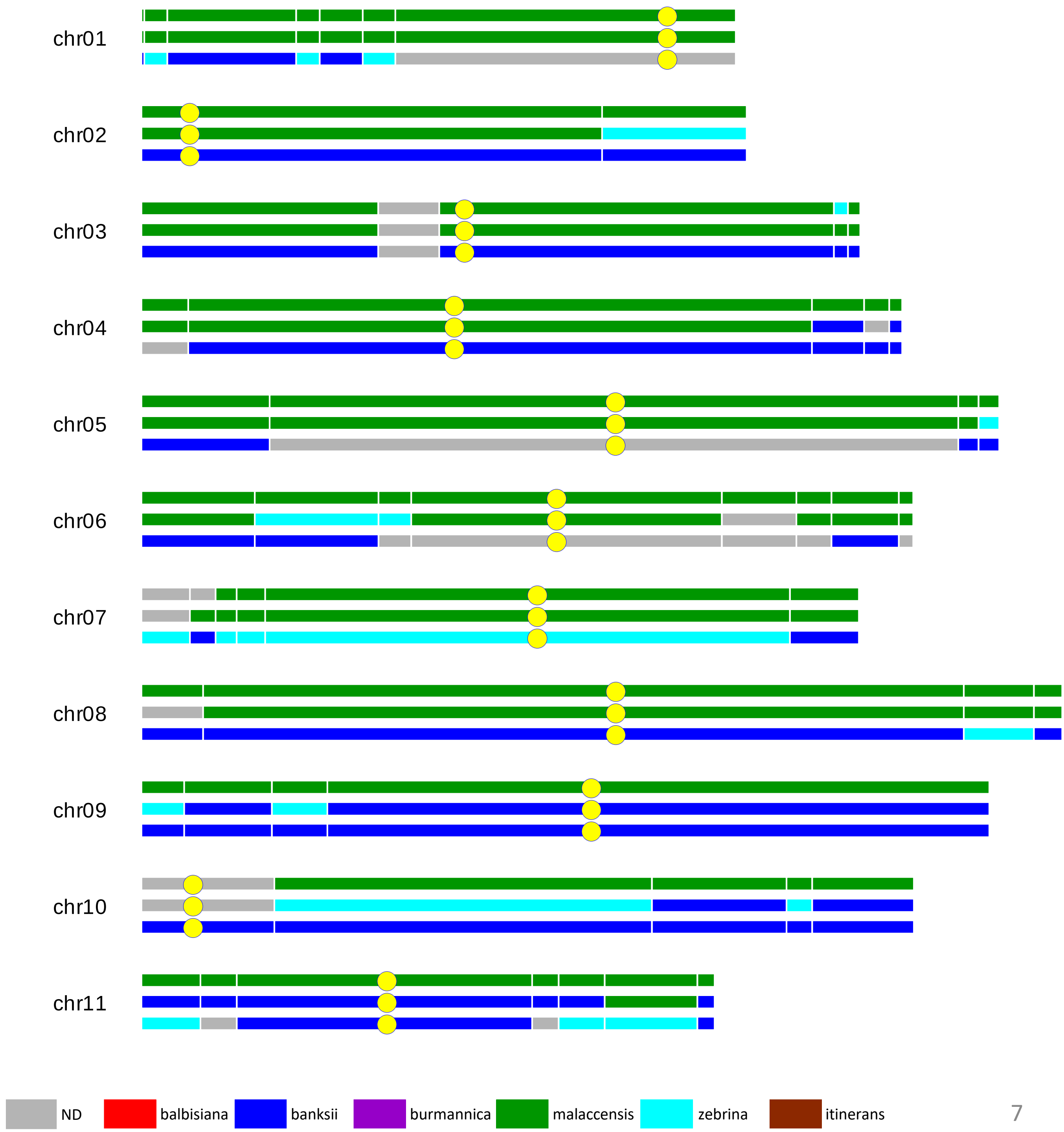

Group: AAA type'108'

- specimen 108, farmer field in Vietnam
- specimens 222 and 228, farmer fields in Laos
- specimen 254, backyard garden in Laos

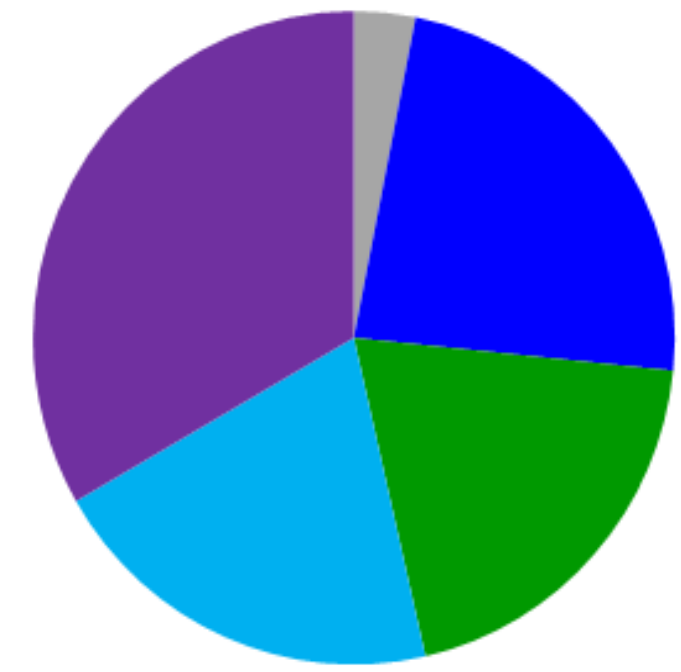

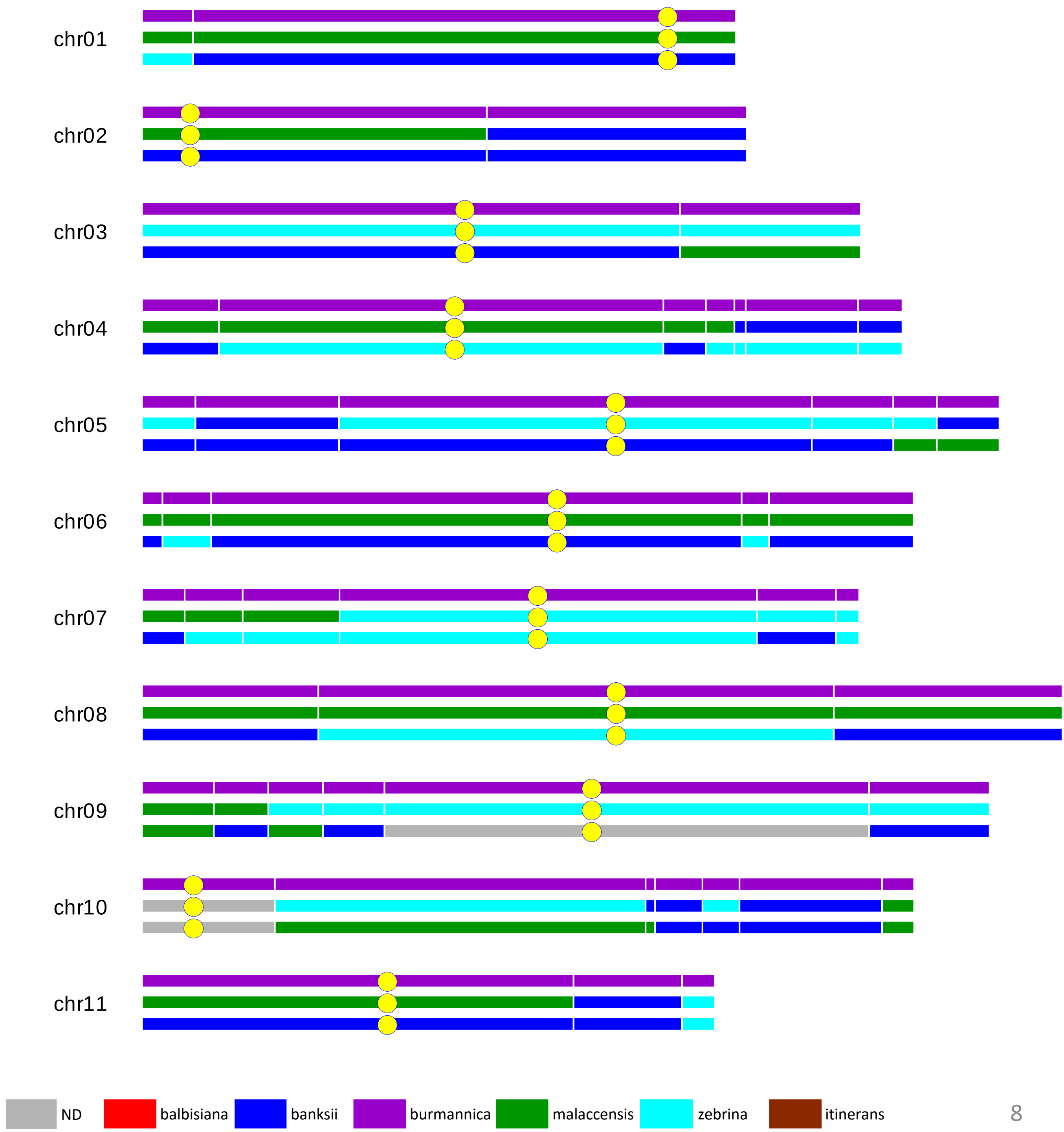

Group: AAA type'33'

- specimen 33, backyard garden in Vietnam
- specimen 270, farmer field in Laos

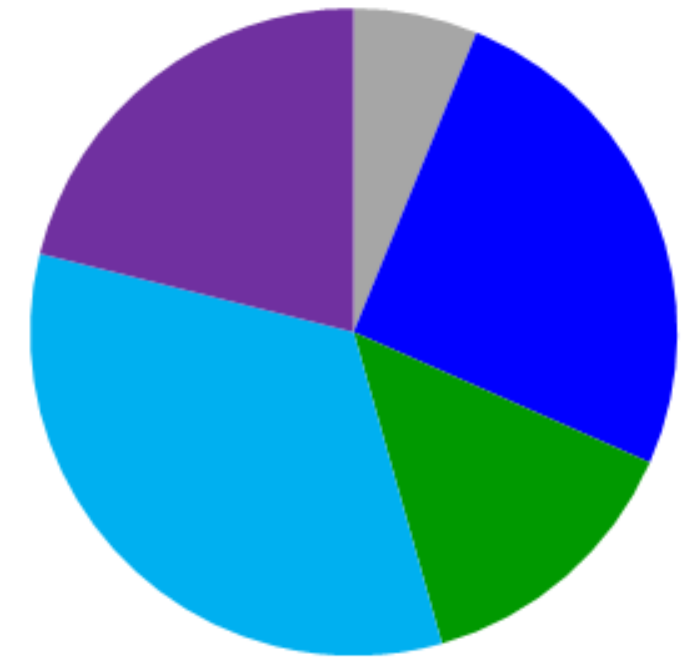

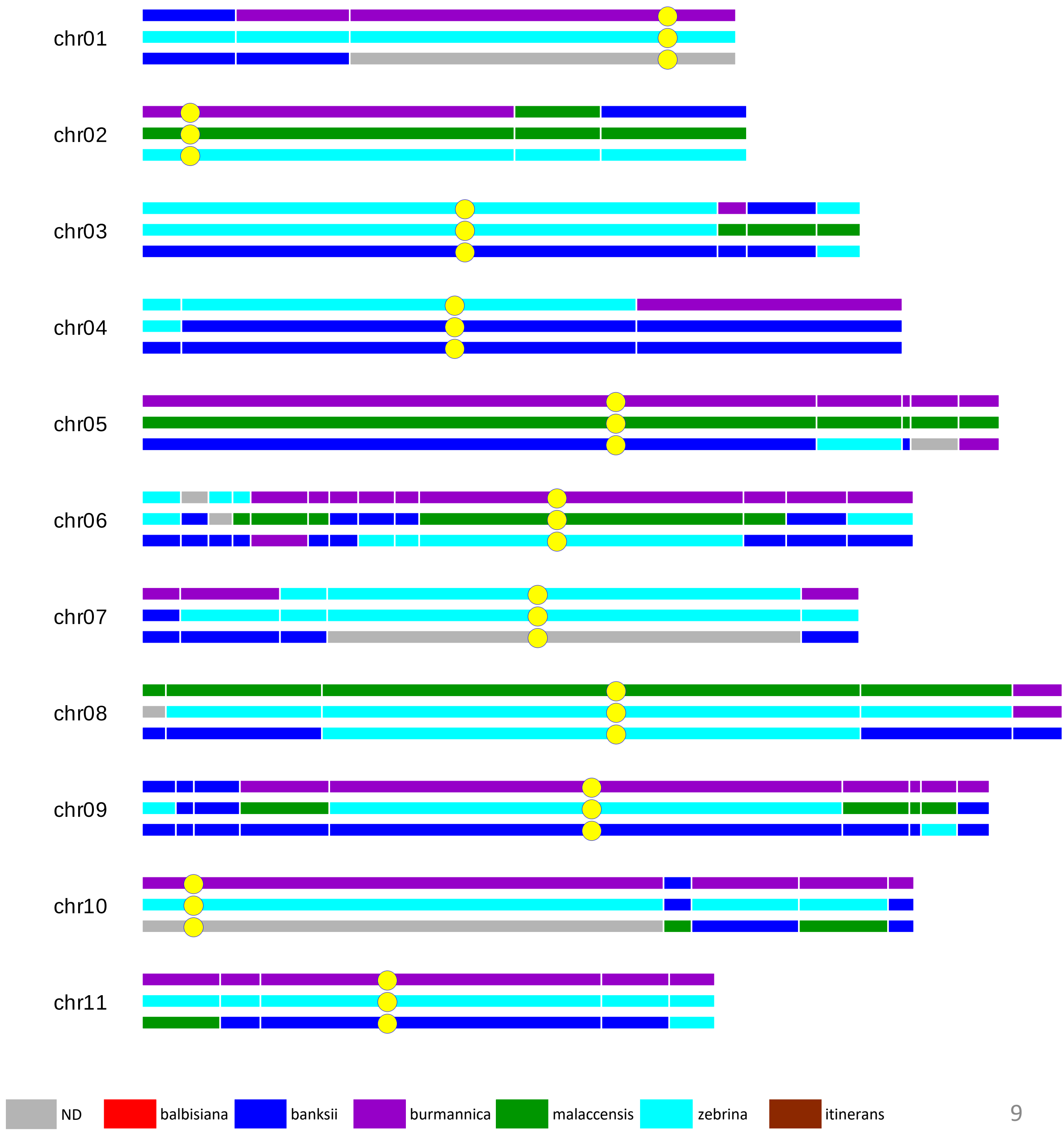

Group: AAB Plantain

- specimen 73 ‘Chuoi Sin Bao’, roadside in Vietnam
- reference 921, ‘Nyombe 2’ from CRBP-PT in Guadeloupe (PT-BA-00248)

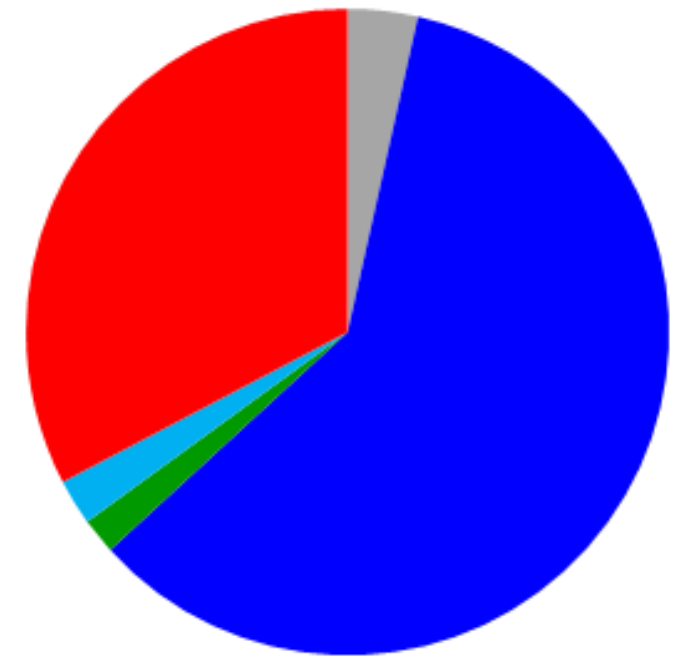

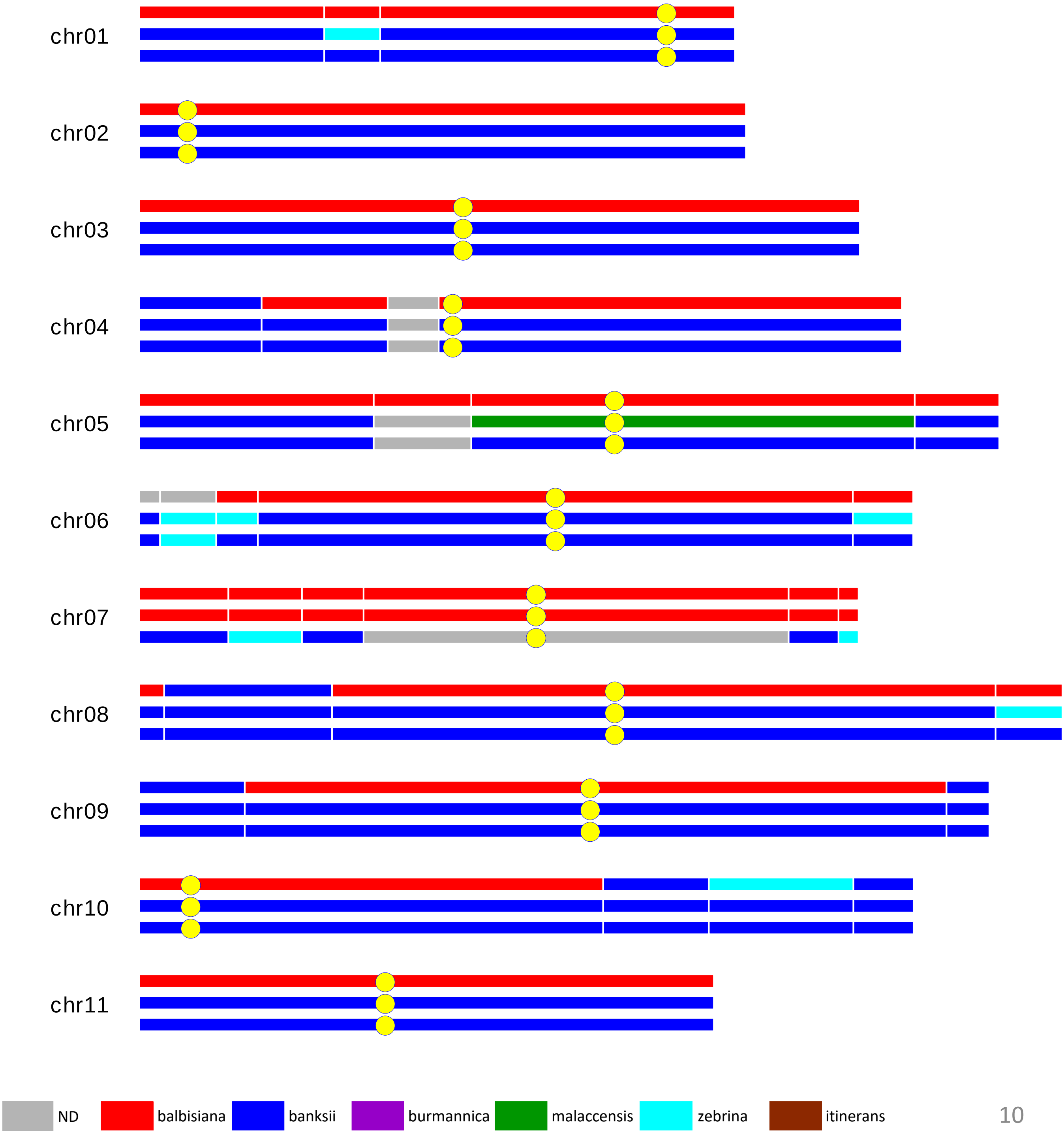

Group: AAB Pome

- specimen 81 ‘Chuoi Mang’, botanical garden in Vietnam
- reference 924, 'Foconah’, from CRBP-PT in Guadeloupe (PT-BA-00091)

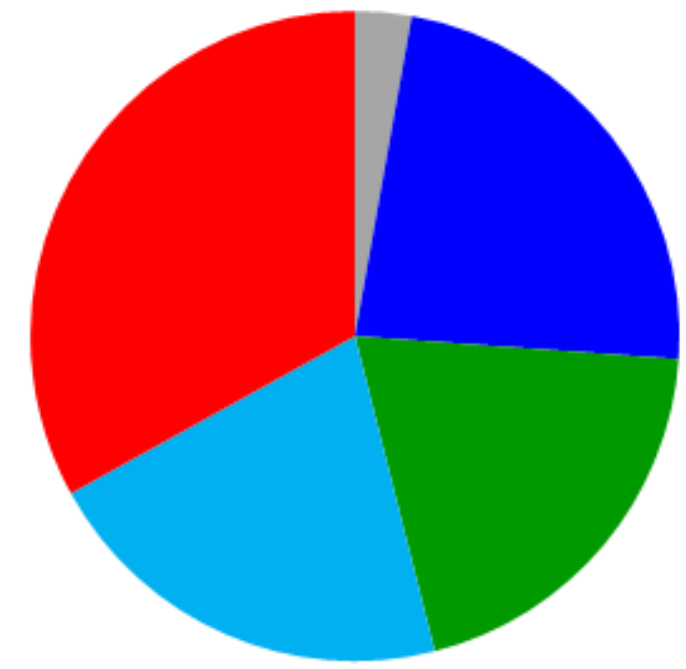

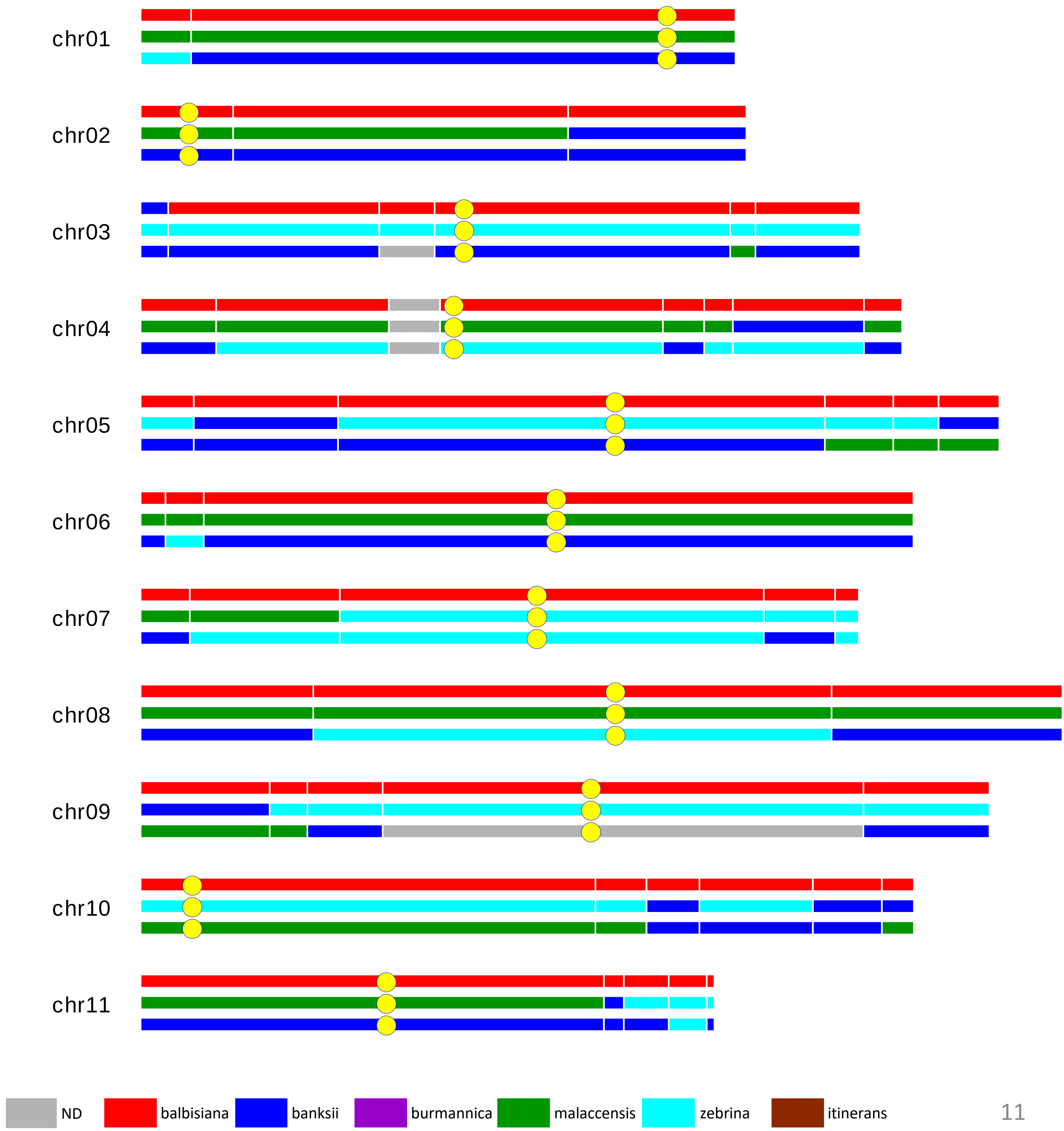

Group: AAB Mysore – Pisang Ceylan

- specimen 18, roadside in Vietnam
- specimens 74 and 75, botanical garden in Vietnam
- reference 920, 'Pisang Ceylan' from CRBP-PT in Guadeloupe (PT-BA-00286)

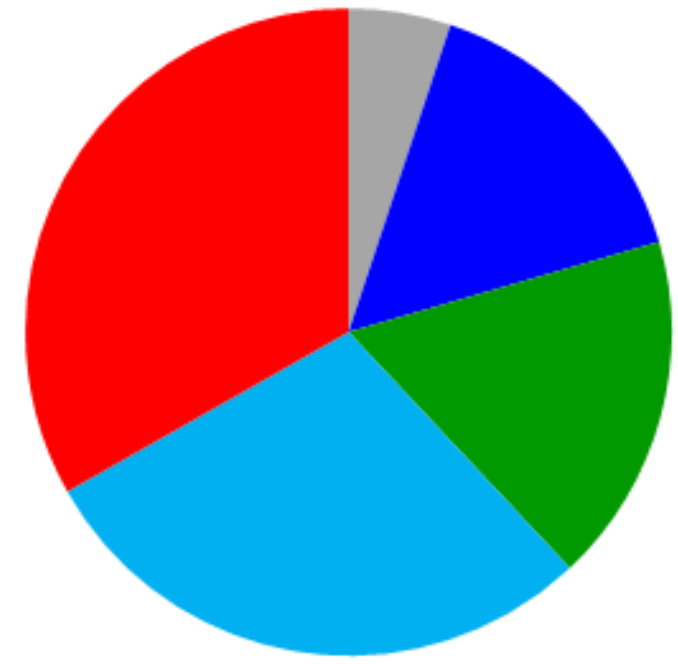

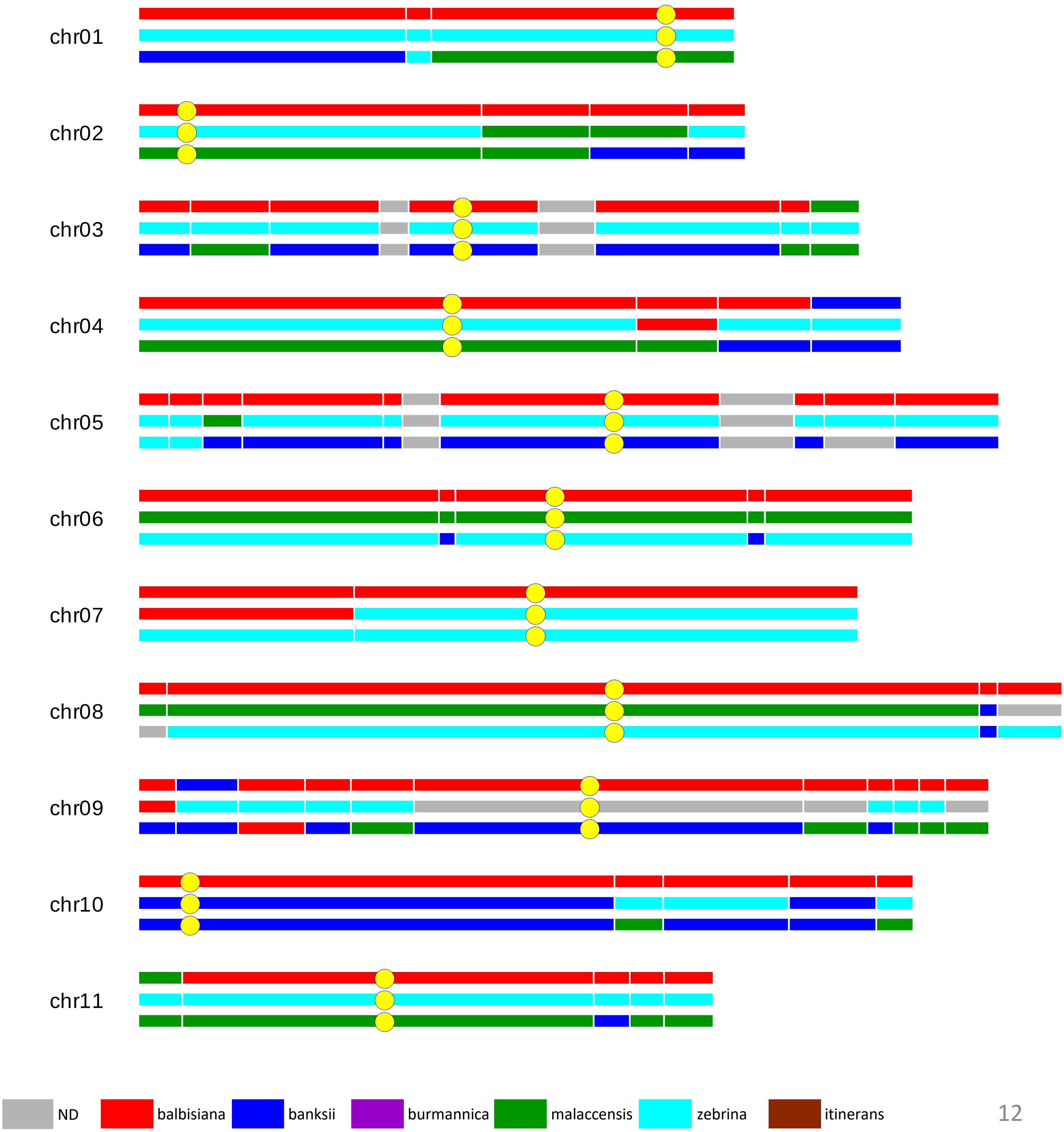

Group: AAB Pisang Kelat / Pisang Seribu

- specimens 80 and 82, botanical garden in Vietnam
- specimen 214, roadside in Laos
- specimen 278, backyard garden in Laos

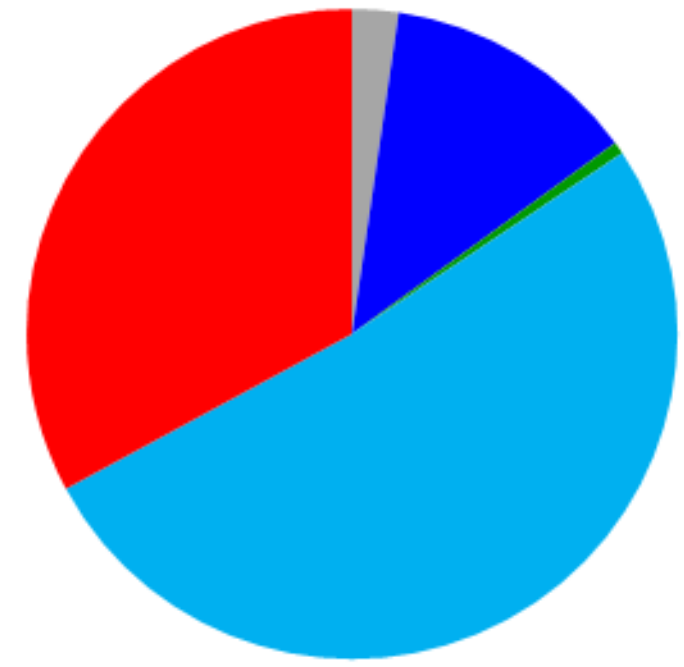

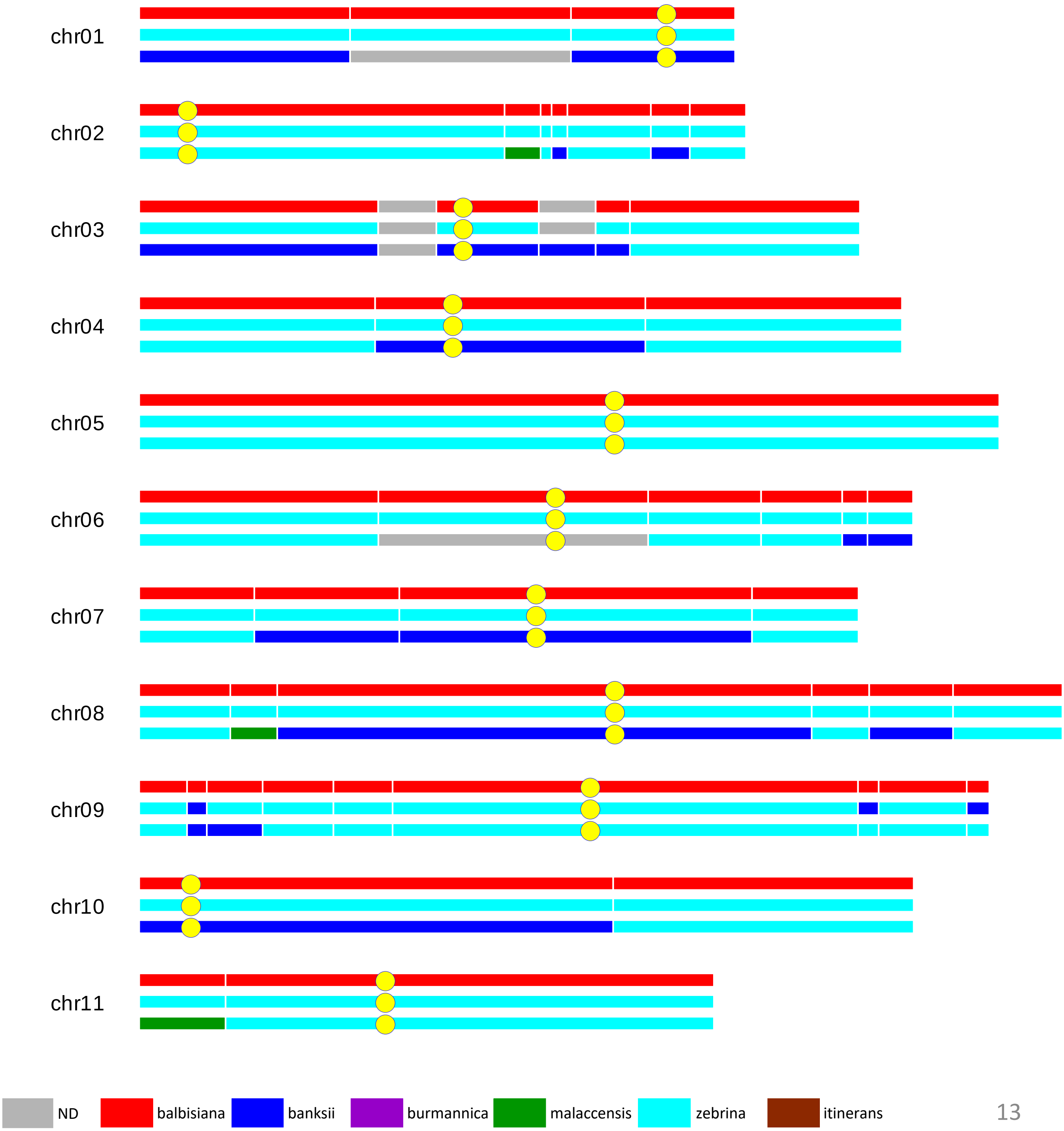

Group: AAB type '139'

- specimen 139, road side in Vietnam
- specimen 331, backyard garden in Yunnan (China)

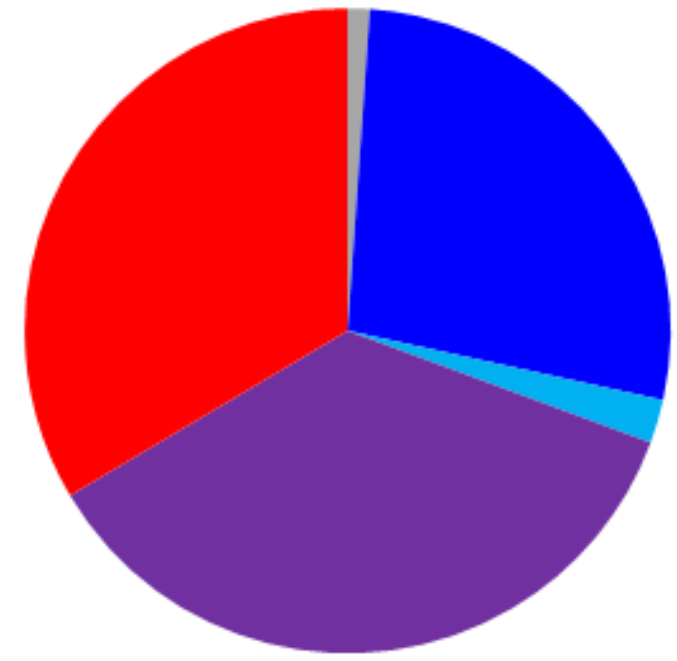

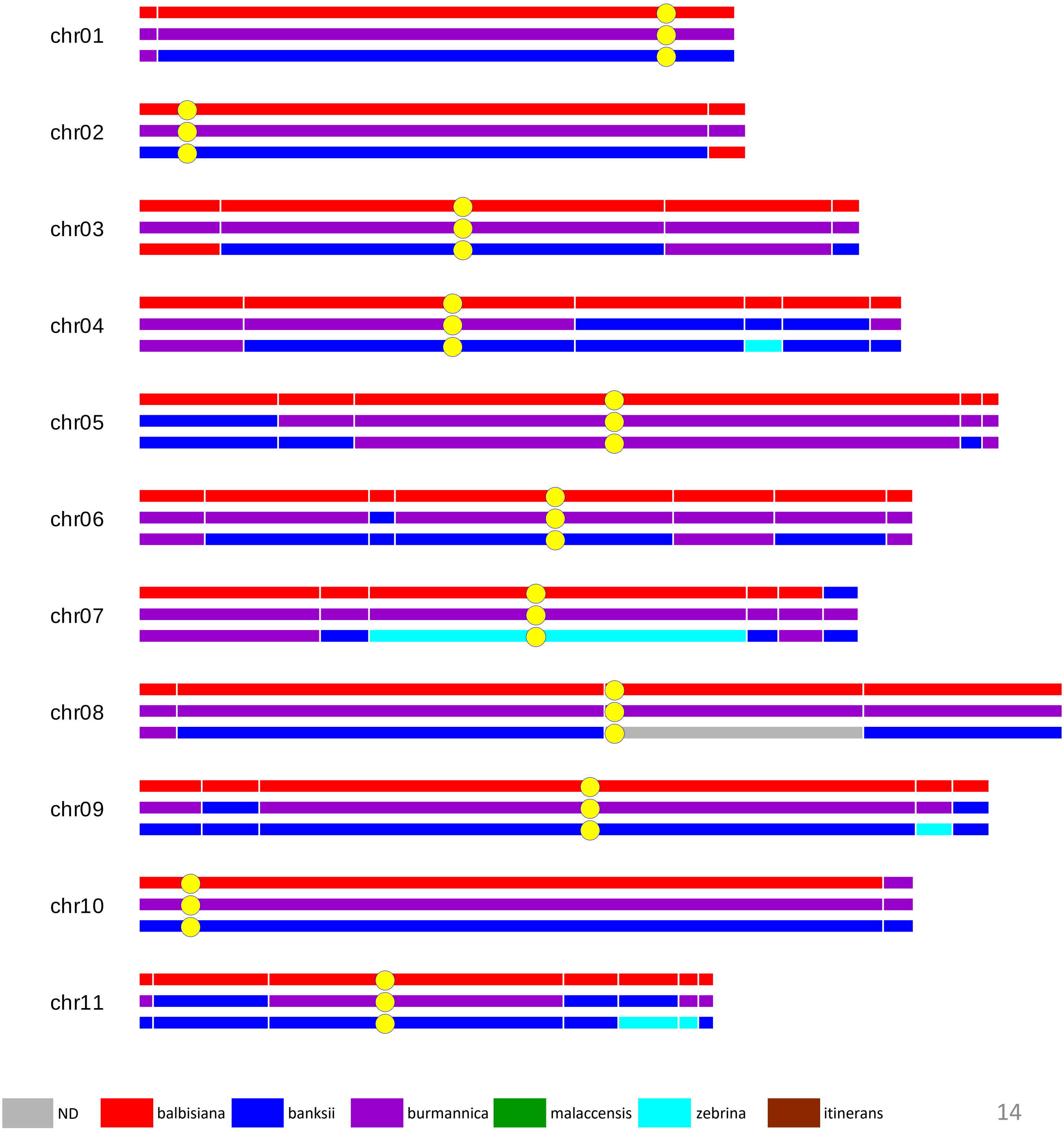

Group: AAB type '156'

- specimen 27, farmer field in Vietnam
- specimen 156, road side in Vietnam
- specimen 357, road side in Yunnan (China)

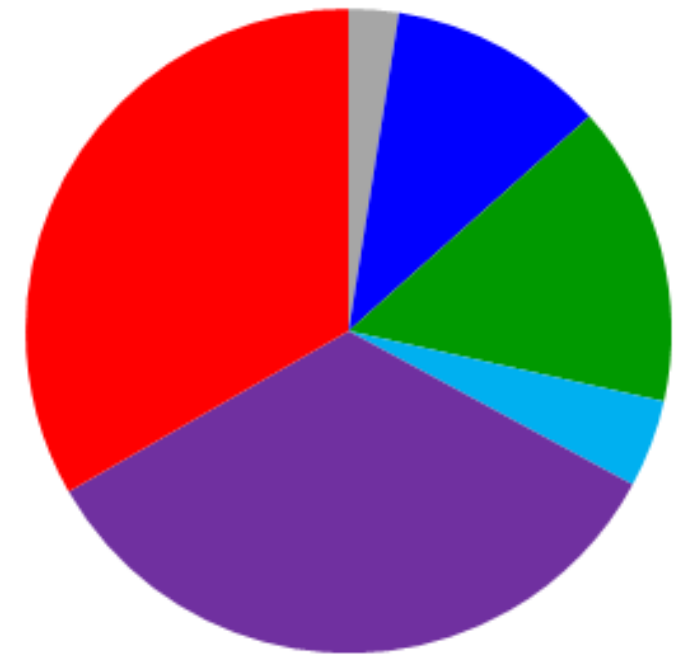

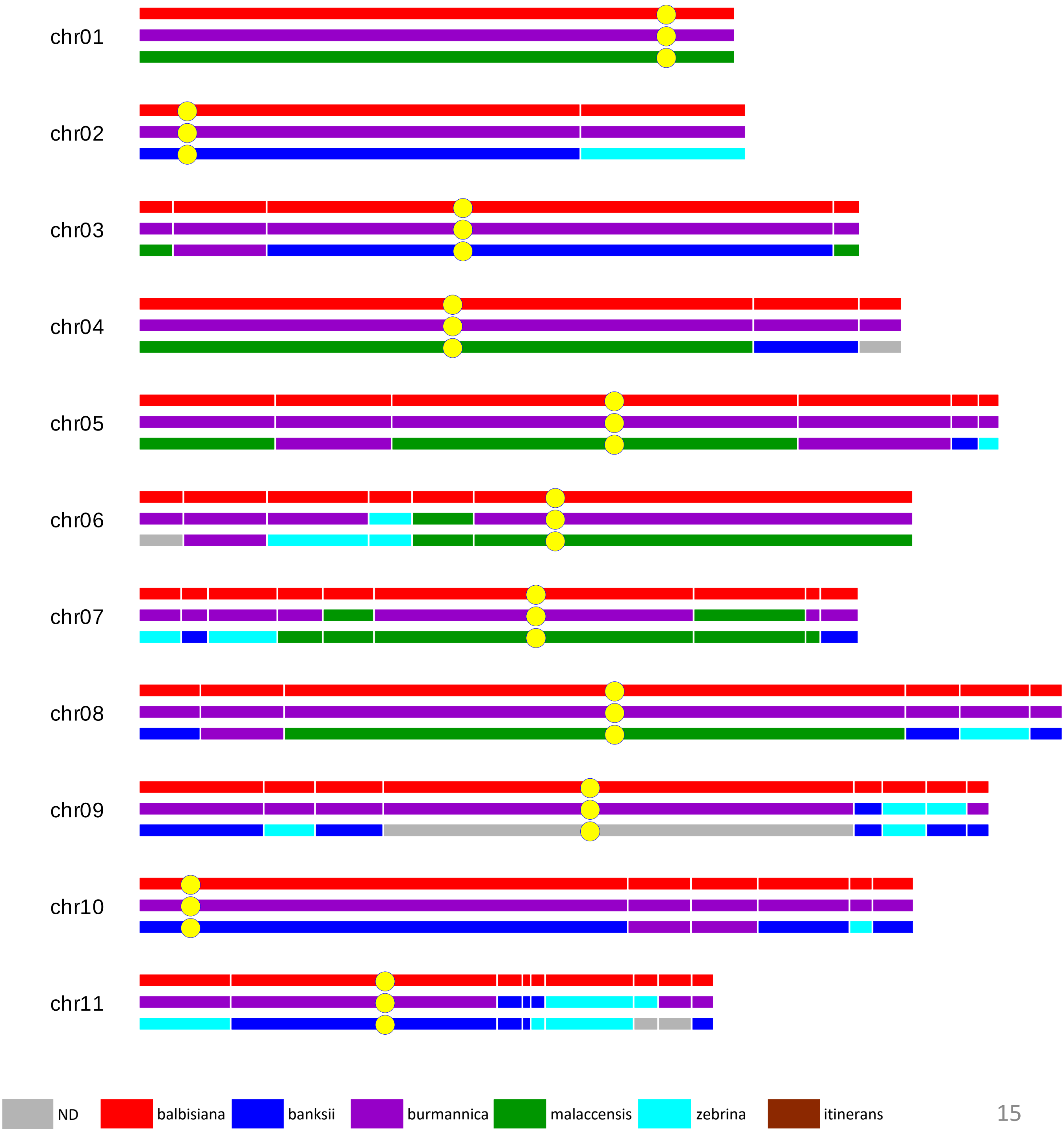

Group: AAB type '268'

- specimen 268, farmer field in Laos

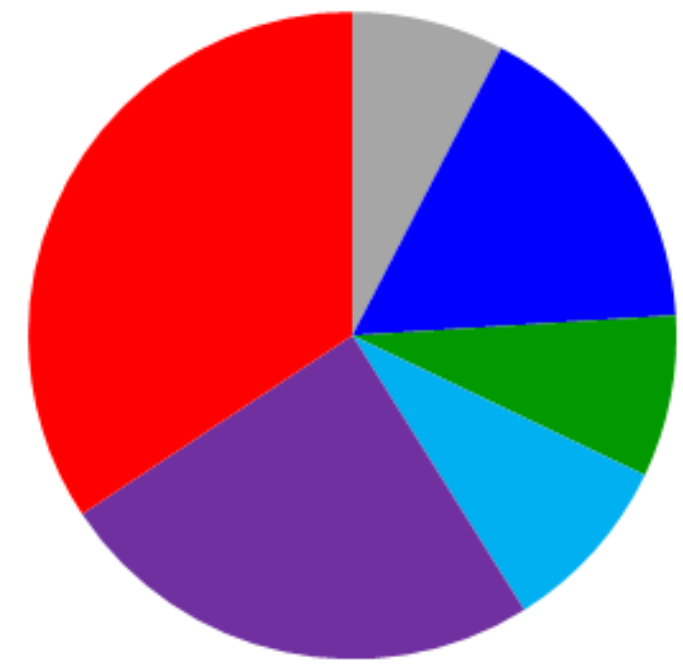

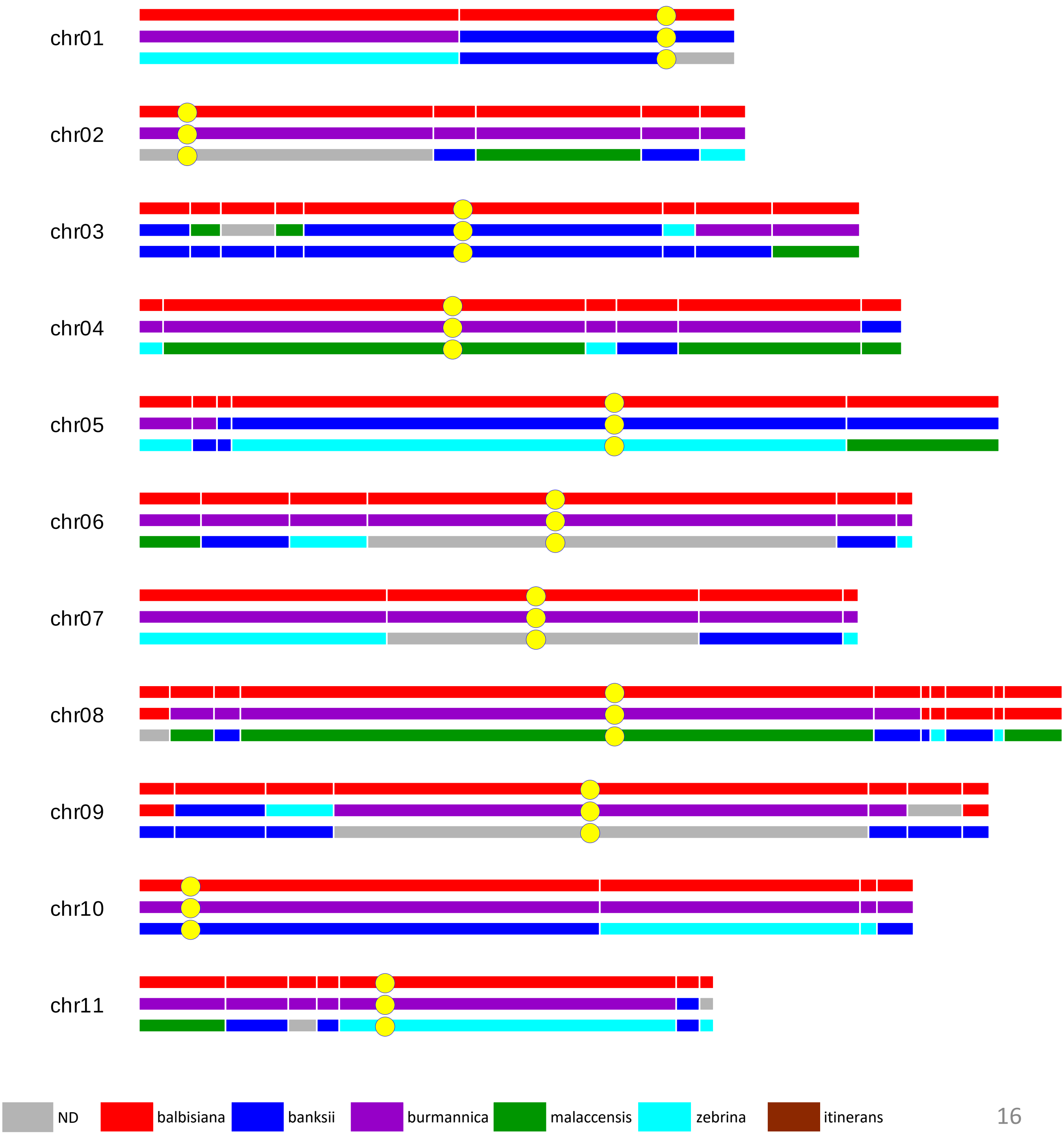

Group: ABB Pisang Awak - type standard

- specimens 11, 19, 42, and 169, road side in Vietnam
- specimens 20, 38, 39, and 50, farmer fields in Vietnam
- specimen 246, backyard garden in Laos
- specimen 283, farmer field in Laos
- reference 926, 'Namwa Khom' from CRBP-PT in Guadeloupe (PT-BA-00234)

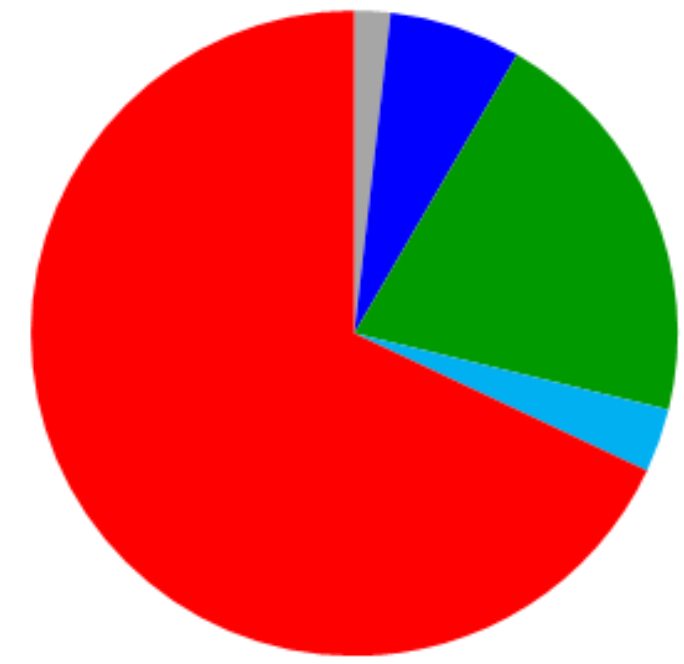

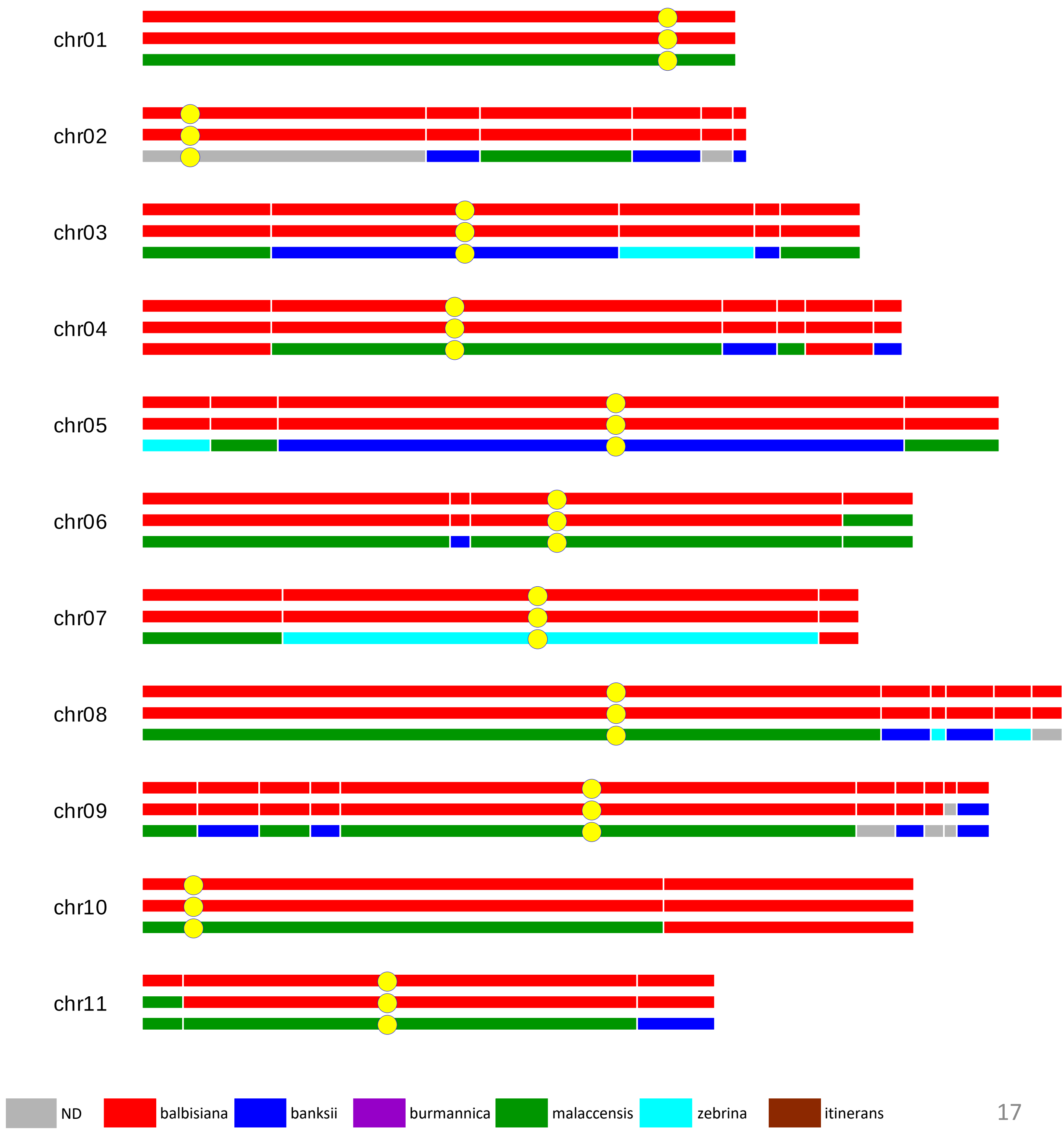

Group: ABB Pisang Awak variant ‘343’

- specimen 343, backyard garden in Yunnan (China)

(in grey dotted boxes, variations from the standard type)

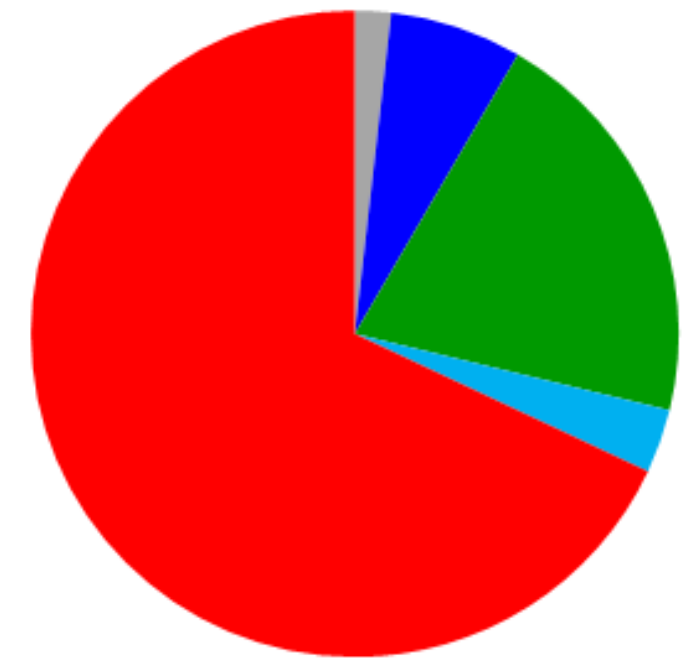

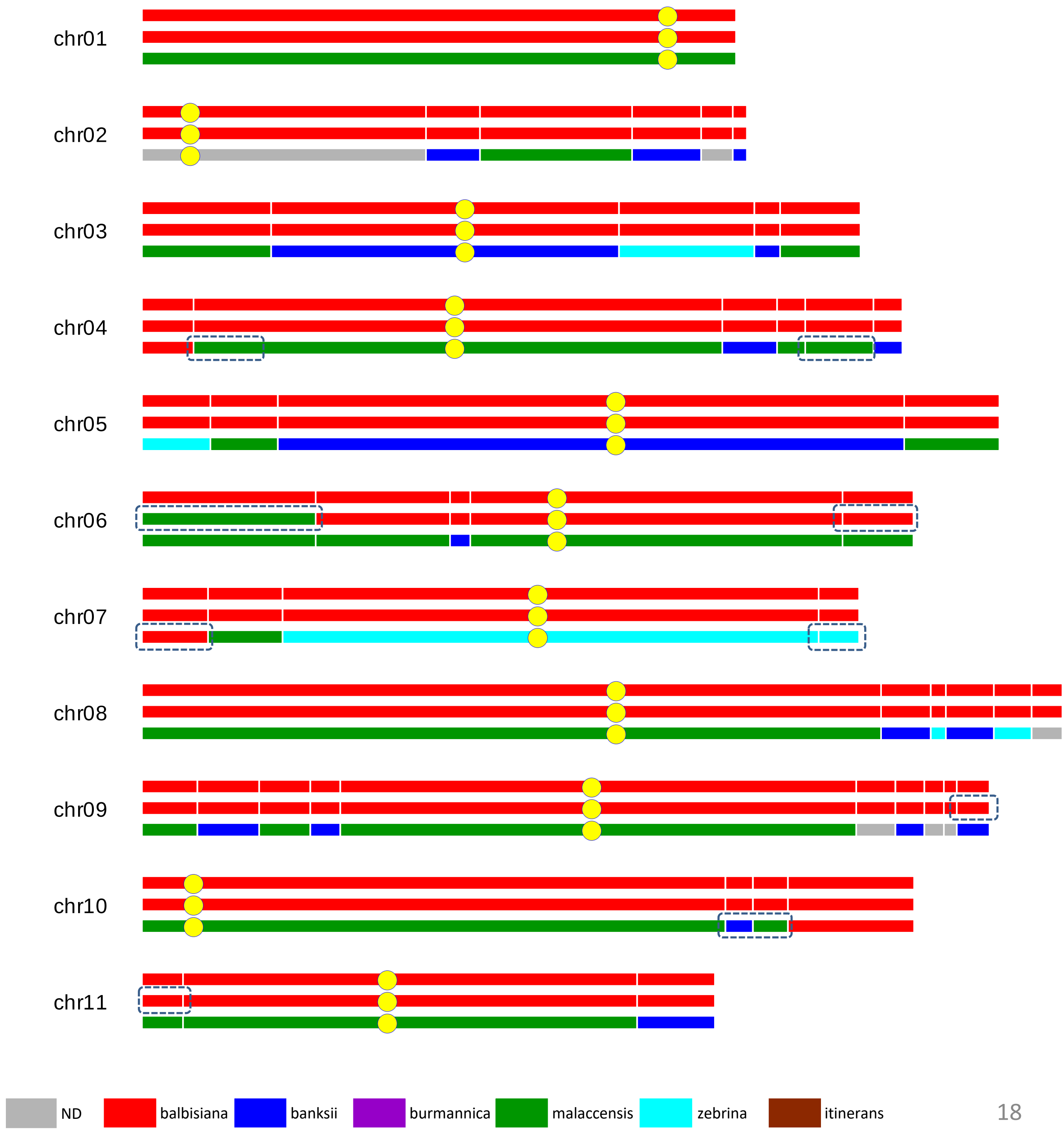

Group: ABBB Pisang awak tetraploid

- specimen 91, road side in Vietnam

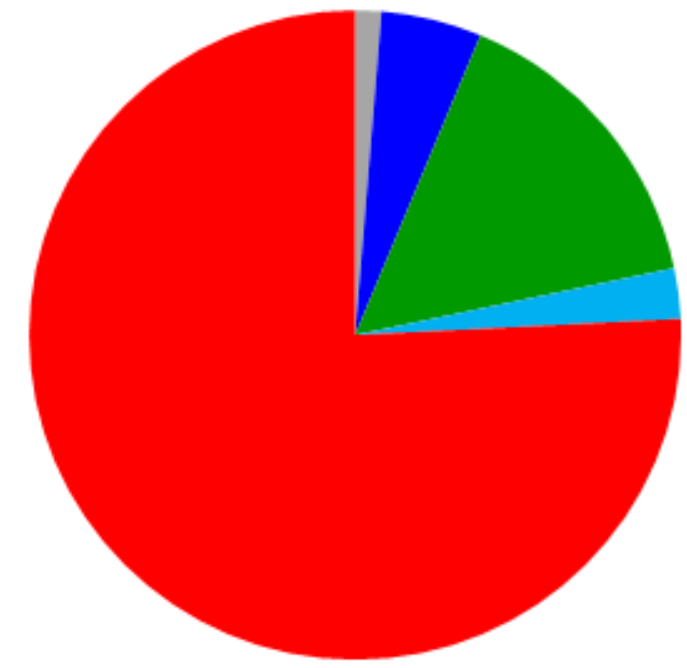

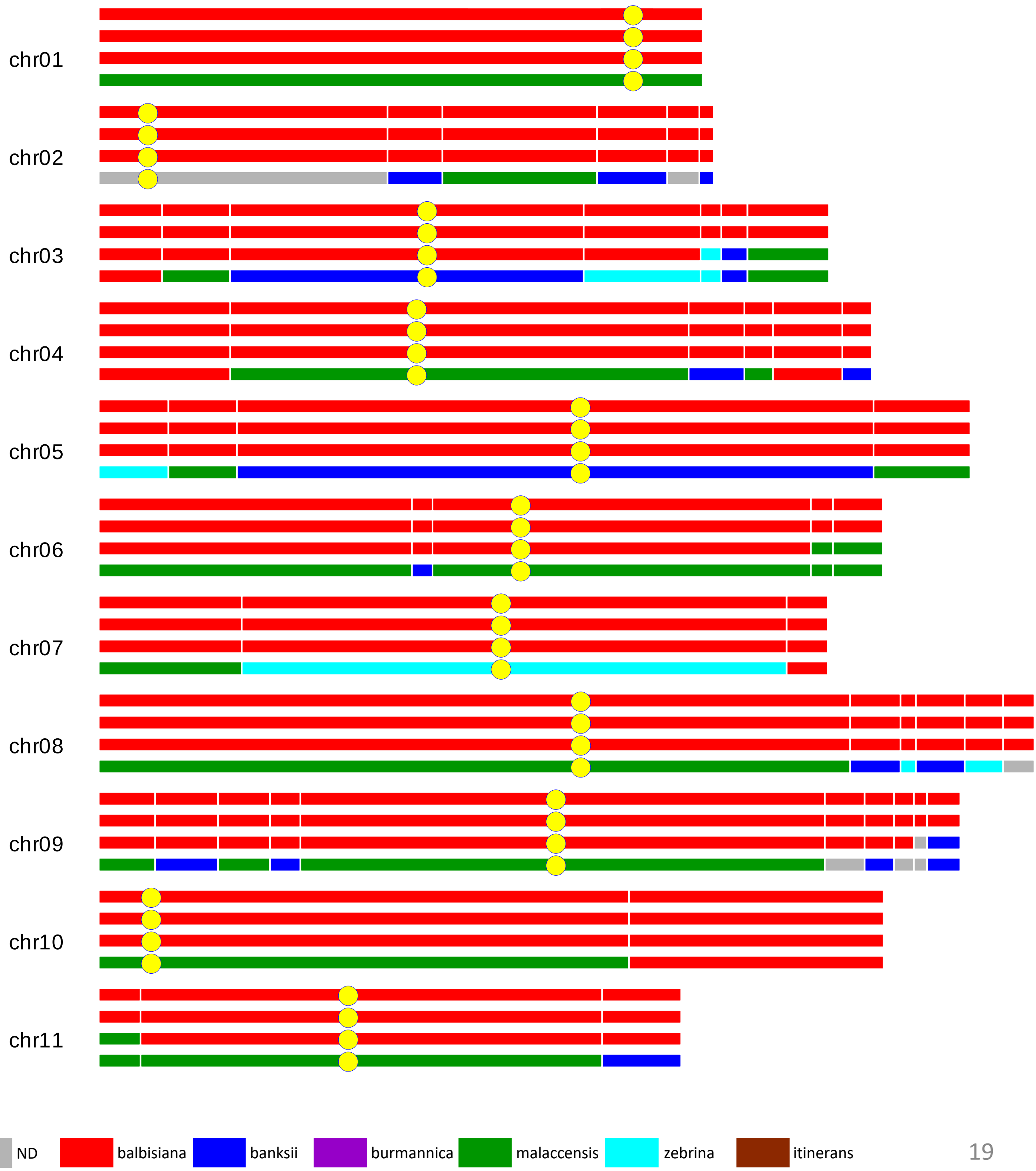

Group: ABB Bluggoe

- reference 925, 'Dole' from CRBP-PT in Guadeloupe (PT-BA-00073)

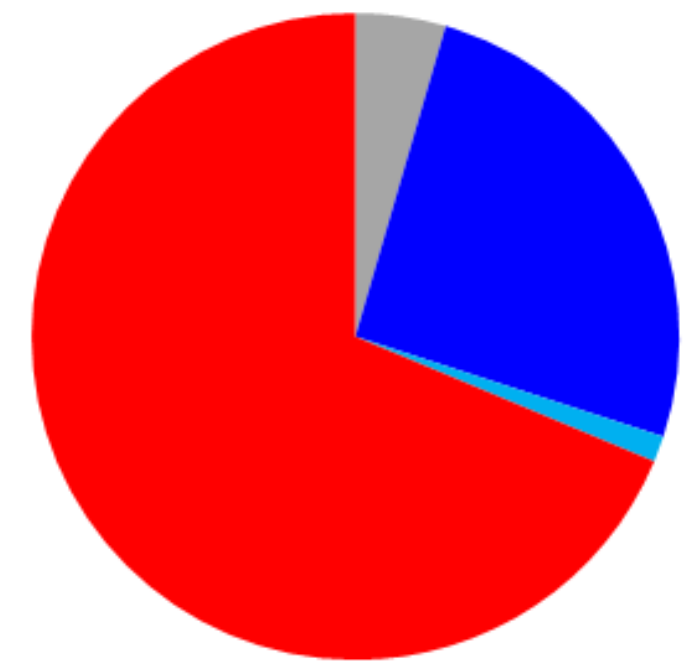

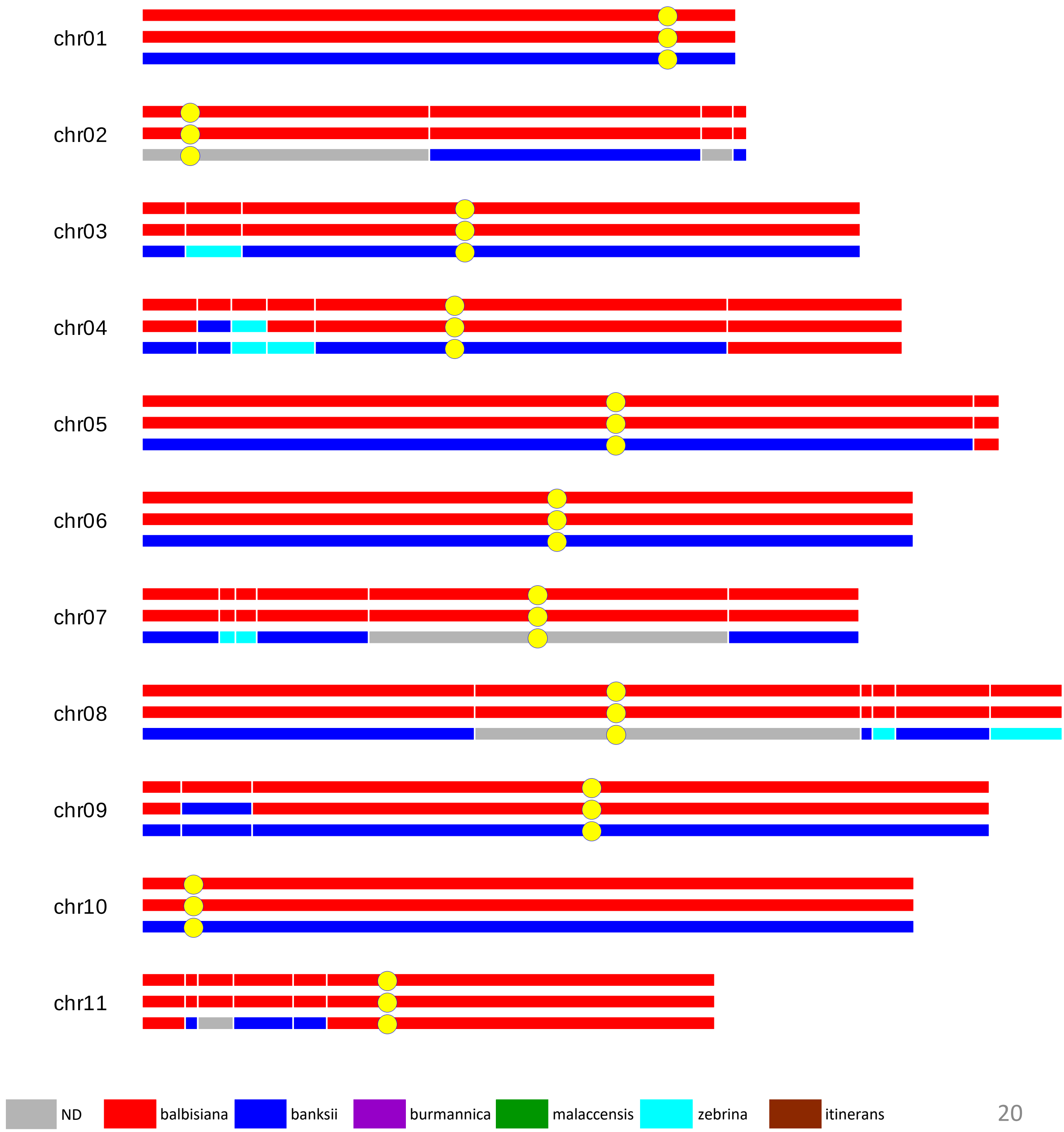

Group: ABB Bluggoe variant ’79’

- specimen 79, botanical garden in Vietnam

(in grey dotted boxes, variations from the reference ‘Dole’)

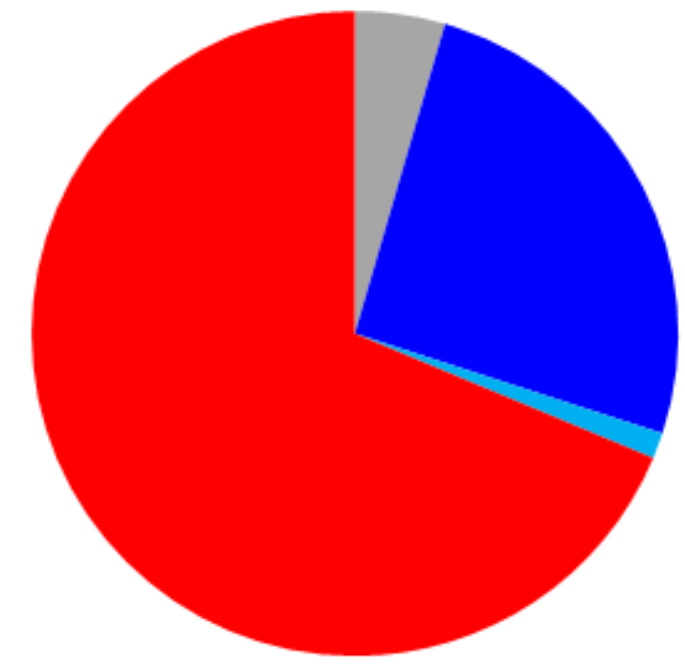

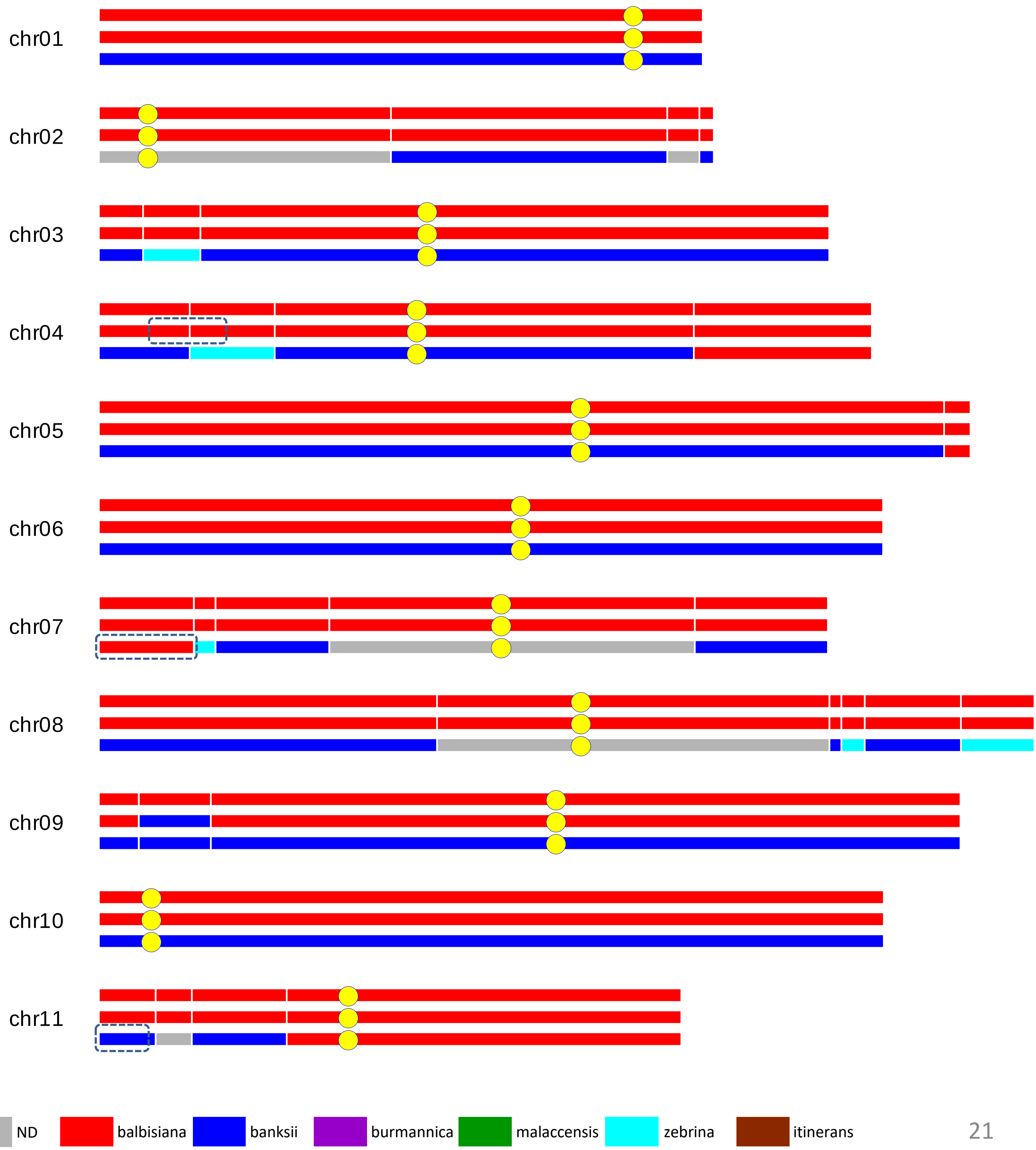

Group: ABB Bluggoe variant ‘149’

- specimen 149, farmer field in Vietnam
- specimen 265, backyard garden in Laos

(in grey dotted boxes, variations from the reference ‘Dole’)

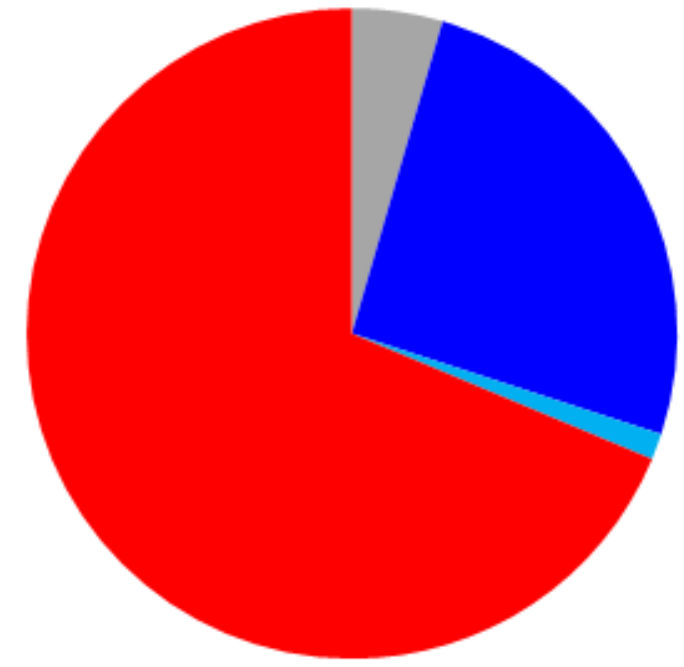

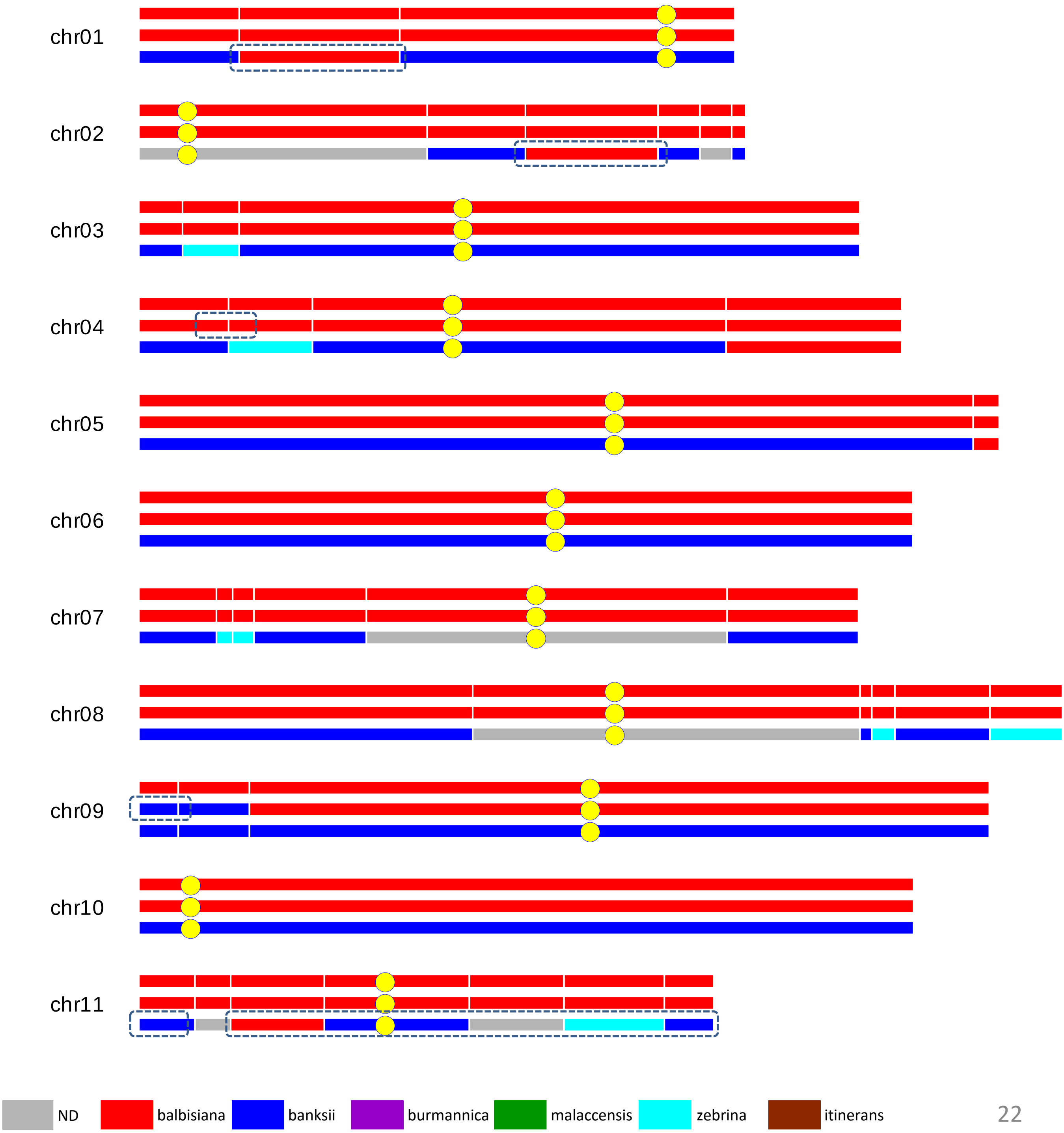

Group: ABB Bluggoe variant ‘154’

- specimen 154, road side in Vietnam

(in grey dotted boxes, variations from the reference ‘Dole’)

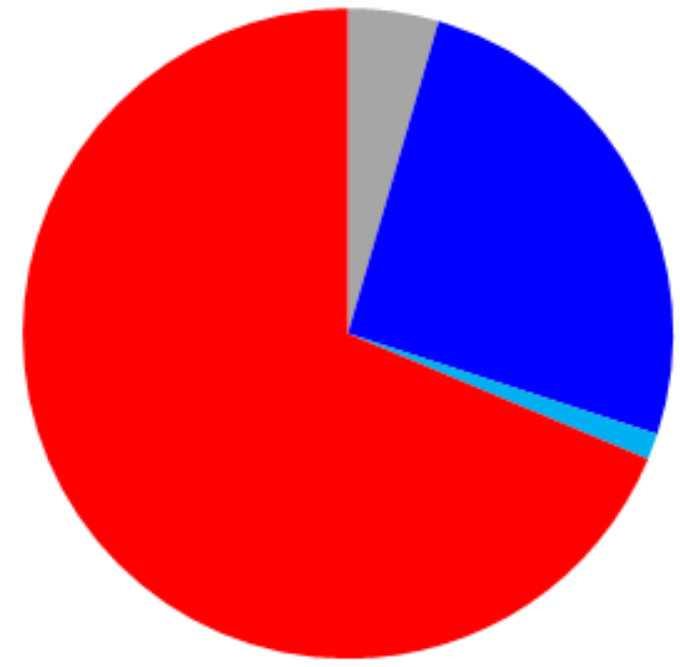

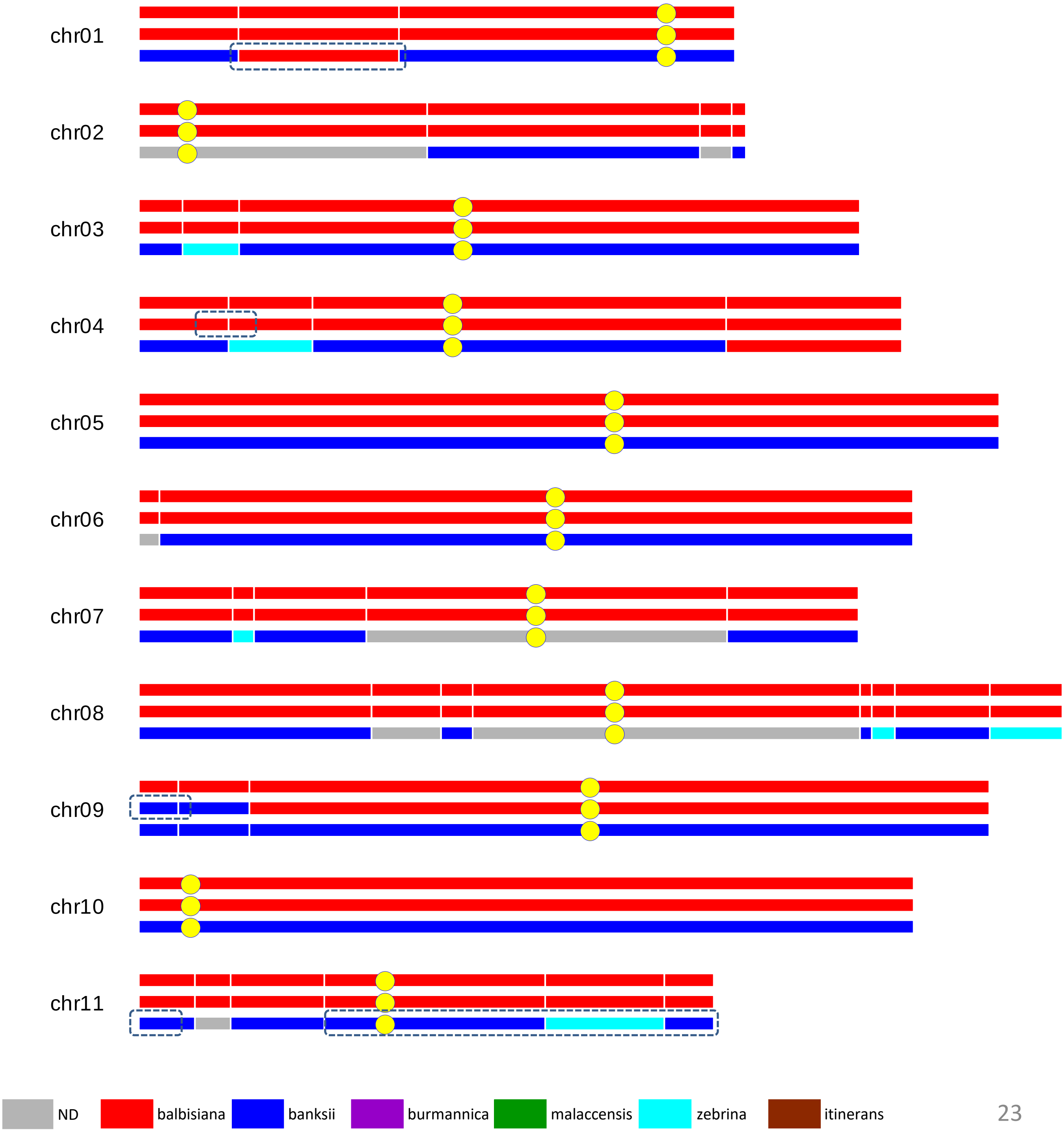

Group: ABB Klue Tiparot - type standard

- specimen 135, farmer field in Vietnam
- specimen 258, roadside in Laos
- reference 930, from CRBP-PT in Guadeloupe (PT-BA-00156)

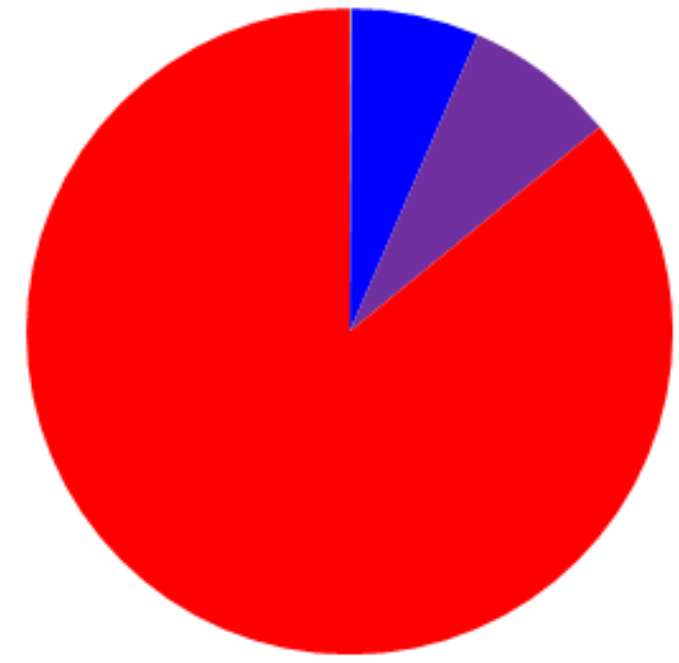

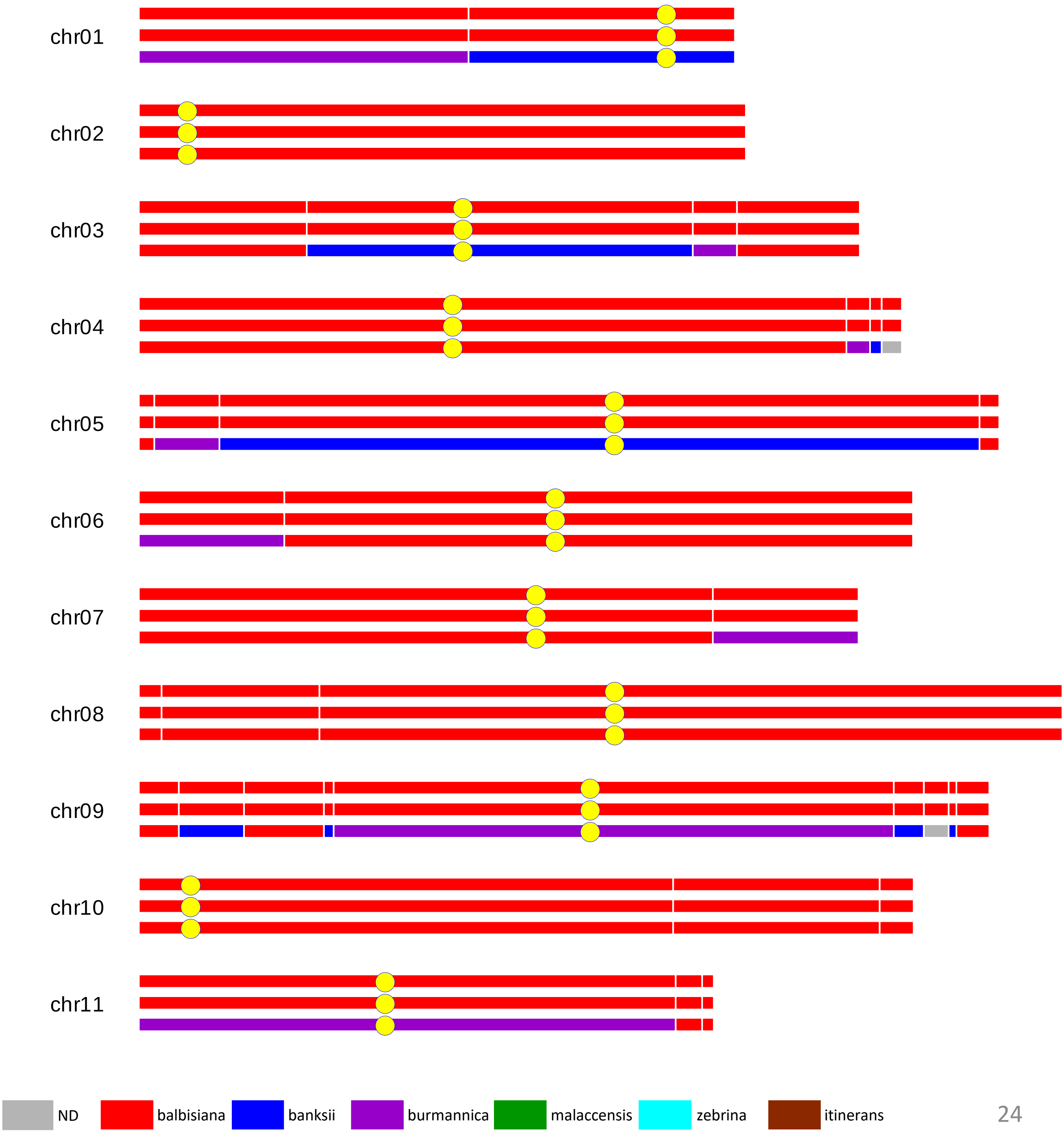

Group: ABB Klue Tiparot variant '224'

- specimen 224, roadside in Laos

(in grey dotted boxes, variations from the standard type)

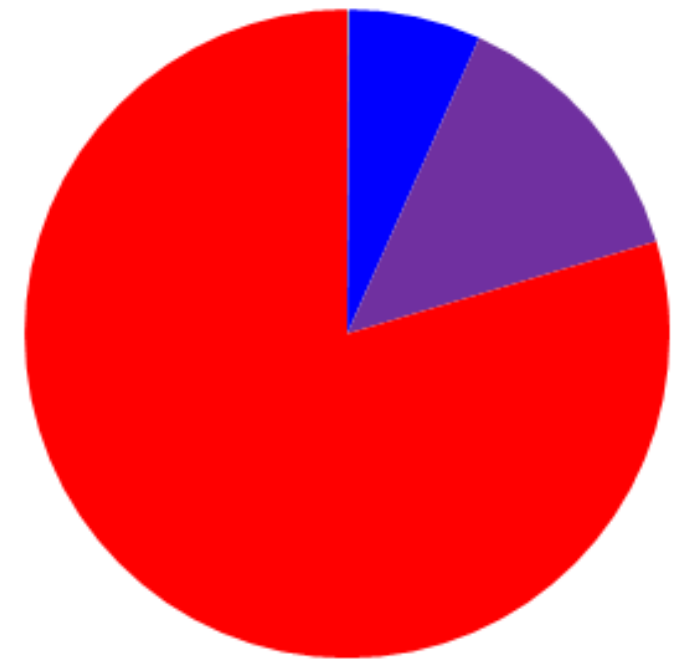

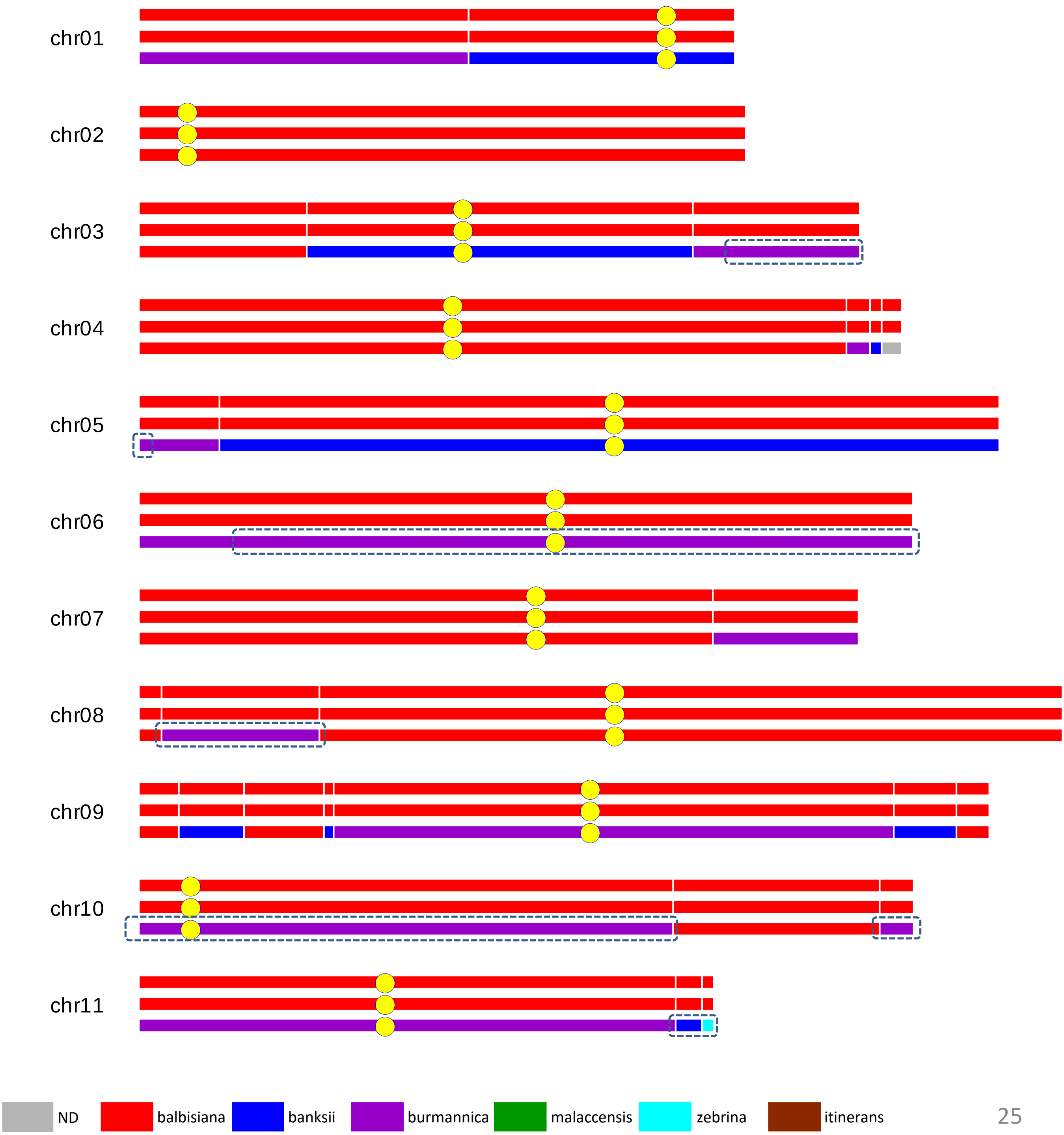

Group: ABI type ‘28’

- specimens 28, 32 ‘Chuoi Ngop’
- specimen 120, road side in Vietnam
- specimen 219 ‘Kuay Som’, backyard garden in Laos

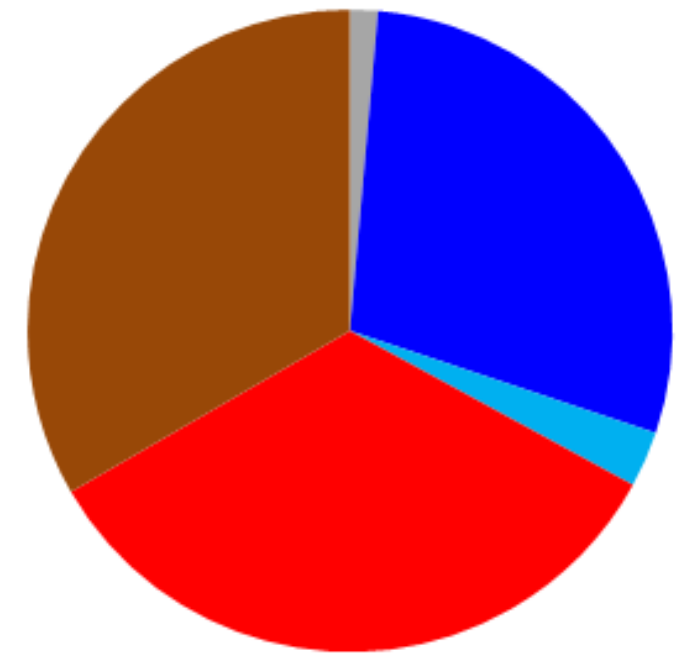

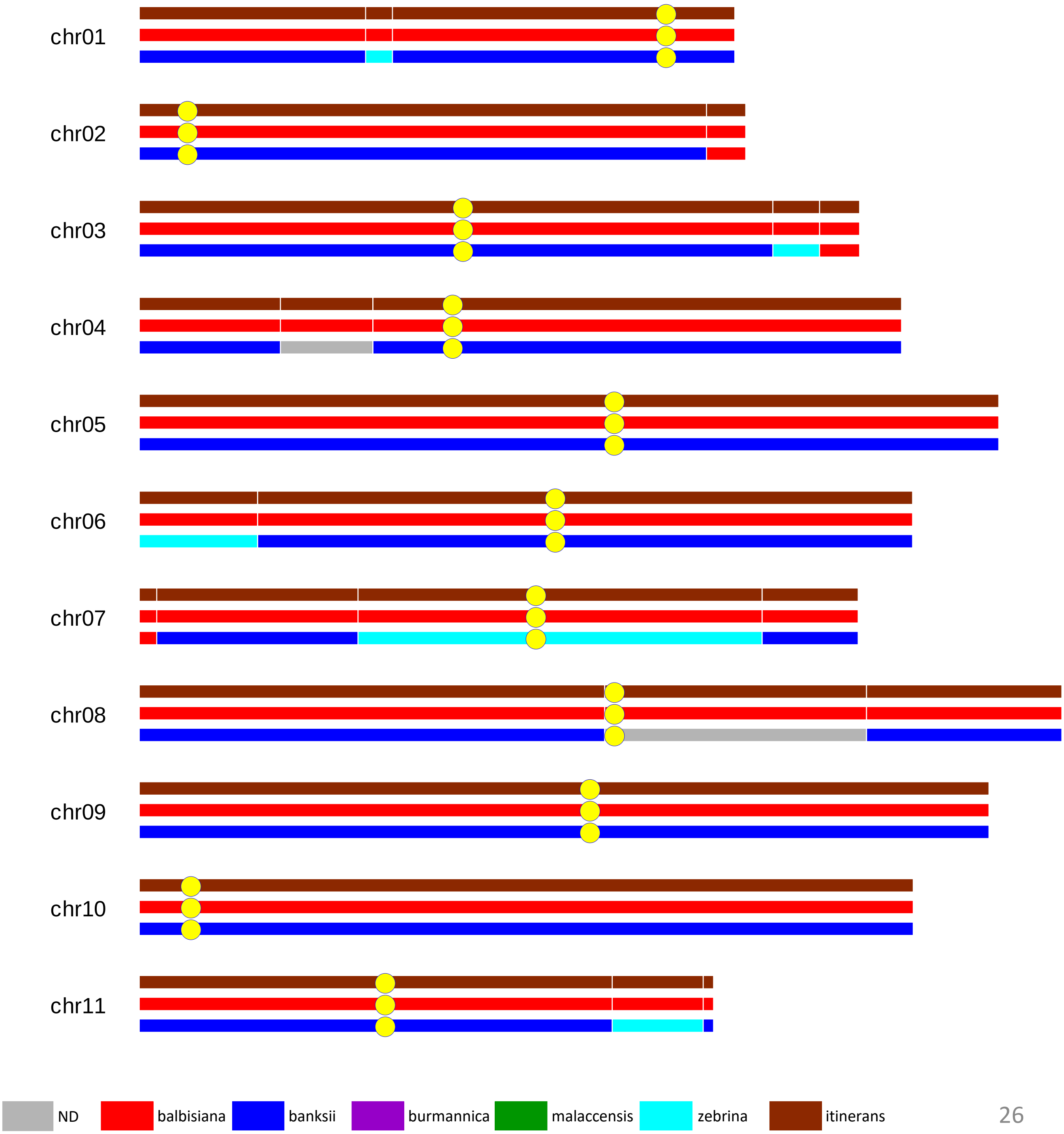

Group: ABI type ‘28’ variant ‘134’

- specimen 134 , farmer field in Vietnam

(in grey dotted boxes, variation from type ‘28’)

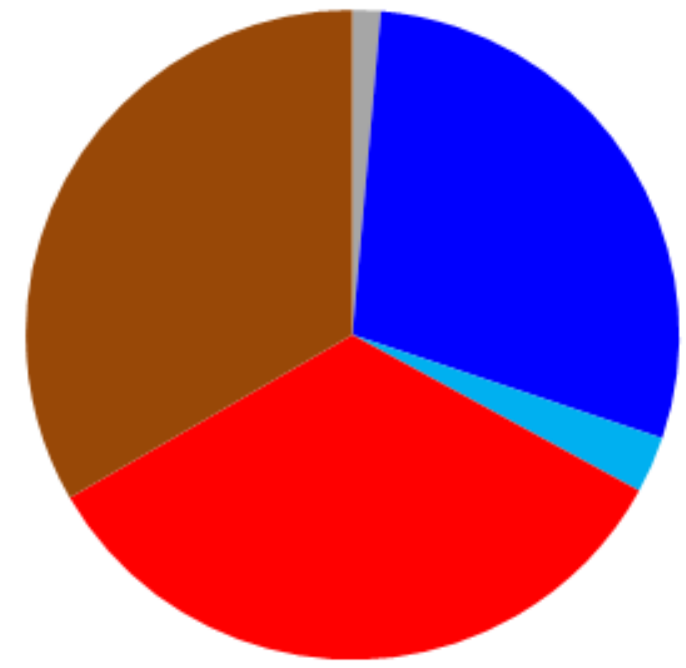

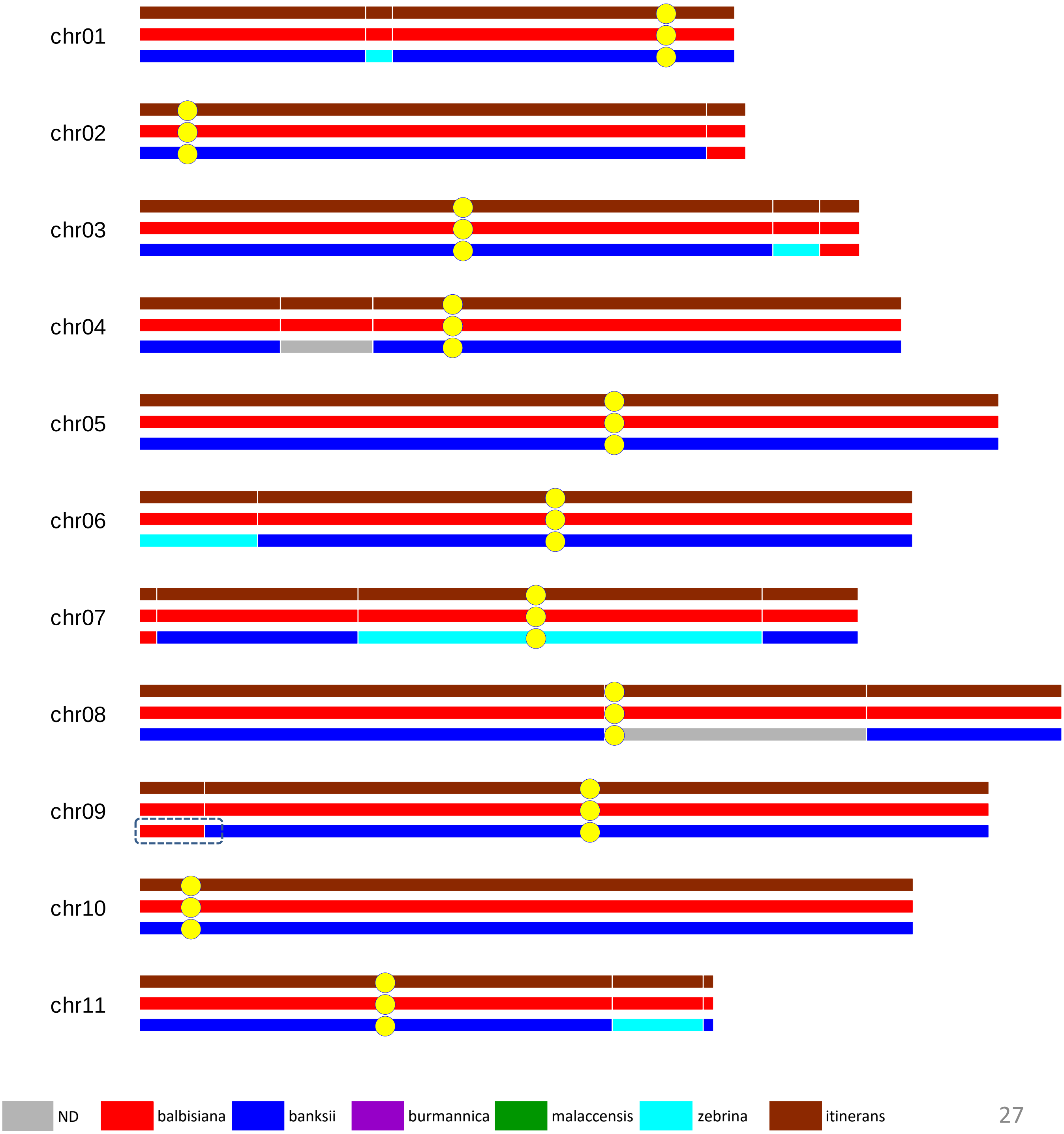

Group: ABI type '223'

- specimen 223, farmer field in Laos

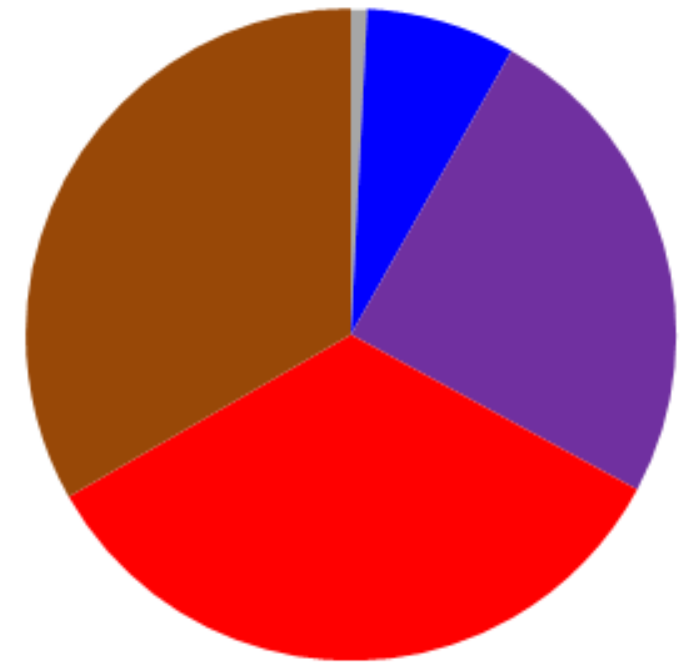

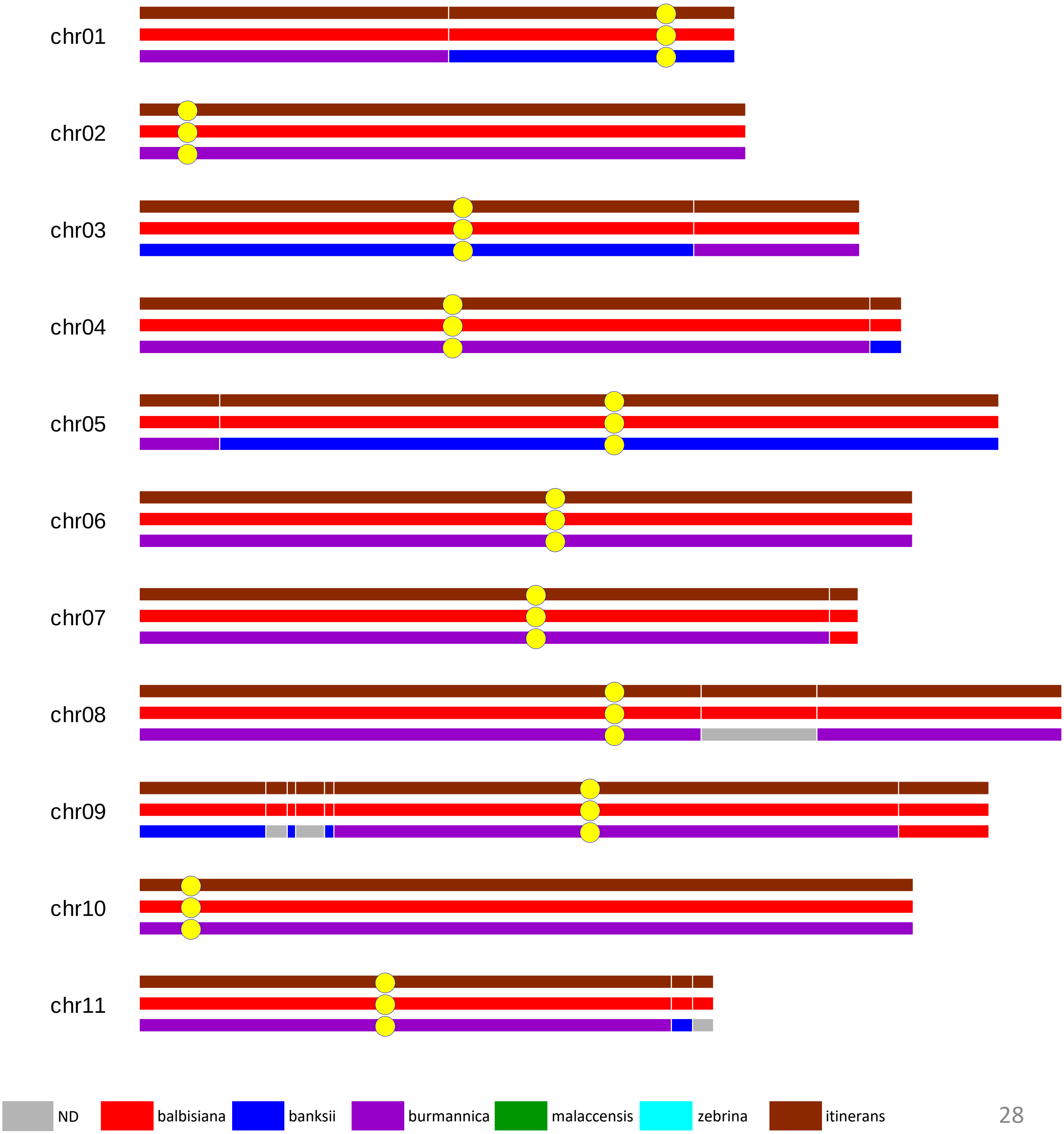

Group: ABBI type ’77’

- specimen 77, botanical garden in Vietnam

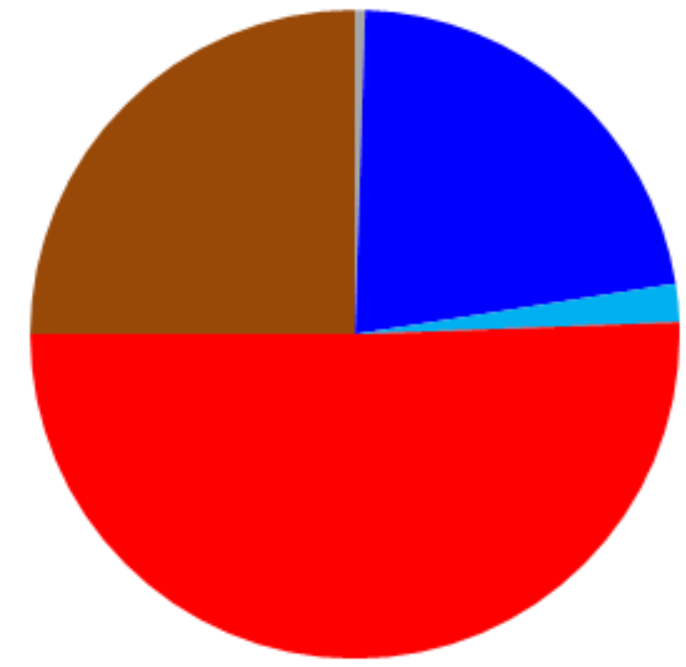

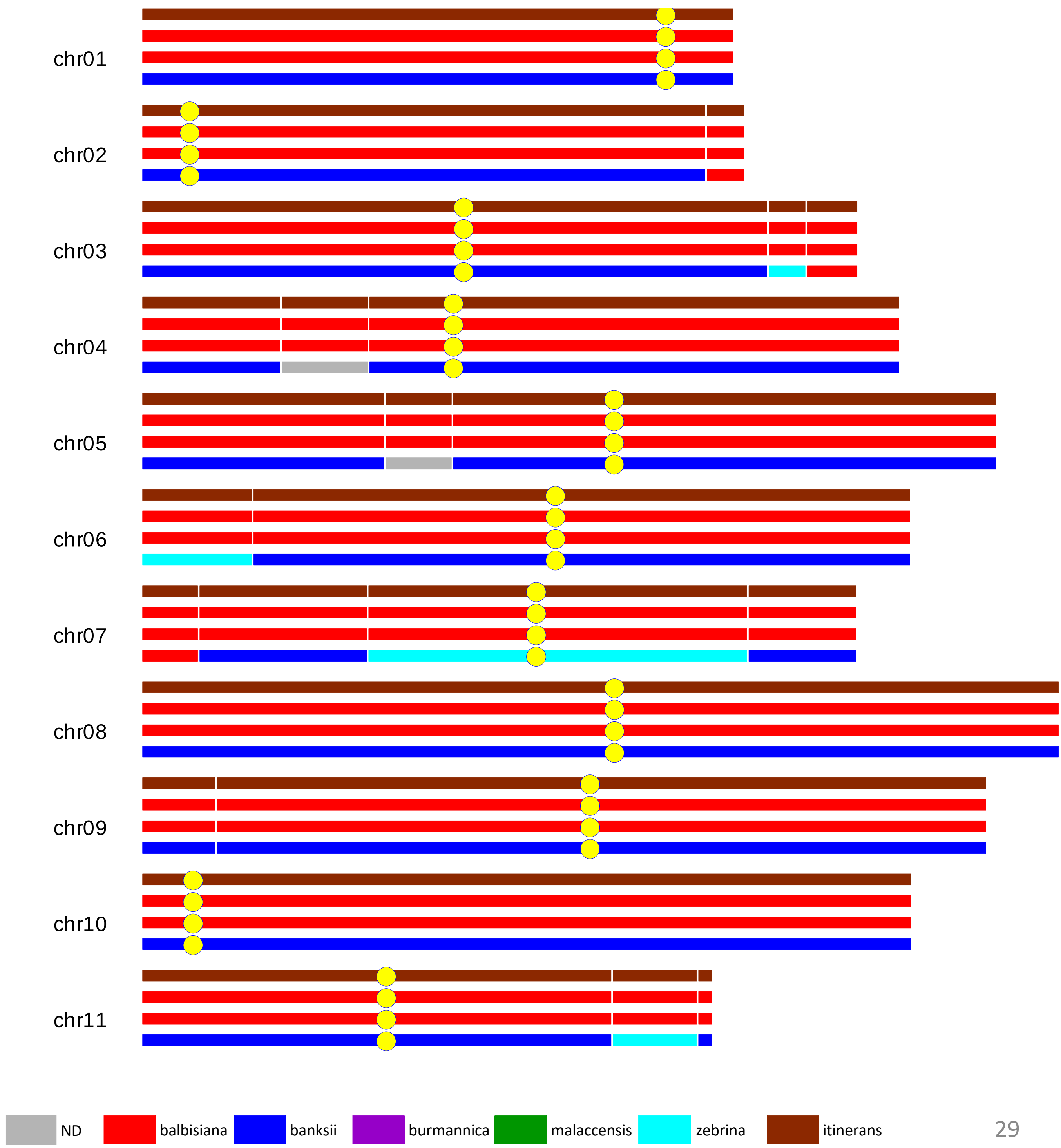

Group: ABBI type '77' variant '132'

- specimens 132 and 138 , road side in Vietnam
- specimen 272, backyard garden in Laos

(in grey dotted boxes, variations from type '77',
with a variation on chromosome 9 identical to that of
variant ABI 134 from type 28)

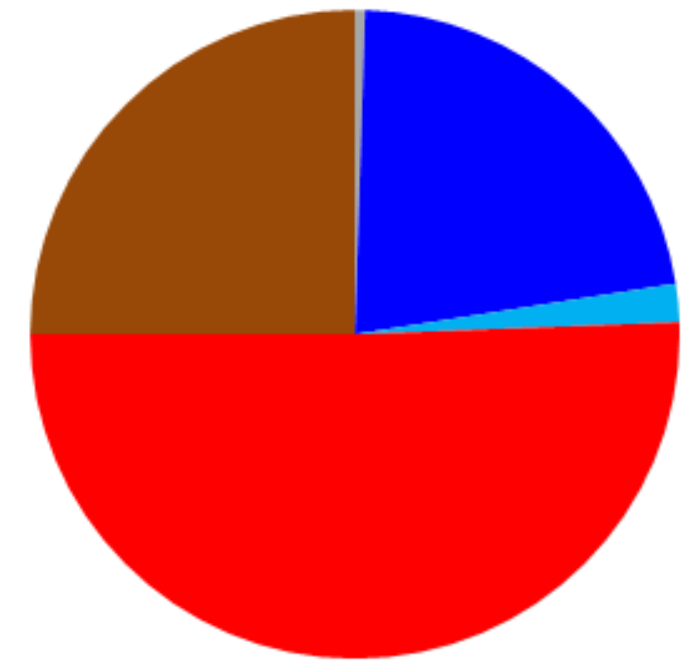

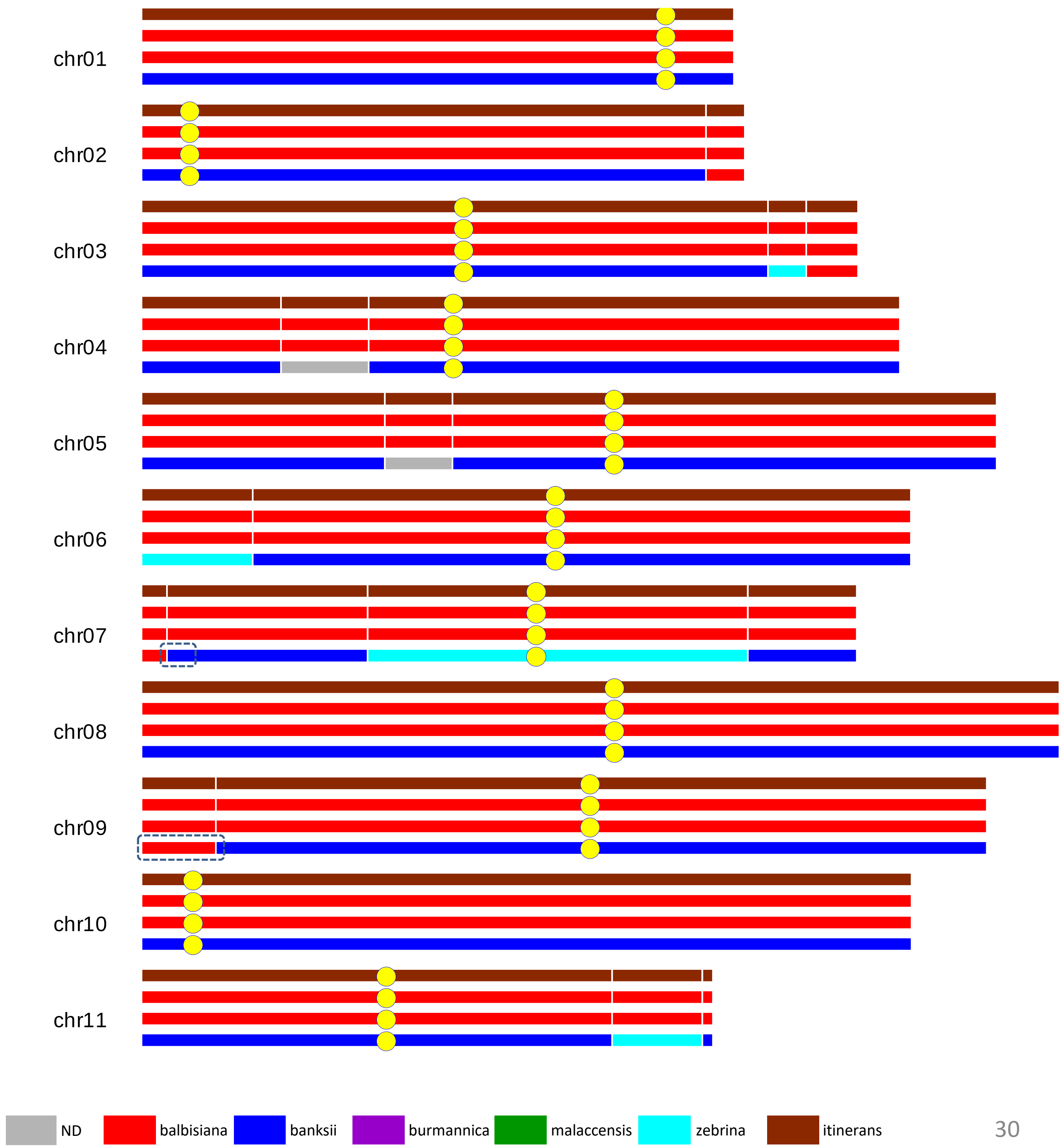

Group: AA Mchare

- reference 912, ‘Akondro Mainty’, from CRBP-PT in Guadeloupe (PT-BA-00010)

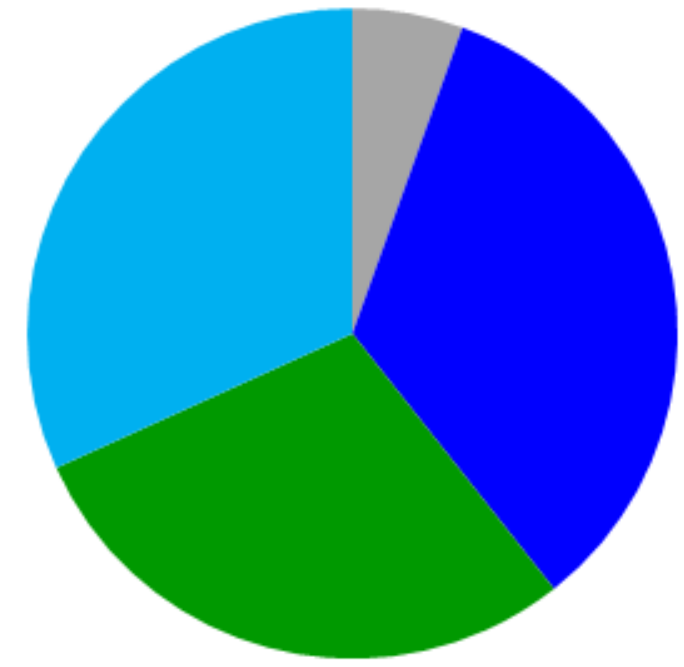

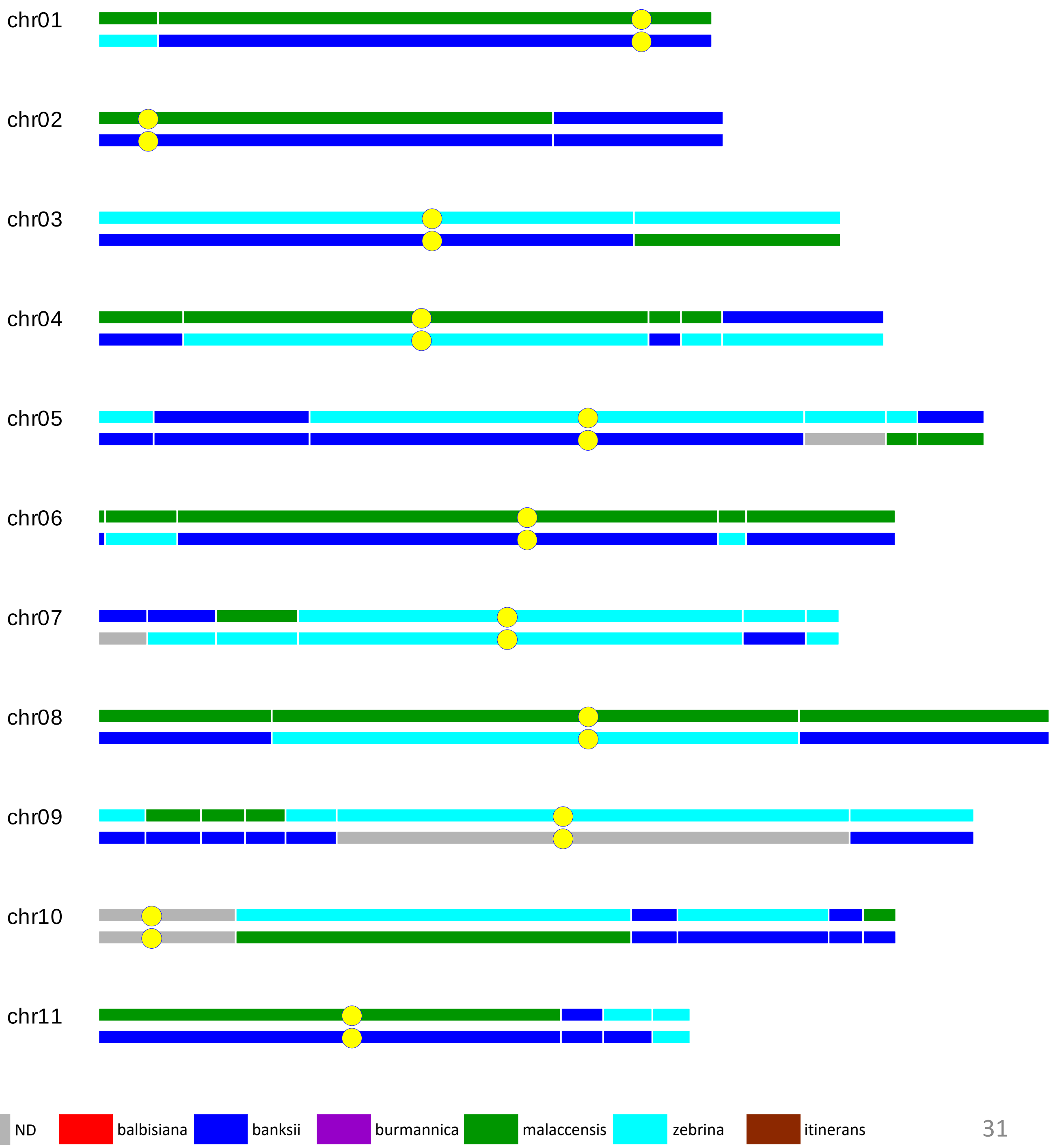

Group: AAA Gros-Michel

- reference 916, ‘Gros-Michel’, from CRBP-PT in Guadeloupe (PT-BA-00106)

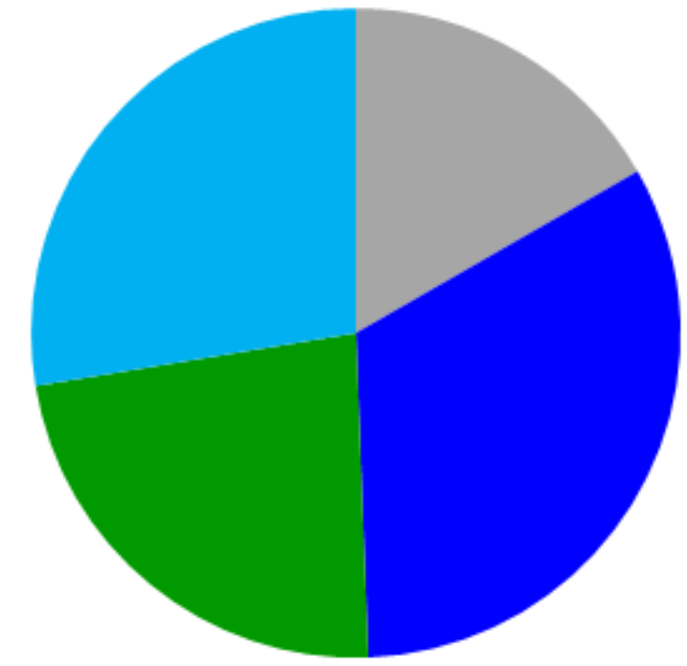

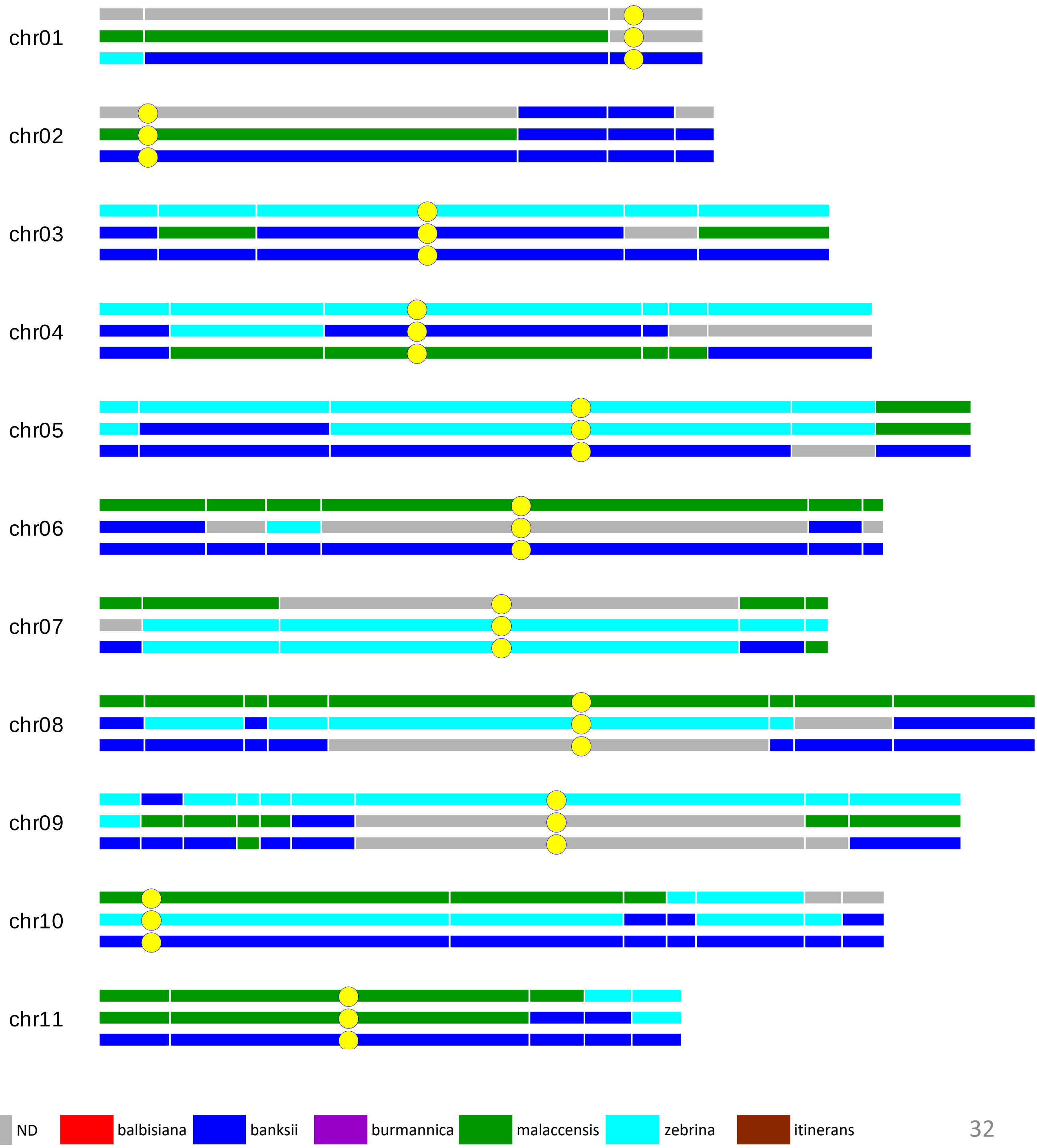

Group: AAA Mutika

- reference 917, 'Mbwazirume', from CRBP-PT in Guadeloupe (PT-BA-00201)

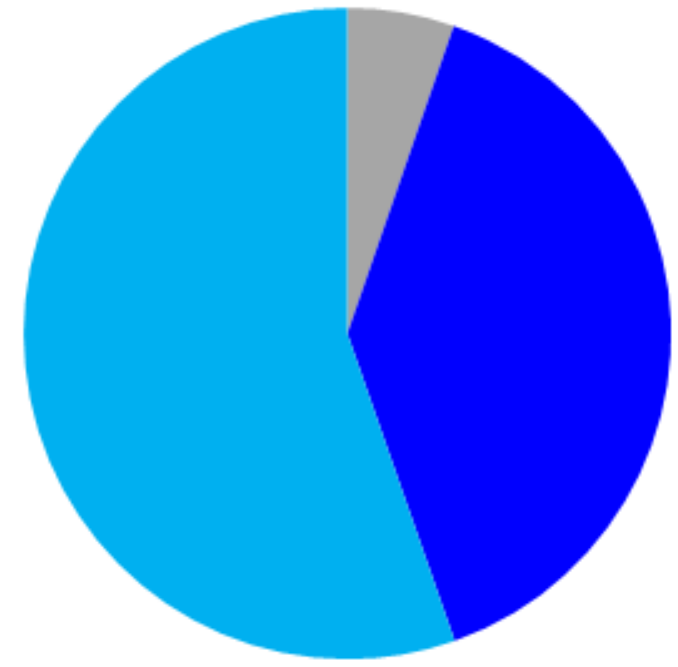

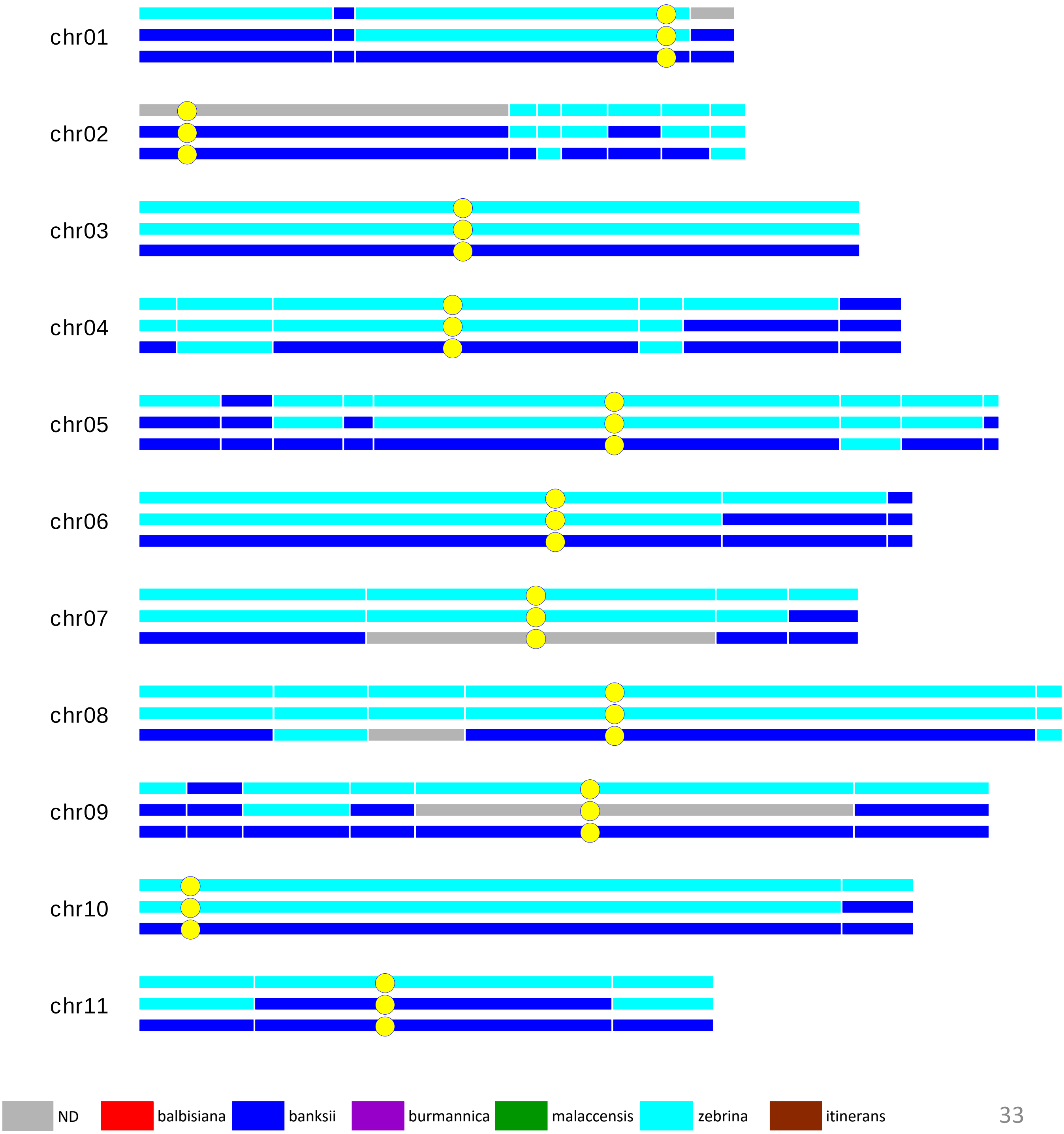

Group: AAB Silk

- reference 922, 'Kingala' from CRBP-PT in Guadeloupe (PT-BA-00153)

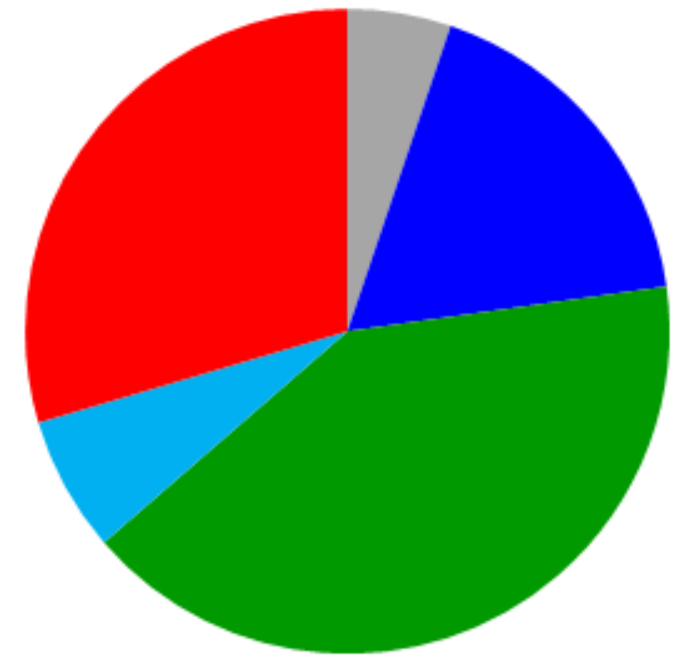

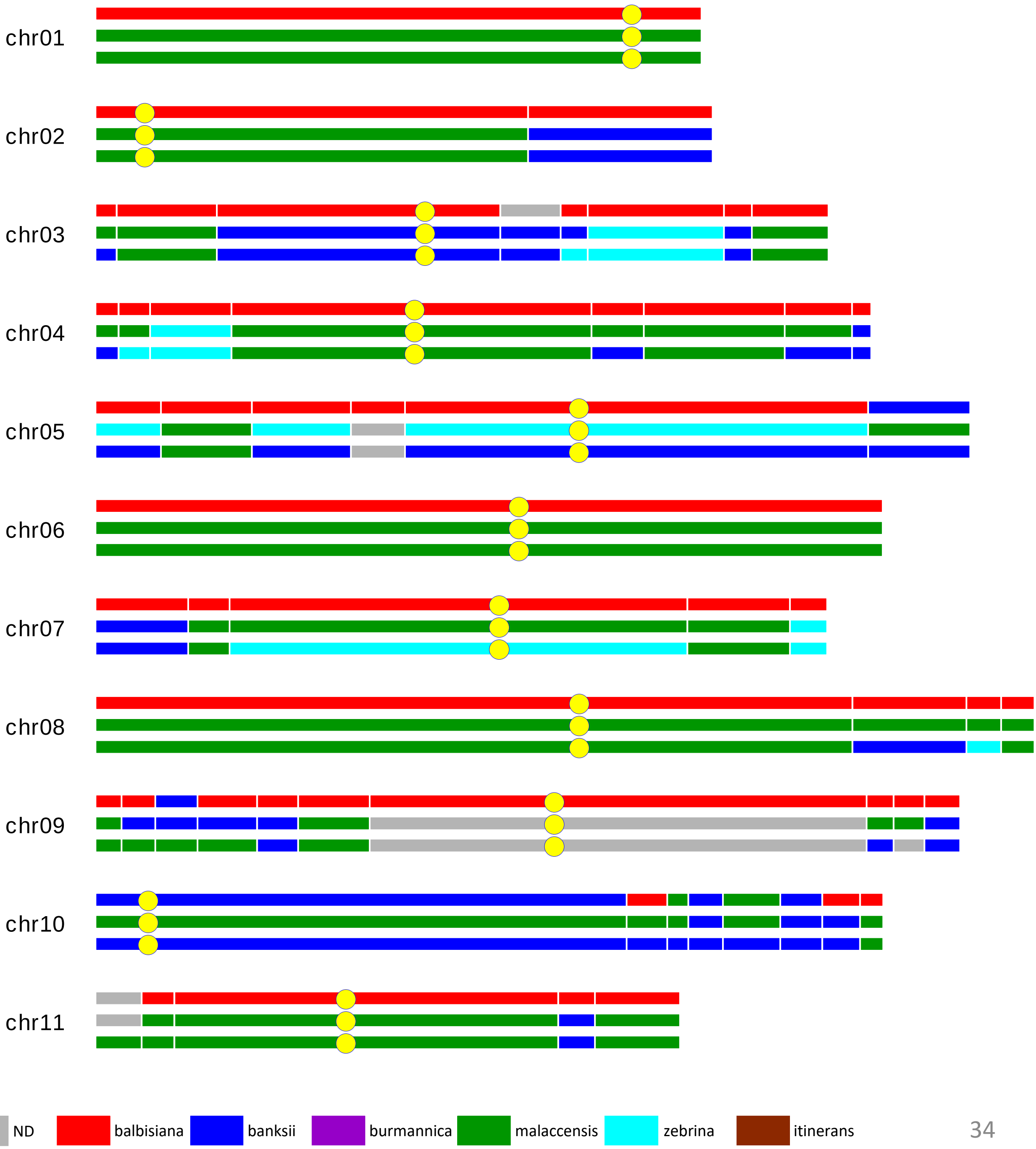

Group: AAB Laknao

- reference 919, ‘Laknao', from CRBP-PT in Guadeloupe (PT-BA-00171)

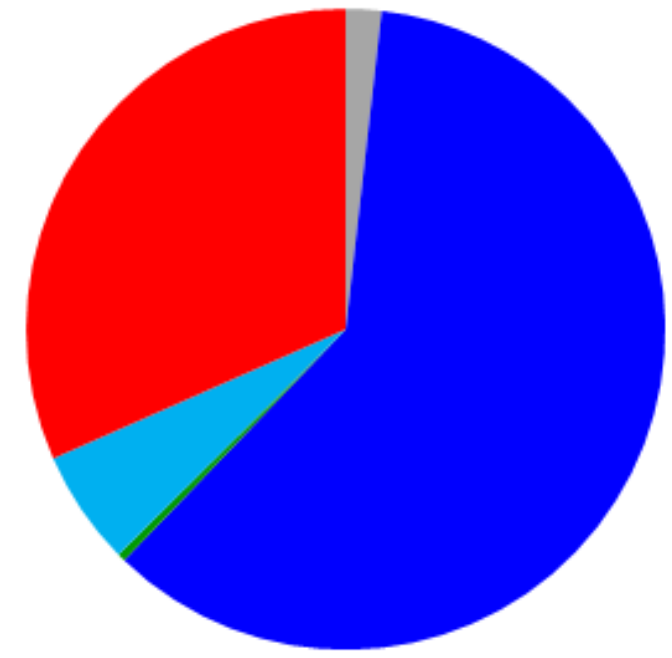

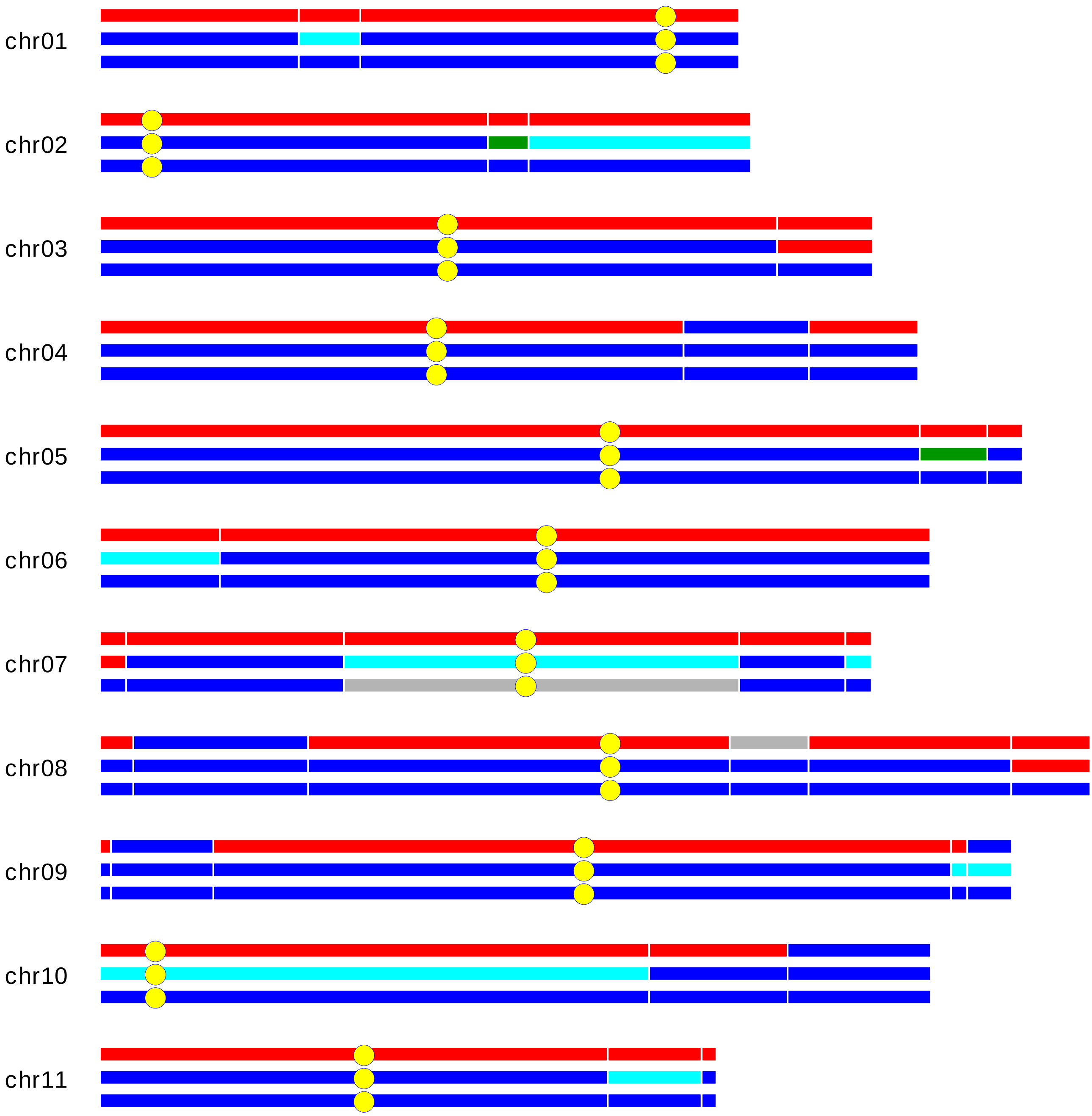

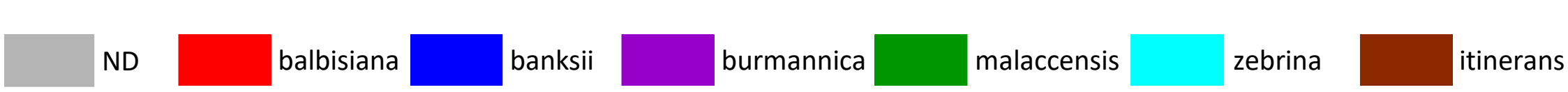

Group: AAB Iholena

- reference 918, ‘Luba', from CRBP-PT in Guadeloupe (PT-BA-00179)

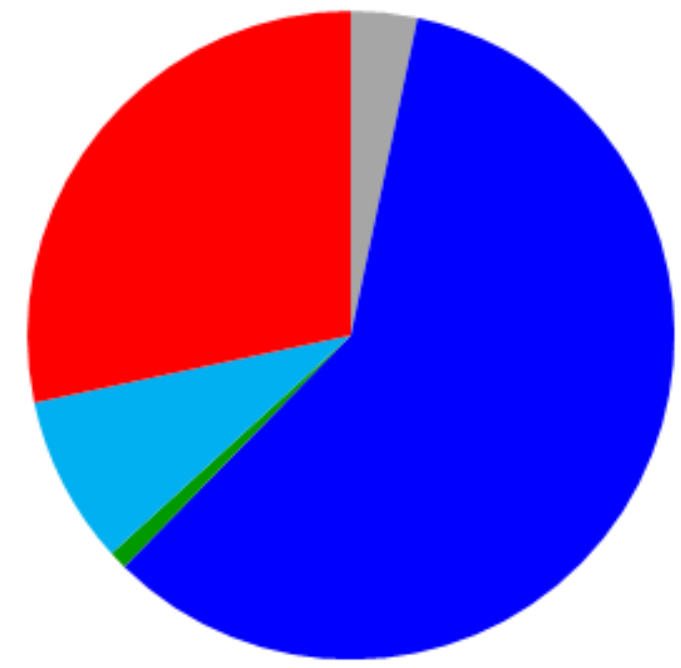

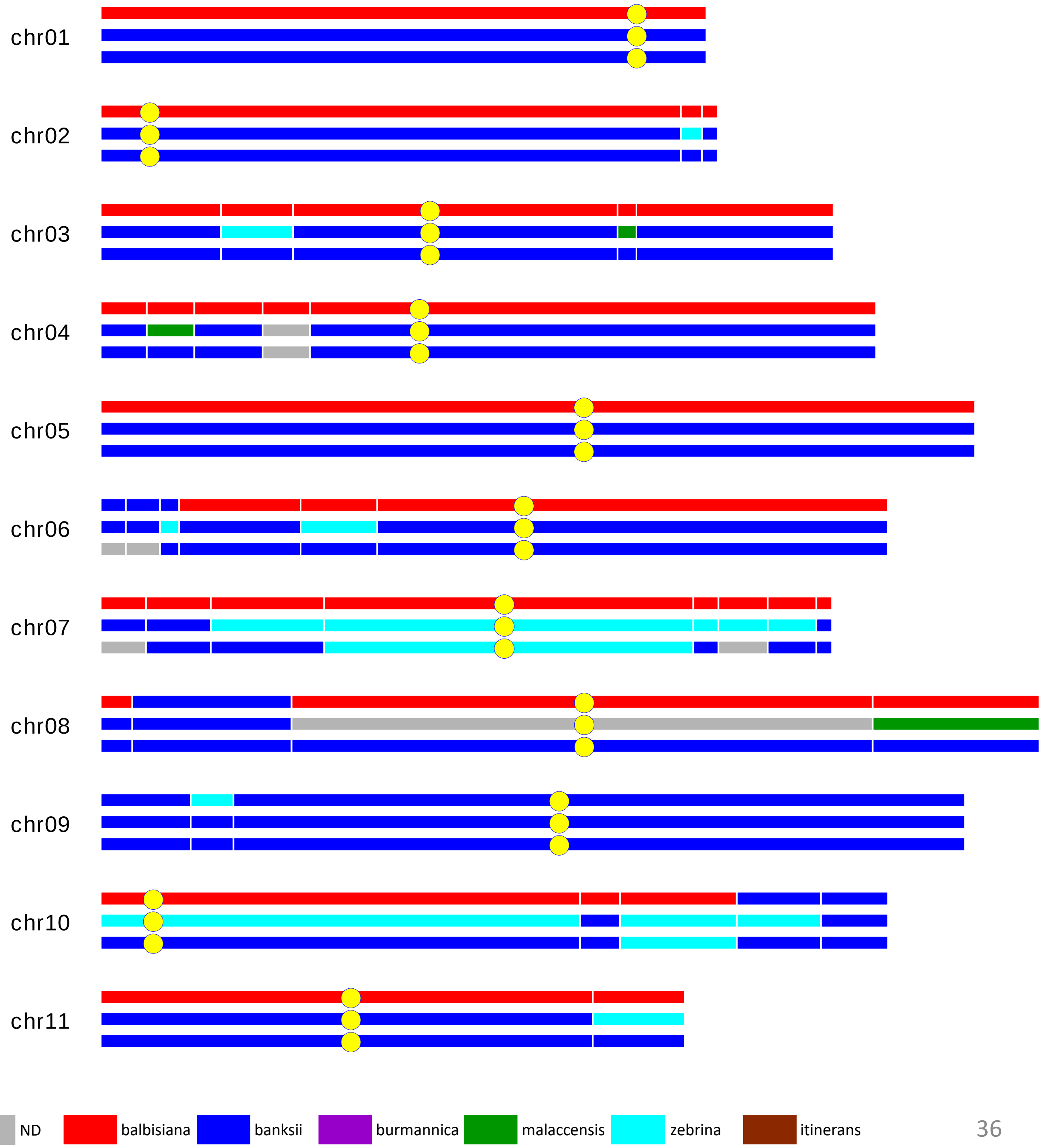

Group: AAB Popolou

- reference 923, 'Popoulou CMR', from CRBP-PT in Guadeloupe (PT-BA-00341)

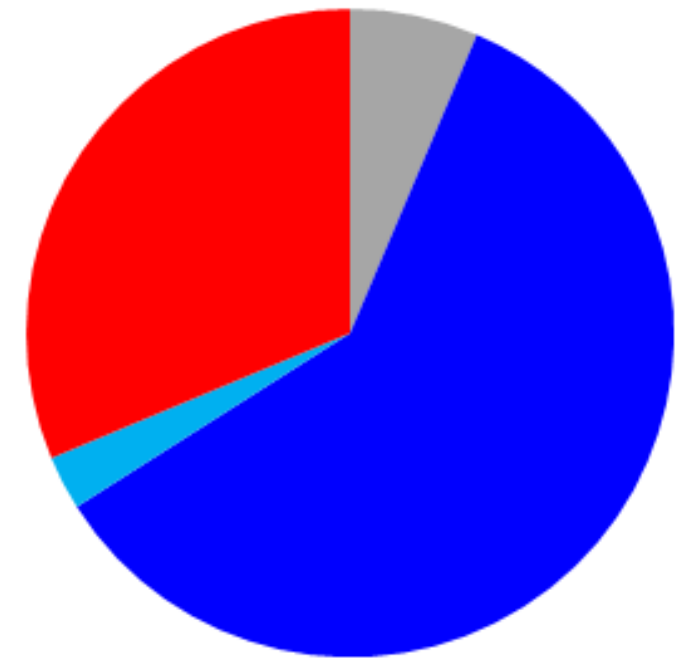

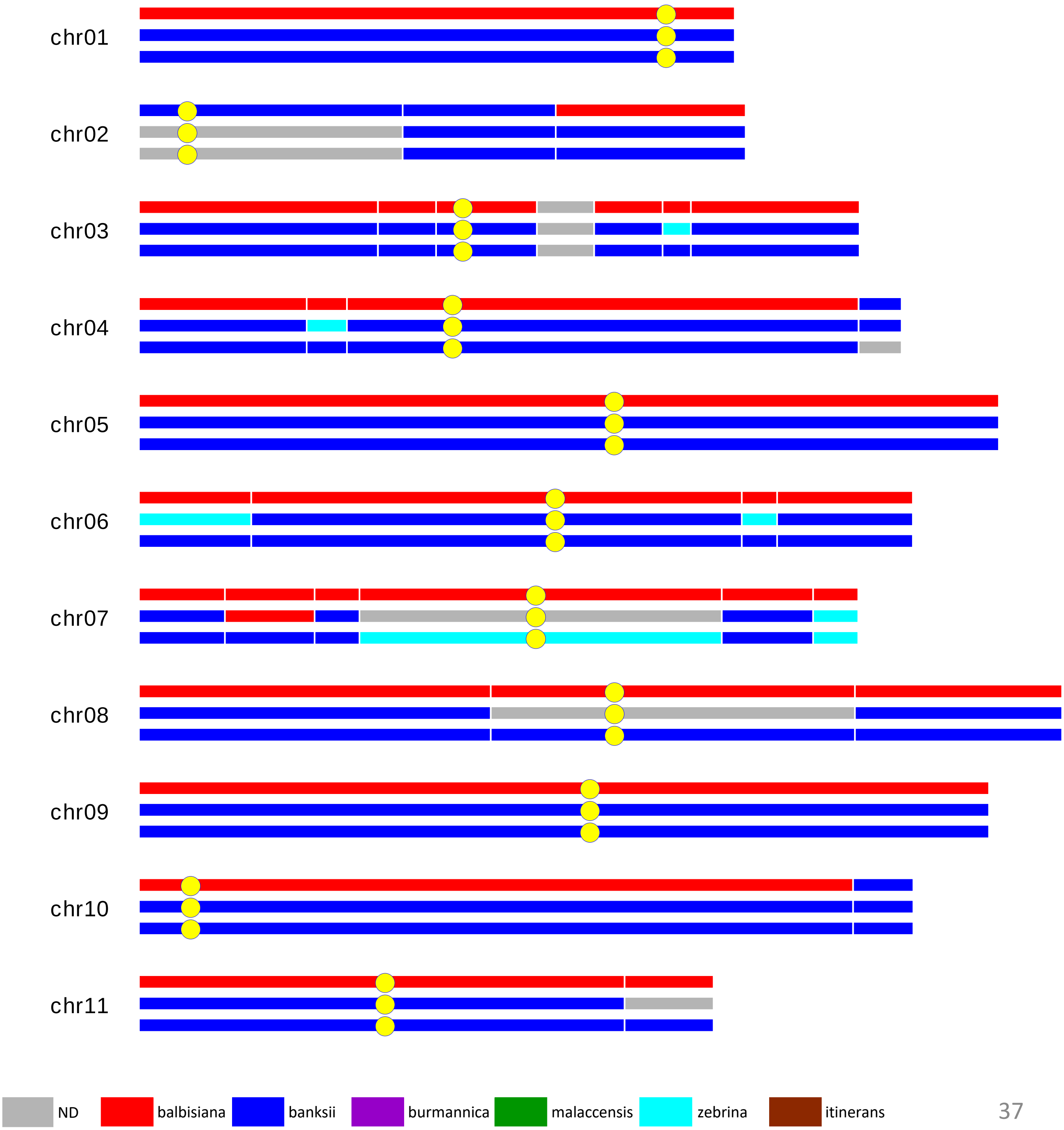

Group: ABB Saba

- reference 927, 'Saba’, from CRBP-PT in Guadeloupe (PT-BA-00358)

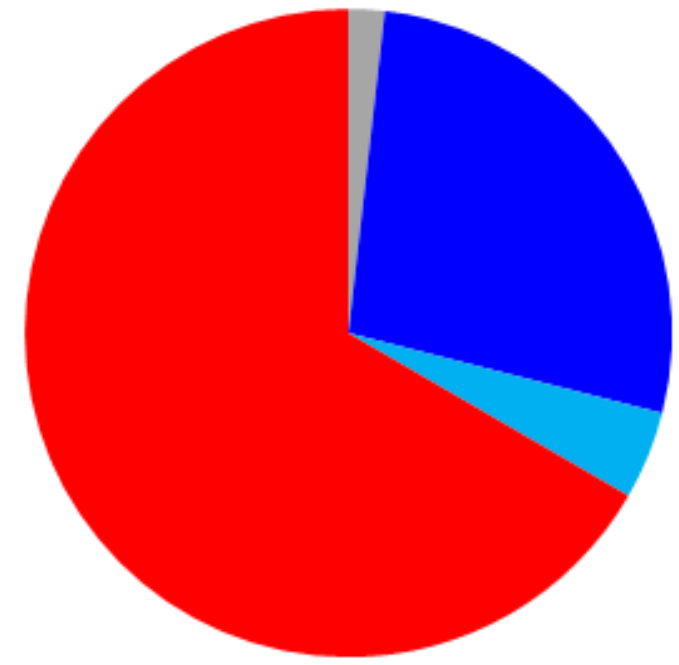

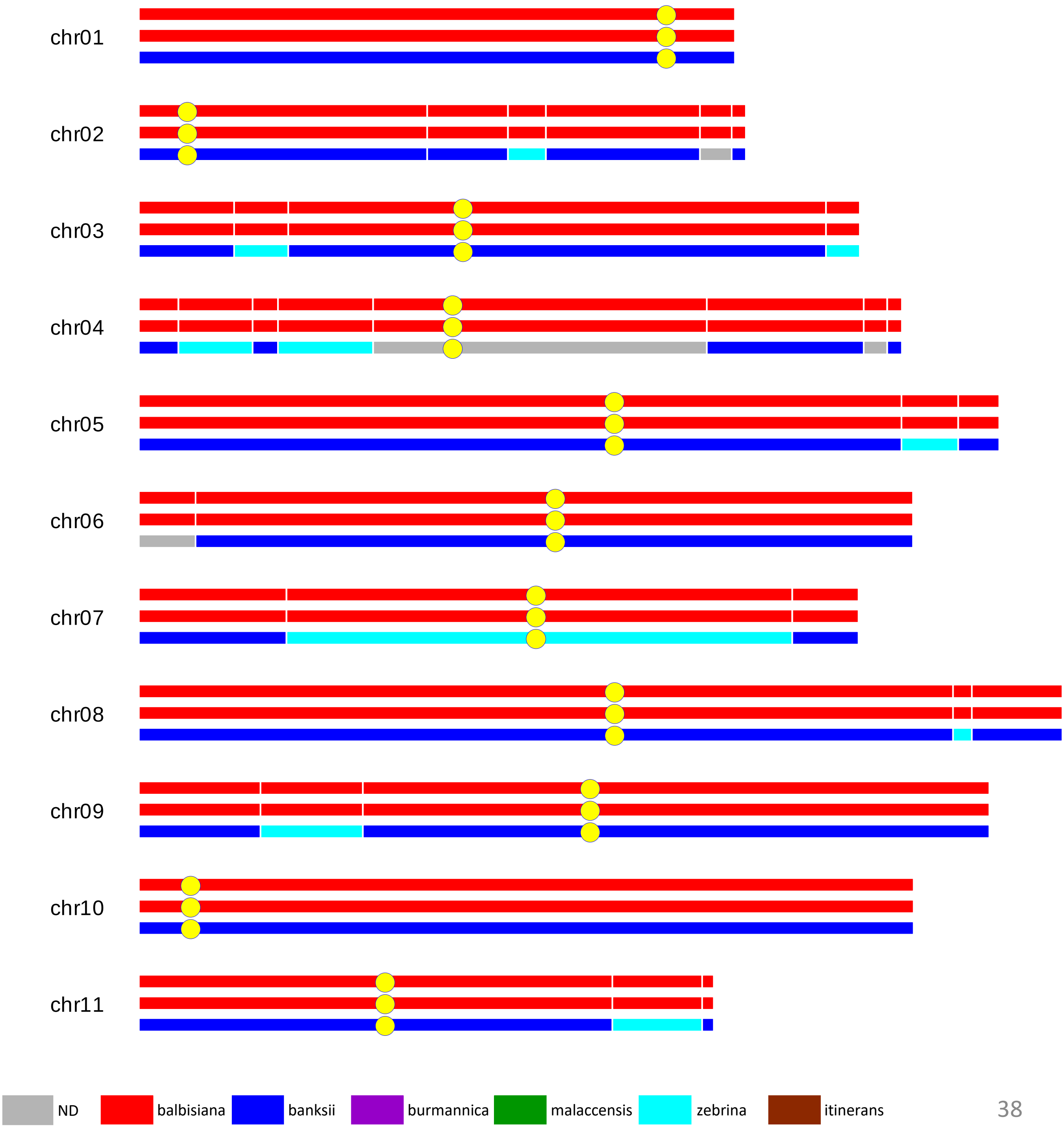

Supplement: Supplementary file 3 [file pone.0352247.s005.pdf]
